# Sex work or male-with-male sexual activity and the risks of HIV and hepatitis C acquisition among people who inject drugs: a global systematic review and meta-analysis

**DOI:** 10.1016/j.eclinm.2026.104268

**Published:** 2026-10-14

**Authors:** Joshua Dawe, Adam Trickey, Josephine Walker, Lucy Barrass, Belinda J. Njiro, Bidisha Kundu, Matthew Hickman, Daniela Abramovitz, Malvina Aladashvili, Irina Artamonova, Karine Blouin, Anders Boyd, Julie Bruneau, Kora DeBeck, Don Des Jarlais, Paul Dietze, Kostyantyn Dumchev, Catherine Hankins, Angelos Hatzakis, Jennifer R. Havens, Vivian Hope, Kanna Hayashi, Vita Jongen, Ann Kurth, Lisa Maher, Shruti Mehta, Holly Mitchell, Meghan D. Morris, Kimberly Page, Norah E. Palmateer, Lucy Platt, Maria Prins, Kasun Rathnayake, Lopamudra Ray Saraswati, Sotirios Roussos, Rachel Sacks-Davis, Avina Sarna, Sunil Solomon, Steffanie A. Strathdee, Vana Sypsa, Daniela Van Santen, Geng Zang, Jack Stone, Peter Vickerman, Adelina A. Artenie

**Affiliations:** a Bristol Population Health Science Institute, Bristol Medical School, Bristol, England, United Kingdom; b Health Protection Research Unit, Bristol Medical School, Bristol, England, United Kingdom; c Division of Infectious Diseases and Global Public Health, University of California San Diego, San Diego, USA; d AIDS and Clinical Immunology Research Center, Tbilisi, Georgia; e Institut National de Santé Publique du Québec, University of Montreal, Montreal, Canada; f Public Health Service of Amsterdam, Department of Infectious Diseases, Amsterdam, the Netherlands; g Department of Infectious Diseases, University of Bern, Bern, Switzerland; h Centre Hospitalier de l’Université de Montréal, CRCHUM, Montreal, Canada; i Département de Médecine de Famille et de Médecine d'urgence, Université de Montréal, Montreal, Canada; j School of Public Policy, Simon Fraser University, British Columbia, Canada; k New York University School of Global Public Health, New York University, New York, USA; l Disease Elimination, Burnet Institute, Melbourne, Australia; m Ukrainian Institute on Public Health Policy, Kyiv, Ukraine; n Department of Global and Public Health, McGill University, Montreal, Canada; o National and Kapodistrian University of Athens, Athens, Greece; p Department of Behavioral Science, University of Kentucky, Lexington, USA; q Public Health Institute, Liverpool John Moores University, Liverpool, England, United Kingdom; r United Kingdom Health Security Agency, London, England, United Kingdom; s Faculty of Health Sciences, Simon Fraser University, British Columbia, Canada; t New York Academy of Medicine, Yale University, CT, USA; u Viral Hepatitis Epidemiology and Prevention Program, Kirby Institute, Sydney, Australia; v John Hopkins Bloomberg School of Public Health, John Hopkins University, Baltimore, USA; w School of Medicine, University of California San Francisco, San Francisco, USA; x Department of Internal Medicine, University of New Mexico, Albuquerque, USA; y Sexual Health and Blood Borne Viruses Research Group, Glasgow Caledonian University, Glasgow, Scotland, United Kingdom; z Department of Public Health, Environments and Society, London School of Hygiene and Tropical Medicine, London, England, United Kingdom; aa Faculty of Public Health and Policy, London School of Hygiene and Tropical Medicine, London, England, United Kingdom; ab Amsterdam Institute for Immunology and Infectious Diseases, Amsterdam, the Netherlands; ac Department of Internal Medicine, University of Amsterdam, Amsterdam, the Netherlands; ad Jawaharlal Nehru University, Delhi, India; ae Population Council, Delhi, India; af School of Medicine, John Hopkins University, Baltimore, USA; ag School of Medicine, University of California San Diego, San Diego, USA

**Keywords:** HIV, Hepatitis C, People who inject drugs, Sex workers, Men who have sex with men

## Abstract

**Background:**

People who inject drugs (PWID) may engage in sexual behaviours that increase their HIV and hepatitis C virus (HCV) risk, including sex work and male-with-male sexual activity. We undertook a global systematic review to assess whether PWID who engage in these behaviours have higher HIV and HCV acquisition risks.

**Methods:**

In this systematic review and meta-analysis, we searched MEDLINE, Embase, and PsycINFO for studies among PWID published between 01/01/2000 and 09/06/2026 that included estimates of the association between male-with-male sex or sex work and our primary outcomes of HIV or HCV incidence. Studies that estimated incidence by longitudinally re-testing people at risk of infection or by using assays for recent infection were eligible for inclusion. We also contacted authors for unpublished estimates. We pooled estimates using random-effects meta-analysis. We used I^2^ to assess heterogeneity, and we used Egger's test to assess publication asymmetry. This review is registered with Prospero (CRD42020220884).

**Findings:**

We included 57 studies comprising 164 estimates. Among men, recent male-with-male sex was associated with higher HIV acquisition (pooled incidence rate ratio (IRR):2.35; 95% CI: 1.51–3.66; I^2^: 51%).) but not HCV (IRR: 1.28; 95% CI: 0.95–1.72; I^2^: 21%). Recent sex work had a 1.72-fold (95% CI: 1.30–2.28; I^2^: 65%) and 1.59-fold (95% CI: 1.29–1.96; I^2^: 60%) higher HIV and HCV acquisition risk among all PWID, respectively. When stratified, sex work was associated with higher HIV acquisition among women (IRR: 2.30; 95% CI: 1.44–3.68; I^2^: 68%) but not men (IRR: 1.43; 95% CI: 0.92–2.22; I^2^: 51%), and was associated with HCV acquisition among men (IRR: 1.51; 95% CI: 1.22–1.87; I^2^: 0%), but not women (IRR: 1.26; 95% CI: 0.90–1.77; I^2^: 55%). For lifetime behaviours, only male-with-male sex was associated with HIV acquisition (IRR: 2.44; 95% CI: 1.18–5.02; I^2^ = 87%). Results from Egger's tests did not provide evidence of asymmetry bias for our pooled estimates, while I^2^ measures suggested considerable heterogeneity for many outcomes.

**Interpretation:**

PWID who also engage in sex work or male-with-male sex were found to have higher HIV or HCV acquisition risks. Future research should aim to understand the mechanisms driving these associations.

**Funding:**

Medical Research Council Greater Western Four Partnership Doctoral Training Partnership, Wellcome Trust, Canadian Institutes of Health Research and Medical Research Council.

**Research in context**

**Evidence before this study**

People who inject drugs (PWID), sex workers, and men who have sex with men experience heightened risk of HIV and hepatitis C virus (HCV) acquisition and are therefore priority populations for health and harm reduction interventions. However, most research has examined these populations in isolation, and there is little understanding about how PWID who engage in overlapping sexual behaviours may have higher HIV and HCV acquisition risk.

We searched MEDLINE, Embase, and PsycINFO for articles published between January 1, 2000 and June 9, 2026, for systematic reviews on HCV or HIV incidence among PWID with the terms: (“Drug Users” [MeSH] OR “Substance Abuse, Intravenous” [MeSH] OR PWID [All Fields] OR PWIDs [All Fields] OR IDU [All Fields] OR IDUs [All Fields] OR IVDU∗[All Fields] OR “inject drug∗” [All Fields]) AND (“HIV” [MeSH] OR “HIV Infections” [MeSH] OR HIV [All Fields] OR “human immunodeficiency virus” [All Fields] OR “Hepatitis C” [MeSH] OR HCV [All Fields] OR “hepatitis c” [All Fields]) AND (“systematic review” [Publication Type] OR “meta-analysis” [Publication Type] OR “systematic review” [All Fields] OR “literature review” [All Fields] OR “meta-analysis” [All Fields] OR “meta analysis” [All Fields]), restricting our search to studies published within five years of our search. We identified four systematic reviews led by our collaborative group, in which two found strong associations between exposure to structural factors (incarceration and homelessness) and HIV and HCV acquisition risk and one found HIV and HCV acquisition risk was higher among women who inject drugs and younger PWID. We also identified a review estimating that around one in ten PWID identify as gay, lesbian or bisexual, and a similar percentage have recently engaged in sex work. However, to our knowledge, no review has yet assessed whether HIV and HCV acquisition risk is higher when PWID engage in sex work or male-with-male sexual activity.

**Added value of this study**

We conducted a global systematic review and meta-analysis, including published and unpublished estimates, to assess whether HIV and HCV acquisition risk is higher among PWID who engage in sex work or male-with-male sex compared to PWID who do not. Our study found that male PWID who recently engaged in male-with-male sexual activity experience more than two-fold higher rates of HIV acquisition compared with those who do not (pooled incidence rate ratio (IRR): 2.35; 95% CI: 1.51–3.66; number of estimates (N) = 13). Conversely, there was no evidence of an association between male-with-male sexual activity and HCV acquisition risk (IRR: 1.28; 95% CI: 0.95–1.72, N = 8). We also found higher rates of HIV (IRR: 1.72; 95% CI: 1.30–2.28; N = 20) and HCV (IRR: 1.59; 95% CI: 1.29–1.96; N = 22) acquisition linked to recent sex work among mixed-sex samples of PWID. In sex-stratified analyses, the association between recent sex work and HIV was strong among women (IRR: 2.30; 95% CI: 1.44–3.68; N = 12), but not among men (IRR: 1.43; 95% CI: 0.92–2.22; N = 12), while the association with HCV was strong among men (IRR: 1.51; 95% CI: 1.22–1.87; N = 14) but not among women (IRR: 1.26; 95% CI: 0.90–1.77; N = 12). Further, we found evidence of overlap in behaviours, with around one in nine PWID reporting recent engagement in sex work and around one in twenty male PWID reporting recent male-with-male sex.

**Implications of all the available evidence**

Our findings suggest many PWID engage in sex work or male-with-male sex which substantially increases their risk of HIV and HCV acquisition. Unfortunately, services providing care for populations at risk of HIV and HCV are frequently siloed, offering services that are focused on specific behaviours which are associated with greater HIV and HCV transmission risk. Our findings underscore the need for a more holistic and integrated approach, which recognises and addresses the multiple overlapping risks experienced by PWID. Such approaches could include integrated sexual health and harm reduction services, routine assessment of both injecting- and sexual-related risks and HIV/HCV prevention strategies that are tailored to individuals rather than behaviours. Further research is needed, with similar studies conducted in other settings, particularly LMICs, where evidence remains limited on how HIV/HCV risk varies among PWID with overlapping risk behaviours. In addition, across all settings, more data are needed on the underlying factors contributing to these elevated risks.

## Introduction

The sharing of injection equipment is a primary risk factor for hepatitis C virus (HCV) and HIV transmission, which are leading causes of morbidity and mortality for people who inject drugs (PWID).^1^^,^^2^ Globally, PWID experience high rates of new HIV and HCV infections,^3^^,^^4^ with contributing factors including suboptimal coverage of harm reduction interventions leading to heightened injecting and sexual risks and low coverage of HIV and HCV treatment.^5^^,^^6^ Further, structural barriers such as unstable housing and incarceration, which are commonly experienced by PWID, also increase HIV and HCV risk.^7^^,^^8^ Despite reductions in HIV and HCV incidence among PWID over the last two decades in some settings, transmission generally remains high,^3^ suggesting that the 2030 WHO and UNAIDS elimination targets are unlikely to be achieved among PWID without expanding prevention and treatment services, and reducing structural barriers.^3^^,^^4^

Beyond PWID, men who have sex with men (MSM) and sex workers also experience a disproportionate burden of HIV and HCV.^9^ Most HIV and HCV research among these groups treats them as distinct populations,^9^^,^^10^ while prevention and care interventions are organised around specific populations and behaviours.^11^, ^12^, ^13^ However, evidence suggests there is considerable overlap in sexual and injecting behaviours that are associated with greater risk of HIV and HCV transmission between PWID, MSM and sex workers.^5^^,^^14^ According to a recent global systematic review, 15% of PWID recently engaged in sex work and 9% identified as lesbian, gay, or bisexual.^5^

These overlapping behaviours may increase HIV and HCV exposure among PWID through compounding injecting and sexual risks, and heightened vulnerability to social and structural factors (e.g. stigma, marginalisation, poverty and criminalisation).^15^, ^16^, ^17^ For instance, compared with sex workers who do not inject drugs, those who inject drugs are more likely to engage in street-based or survival sex work,^18^^,^^19^ which is associated with limited condom negotiation, rushed or unsafe injections, more interactions with law enforcement and greater exposure to physical and sexual violence.^12^^,^^20^^,^^21^ Criminalisation of both sex work and drug use in most countries also subjects sex workers who inject drugs to intensified policing, and incarceration, which can disrupt health care access and increase HIV and HCV risk.^8^^,^^22^, ^23^, ^24^, ^25^ Gender dynamics compound these risks; globally, most sex work is conducted by women, who experience power imbalances, gender-based violence, and poverty.^26^, ^27^, ^28^ Similarly, MSM who use drugs are more likely to engage in condomless anal sex compared with MSM who do not use drugs^29^^,^^30^ and may participate in sexualised drug use (including ‘chemsex’),^31^ which can involve multiple partners and other sexual and injection behaviours that are associated with greater HIV and HCV acquisition risk.^11^^,^^32^^,^^33^ More broadly, experiences of stigma and discrimination related to sex work or sexuality^34^, ^35^, ^36^ can further influence these behaviours and deter access to health and harm reduction services.^37^^,^^38^

Despite evidence of overlapping sexual and injecting behaviours between PWID, sex workers and MSM, the extent to which the risks of HIV and HCV acquisition may differ among PWID who engage in sex work or male-with-male sex compared to those who do not is uncertain. We conducted a global systematic review and meta-analysis of published and unpublished data to estimate the magnitude of these risks.

## Methods

### Search strategy and selection criteria

This systematic review and meta-analysis uses our existing database of studies developed for four previous systematic reviews that utilised studies estimating HIV and/or HCV incidence among PWID.^3^^,^^4^^,^^7^^,^^8^ The original search covered the period from January 1st, 2000 to June 13th, 2017^8^ and included studies published in any language; it has been updated four times,^3^^,^^4^^,^^7^ most recently up to June 9th, 2026. The search strategy included terms related to injecting drug use, HIV infection, HCV infection, and study designs that can be used to estimate incidence (Appendix 3). The reference lists of relevant systematic reviews were searched. We reported this systematic review and meta-analysis in accordance with PRISMA (Preferred Reporting Items for Systematic reviews and Meta-Analyses) guidelines. A study protocol has been published (CRD42020220884).

Studies were eligible for inclusion in our database, maintained as an EndNote library, if they reported estimates of HIV or HCV incidence among PWID measured either through repeat testing of people at risk of infection or using indicators of recent infection (detectable RNA but not detectable antibodies for HCV or low-avidity/immature antibodies with high viral load for HIV). Studies which recruited participants in custodial settings were excluded. All studies included in this database had undergone title and abstract screening and full-text review to determine if they included HIV and/or HCV incidence estimates. For this review, all articles in the database were re-screened to assess whether they reported on the association between sex work (selling sex for money, food or drugs only) or male-with-male sex and risk of HIV or HCV acquisition. Full-text review of studies in our database were conducted by the first author (JD) and double-checked by co-authors (AA, JS). Google Translate was used to translate non-English papers.

In previous reviews using the same database,^3^^,^^4^^,^^7^^,^^8^ authors of published studies that reported HIV or HCV incidence among PWID were contacted to request relevant estimates if they were not included in any of their publications. Similarly, for this review, authors who had responded to earlier requests, as well as authors of newly identified papers, were contacted (by JD, PV, JW) to provide estimates of associations between male-with-male sex and/or sex work and HIV and/or HCV incidence if these were not available from published studies. When requesting unpublished estimates, we asked for both crude and adjusted estimates, with the latter adjusted for structural factors known to be associated with HIV and HCV risk (homelessness^7^ and incarceration),^8^ opioid agonist therapy (OAT) use,^39^ and specific injecting behaviours associated with higher HIV and HCV acquisition risk (injecting frequency and duration of injecting).^40^^,^^41^ The instructions for providing unpublished estimates are included in the Supplementary Material (Appendix 17). We use the term unpublished estimates to refer to estimates that were obtained by contacting authors. Although these estimates have not been previously published, they were calculated from cohorts which had previously published estimates of HIV or HCV incidence.

### Statistical analysis

Data from eligible studies were extracted by the first author (JD) using Microsoft Excel. Data extraction was double-checked by co-authors (AT, LB, BK, BN). Extracted data included HIV and HCV incidence, measures of association between sex work or male-with-male sex and HIV or HCV acquisition risk (sex-stratified where possible for sex work), publication year, years of data collection, study site, study design and the baseline characteristics of study participants. A detailed summary of the extracted data is available in Appendix 4. Studies reported either sex or gender, we therefore treated these terms as analogous for sex-stratified analyses. We did not conduct analyses among transgender people.

For each study, we extracted the reported measure of association (incidence rate ratio (IRR), hazard ratio (HR), or relative risk (RR)) comparing the risk of HIV or HCV acquisition among PWID reporting recent (up to past 12 months) or lifetime sex work vs those who did not and among those who reported recent or lifetime male-with-male sex vs those who did not. We distinguished between recent and lifetime exposure because recent exposure aligns more closely with the period at-risk for incident infections. For male-with-male sex, exposure was based on reported sexual behaviour. However, in studies where this was not available but data on sexual orientation were, we classified males who self-identified as gay or bisexual as having lifetime male-with-male sexual exposure. We also included studies reporting same-sex sexual activity among mixed-sex PWID samples when male-specific data were unavailable, provided that male PWID comprised >70% of the sample, with sensitivity analyses excluding these studies.

Measures of association were extracted separately by exposure type (sex work or male-with-male sex), exposure time frame (recent or lifetime), outcome (HIV or HCV acquisition), adjustment status (crude or adjusted), and, for sex work, by sex (male, female, or combined). Thus, each study could provide data on multiple exposure-outcome combinations (Appendix 5). If not reported, we calculated the measure of association and corresponding 95% CIs from raw data where possible. In studies with no incident HCV or HIV cases in either the exposed or unexposed group, we applied a continuity correction (i.e. 0.5 incident infections added to the numerator and denominator) to both groups to calculate a measure of association.^42^^,^^43^ When multiple publications reported on the same cohort for a given exposure and outcome, we included the one with the greatest total person-years at risk or sample size in our analysis. If the number of person-years or sample size was not reported or could not be estimated, we included the most recent publication.

Study quality was assessed using a modified version of the Newcastle–Ottawa Scale (score range 0–8), with lower scores indicating lower study quality (Appendix 8). In brief, the scale is used to score studies on three domains, namely whether the sample selection was representative of the target PWID population (max score of four), comparability between exposed and unexposed groups (i.e. whether confounders were adjusted for; max score of one); and ascertainment of the outcome (max score of three). We defined study quality as unsatisfactory if a study scored three or less, satisfactory if it scored four or five, good if it scored six or seven, and very good if it scored eight. Study quality was assessed by the first author (JD) and double-checked by co-authors (AA, JS, LB, AT, BK). For cohorts providing unpublished estimates, study quality was assessed independently by the study team using previously published protocols and manuscripts describing the same cohort, rather than being scored by the original investigators themselves. Where information required for quality assessment was not available in previous publications, study investigators were contacted directly to clarify relevant methodological details. We assessed potential publication asymmetry for all combinations of our exposures and outcomes using a funnel plot and Egger's test.

Estimates were pooled separately for each exposure-outcome combination (sex work or male-with-male sex as the exposure, and HIV or HCV acquisition as the outcome), the time frame of exposure (recent vs lifetime), and whether estimates were crude or adjusted for confounders. Random effects meta-analyses were used for pooling. We considered a pooled effect to have no statistical evidence of an association if the estimate crossed the null hypothesis. We also calculated the pooled proportion of participants involved in male-with-male sex and sex work for both recent and lifetime exposure using random-effects meta-analysis.

Measures of association were log-transformed for analyses and back-transformed for reporting. Our primary analyses focused on crude estimates for recent exposure to sex work or male-with-male sex, while secondary analyses focused on adjusted estimates and/or lifetime exposure. Analyses focused on sex work were conducted for samples of both males and females (mixed-sex samples), and then separately for samples stratified by sex (i.e. male-only and women-only samples). We primarily focused on crude estimates given that our aim was to establish whether PWID who also engage in male-with-male sex and sex work experience elevated HIV and HCV risk compared to those who do not, rather than to isolate the independent causal contribution of these behaviours themselves. Identifying whether such overlapping vulnerabilities are associated with excess risk is a necessary precursor to understanding the underlying mechanisms, whether driven solely by male-with-male sex and sex work, or by compounding structural and behavioural factors, which can then inform the design of targeted prevention efforts. Thus, crude estimates were chosen as the main analysis because they directly reflect the crude difference in HIV/HCV risk between the populations of interest: i) PWID who engage in sex work compared to those who do not, and ii) men who inject drugs who have sex with men compared to men who inject drugs who do not. In addition, adjusted estimates may control for factors that could lie on the causal pathway between these exposures and HIV or HCV acquisition (e.g. differences in injecting practices or sexual behaviours), potentially attenuating the effects. Crude estimates were also available for a larger number of studies and were more comparable across studies given variability in the selection of confounders between studies.

We assessed heterogeneity using the I^2^ statistic and Cochran's Q statistic. We conducted subgroup analyses to ascertain potential sources of heterogeneity for each exposure-outcome combination (sex work and male-with-male sexual activity in relation to HIV and HCV risks), restricted to analyses using recent exposure definitions and crude estimates. Factors considered in subgroup analyses included publication status, setting (high-income countries (HICs) vs low- and middle-income countries (LMICs)), mid-point of data collection (before 2010, 2010 or after), background incidence rates within each study (≥15 cases/100 PY for HCV, ≥2 cases/100 PY for HIV), method of incidence estimation, age of participants, proportion of PWID with recent incarceration, recent OAT use and recent homelessness, and the proportion of the sample that was female. Continuous variables were categorised at the median values of the studies included in each exposure-outcome analysis.

Data analysis was conducted in R (version 4.0.5) using the “meta” and “metafor” packages. Analysis code is available at https://github.com/JoshuaDaweUoB/PWID_SW_MSM_SR.

### Ethics

The study did not require ethical review or informed consent from participants of included studies, as it was a systematic review of published data, and unpublished data with prior ethical approval. The study was conducted according to the guidelines of the Declaration of Helsinki.

### Role of the funding source

This study received funding from the Wellcome Trust and the Canadian Institutes of Health Research and Medical Research Council. The funders of the study had no role in study design, data collection, data analysis, data interpretation, or writing of the publication.

## Results

Searches conducted as part of previous reviews and our update identified 82,105 studies published between January 1st, 2000, and June 9th, 2026, of which 27,279 were duplicates (Fig. 1). After screening titles and abstracts, 767 studies were eligible for full-text screening. Of these, 331 were excluded,^3^^,^^4^^,^^7^^,^^8^ with 436 studies reporting HIV or HCV incidence among PWID retained in our database. These 436 studies were full-text screened for this review, with 62 reporting at least one association between male-with-male sex or sex work and HIV or HCV incidence. After contacting authors of 36 studies to request additional data, a further 24 studies (24/36; 67% response rate) responded and provided data, resulting in a total of 86 studies. After removing studies that reported duplicate estimates from the same study population, 57 studies contributing 164 estimates (49 published and 115 unpublished) were retained. The updated search, which covered November 2nd, 2024 to June 9th, 2026, yielded one additional eligible study.

**Fig. 1:**
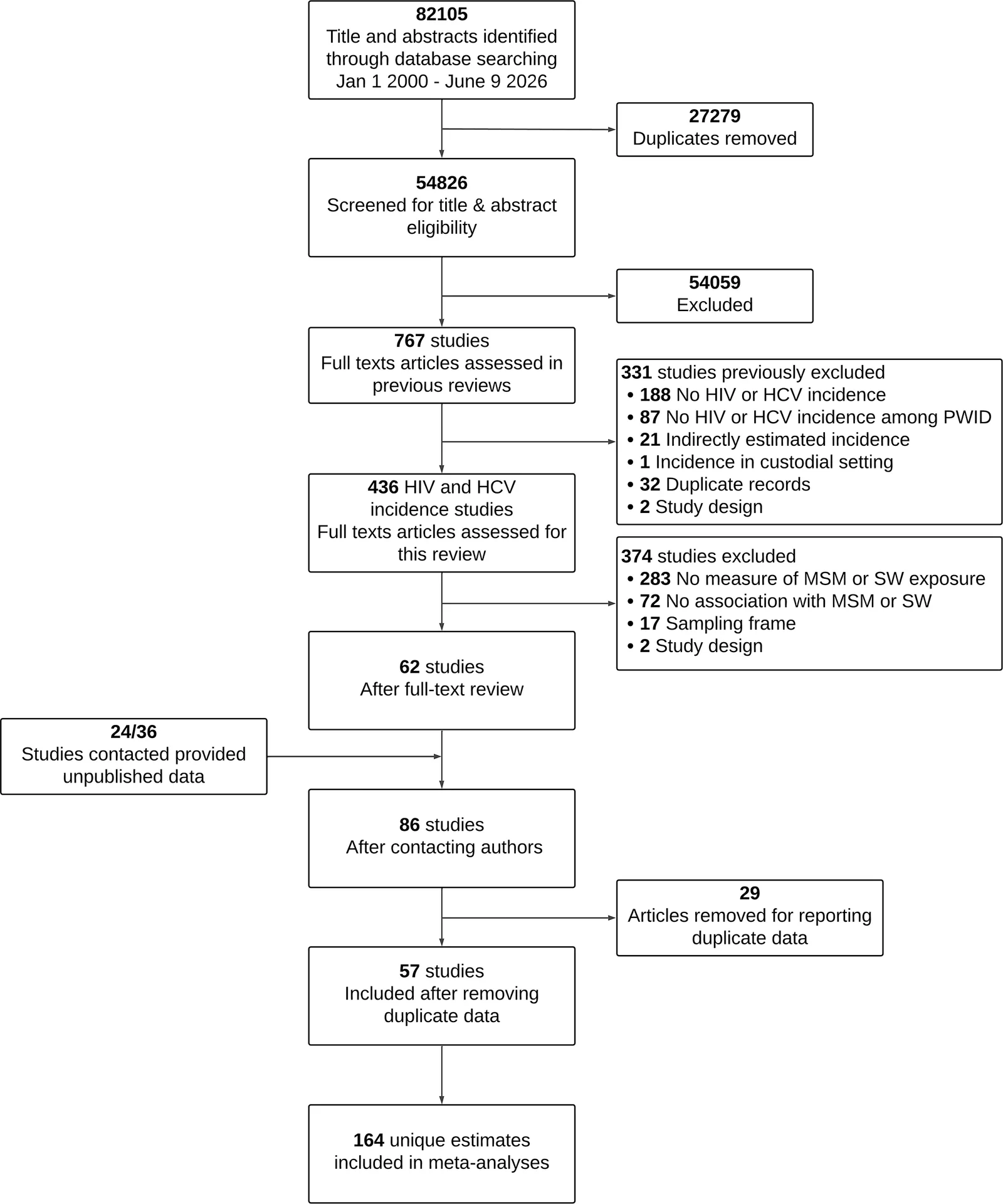
Study profile. HCV: hepatitis C virus; MSM: men who have sex with men; SW: sex work.

Among included studies, 23 reported estimates of association with HCV incidence, 22 reported estimates of association with HIV incidence, and 12 reported estimates of association for both HIV and HCV incidence. Twenty-four studies provided data on associations with sex work only, 11 on associations with sex between men only and 22 on associations with both exposures (Table 1).

**Table 1:** Summary of included studies.

Characteristic	Studies, n (%)	Estimates, n (%)
Overall	57	164
Publication status		
Published	33 (58)	49 (30)
Unpublished	24 (42)	115 (70)
Outcome		
HIV only	22 (39)	82 (50)
HCV only	23 (40)	82 (50)
Both HIV and HCV	12 (21)	–
Exposure		
Male-with-male sex only	11 (19)	42 (26)
Sex work only	24 (42)	122 (74)
Both male-with-male sex and sex work	22 (39)	–

HCV: hepatitis C virus.

In studies reporting associations between male-with-male sex and HIV or HCV acquisition risk (33 studies), recent and lifetime exposure were each reported in 17 studies. For HIV, all estimates were restricted to men who inject drugs. For HCV, three studies included estimates of same-sex sexual activity among participants who were not exclusively men (73%, 83% and 96% were men in each). In random-effects meta-analyses, the pooled proportions of men who inject drugs reporting recent and lifetime sex with men were 4.8% (95% CI: 2.7–8.3%; number of estimates (N) = 12; I^2^ = 98%) and 21.9% (95% CI: 11.9–36.8%; N = 14; I^2^ = 99%), respectively.

For HIV, 13 studies contributed crude estimates for the association between recent male-with-male sex and HIV acquisition risk among men who inject drugs, yielding a pooled incidence rate ratio (IRR) of 2.35 (95% CI: 1.51–3.66, N = 13), with moderate between-study heterogeneity (I^2^ = 51%; Table 2, Fig. 2). The pooled adjusted IRR was slightly higher than the crude IRR for recent male-with-male sex (IRR: 2.66; 95% CI: 1.43–4.98; N = 6; I^2^ = 71%). Lifetime male-with-male sex had a pooled crude IRR for HIV acquisition of 2.44 (95% CI: 1.18–5.02; N = 8, I^2^ = 87%), which was similar when using adjusted estimates (IRR: 2.32; 95% CI: 1.23–4.37; N = 8; I^2^ = 84%) (Appendix 10).

**Table 2:** Pooled estimates of the additional risk of HIV or HCV acquisition among men who inject drugs engaging in recent male-with-male sex.

	Number of estimates	Effect size (95% CI)	I^2^ (%)	P_heterogeneity_
**Effect on risk of HIV acquisition**				
Crude effect estimates	13	2.35 (1.51–3.66)	51.3	<0.001
Adjusted effect estimates	6	2.66 (1.43–4.98)	71.3	0.002
Crude subgroup analyses				
Published estimates	7	2.26 (0.97–5.30)	59.7	0.021
Unpublished estimates	6	2.08 (1.43–3.03)	17.5	0.300
Data collection midpoint <2010	6	2.96 (1.50–5.86)	61.3	0.024
Data collection midpoint ≥2010	7	1.83 (0.99–3.37)	38.6	0.134
HIV incidence ≥2 cases/100 PY	5	2.81 (1.36–5.82)	0.0	0.696
HIV incidence <2 cases/100 PY	7	2.62 (1.45–4.74)	66.0	0.007
HIV incidence unknown^a^	1	0.91 (0.37–2.27)	–	–
**Effect on risk of HCV acquisition**				
Crude effect estimates	8	1.28 (0.95–1.72)	21.0	0.263
Adjusted effect estimates	6	1.19 (0.76–1.86)	43.9	0.438
Crude subgroup analyses				
Published estimates	1	1.15 (0.67–1.96)	–	–
Unpublished estimates	7	1.28 (0.87–1.89)	30.2	0.198
Data collection midpoint <2010	3	1.31 (0.64–2.11)	0.0	0.510
Data collection midpoint ≥2010	5	1.16 (0.73–1.84)	46.8	0.111
HCV incidence ≥15 cases/100 PY	4	1.32 (1.01–1.73)	0.0	0.429
HCV incidence <15 cases/100 PY	2	1.55 (0.79–2.37)	32.2	0.224
HCV incidence unknown^a^	2	0.76 (0.04–15.55)	69.6	0.070

HCV: hepatitis C virus; CI: confidence interval; LIC: low income country; LMIC: low-middle income country; UMIC: upper-middle income country; UIC: upper income country; PY: person-years.

a Study incidence was not available in text.

**Fig. 2:**
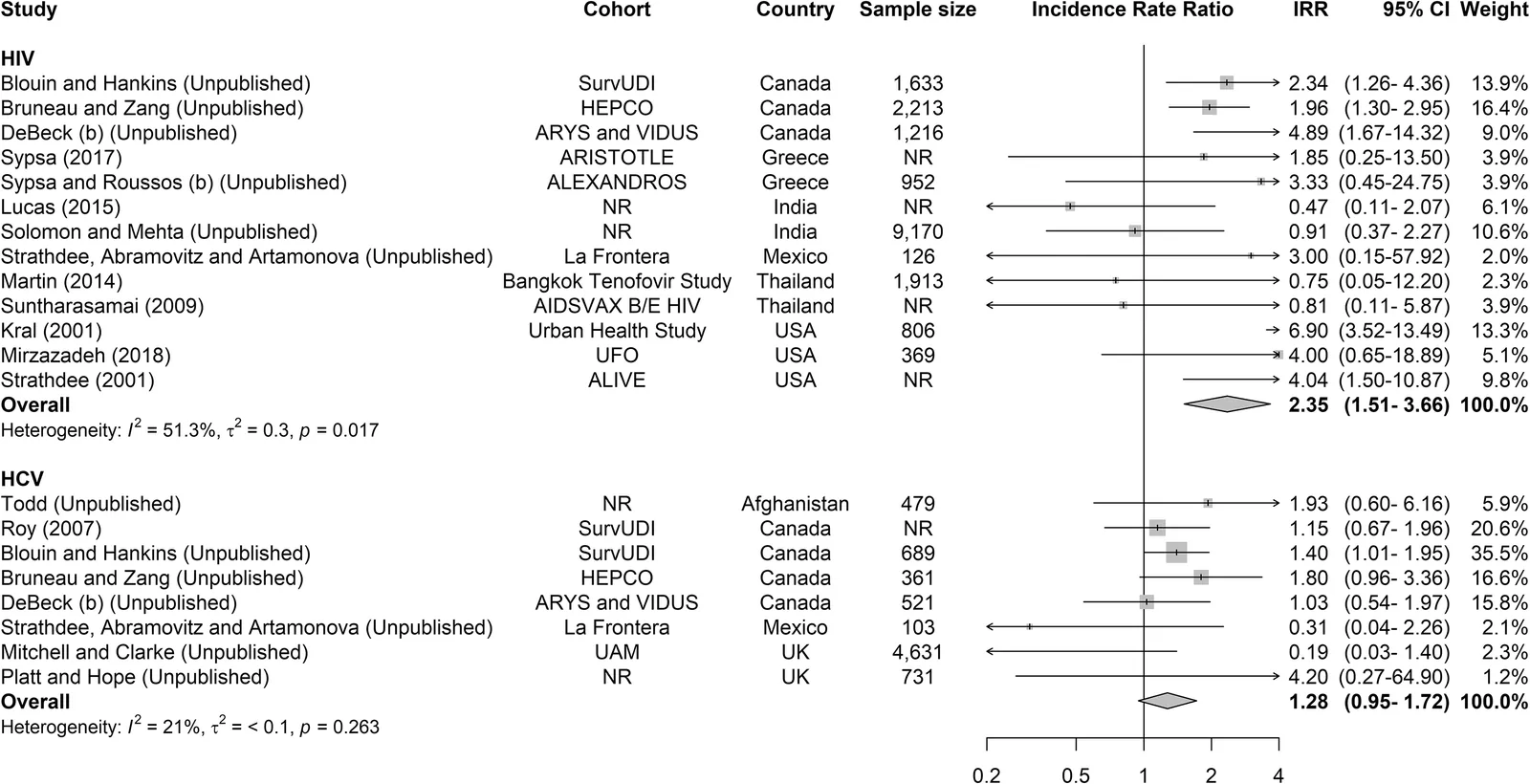
Pooled crude associations between recent male-with-male sexual activity and risk of HIV and HCV acquisition among people who inject drugs. HCV: hepatitis C virus; IRR: incidence rate ratio; CI: confidence interval.

Conversely, there was no evidence of an association between either recent (IRR: 1.28; 95% CI: 0.95–1.72; N = 8; I^2^ = 21%) or lifetime (IRR: 0.89; 95% CI: 0.74–1.07; N = 12; I^2^ = 0%) male-with-male sex and HCV acquisition risk when crude estimates were used (Table 2 and Appendix 10). Pooled estimates remained similar in sensitivity analyses where studies with fewer than 100% of the sample were male PWID were excluded (Appendix 13).

In studies reporting associations between sex work and HIV or HCV acquisition risk (N = 42), recent and lifetime exposure were reported in 38 and nine studies, respectively. In random-effects meta-analyses, the pooled proportions of PWID reporting recent and lifetime sex work were 10.6% (95% CI: 7.9–14.1%; N = 36; I^2^ = 99%) and 14.6% (95% CI: 8.8–23.2%; N = 9; I^2^ = 99%), respectively. By sex, these estimates were lower among men than women: 4.0% (95% CI: 2.5%–6.3%; N = 19; I^2^ = 98%) in men vs 27.2% (95% CI: 17.4%–39.8%; N = 15; I^2^ = 98%) in women for recent exposure, and 8.9% in men (95% CI: 4.5%–16.9%; N = 7; I^2^ = 99%) vs 44.0% in women (95% CI: 22.2%–68.4%; N = 5; I^2^ = 97%) for lifetime exposure.

Twenty studies contributed crude estimates for the association between recent sex work and HIV acquisition risk among male and female PWID combined, yielding a pooled IRR of 1.72 (95% CI: 1.30–2.28; N = 20) with moderately-high between-study heterogeneity (I^2^ = 63%, Table 3, Fig. 3). Pooled adjusted estimates were similar (IRR: 1.73; 95% CI: 1.27–2.36; N = 11; I^2^ = 59%). When crude estimates were stratified by sex, there was evidence of an association between recent sex work and HIV acquisition among women who inject drugs (IRR: 2.30; 95% CI: 1.44–3.68; N = 12; I^2^ = 68%), but no evidence among men who inject drugs (IRR: 1.43; 95% CI: 0.92–2.22; N = 12; I^2^ = 51%).

**Table 3:** Pooled estimates of the additional risk of HIV or HCV acquisition among PWID engaging in recent sex work.

	Number of estimates	Effect size (95% CI)	I^2^ (%)	P_heterogeneity_
**Associations with risk of HIV acquisition**				
Crude estimates	20	1.72 (1.30–2.28)	64.8	<0.001
Adjusted estimates	11	1.73 (1.27–2.36)	58.8	0.014
Male-only estimates^b^	12	1.43 (0.92–2.22)	50.7	0.022
Female-only estimates^b^	12	2.30 (1.44–3.68)	67.5	<0.001
Crude subgroup analyses				
Published estimates	10	1.79 (1.24–2.60)	65.9	0.002
Unpublished estimates	9	1.62 (0.98–2.69)	67.1	0.002
Data collection midpoint <2010	9	1.76 (1.24–2.50)	66.6	0.002
Data collection midpoint ≥2010	10	1.61 (0.96–2.70)	64.7	0.002
HIV incidence ≥2 cases/100 PY	8	1.24 (1.01–1.52)	0.0	0.511
HIV incidence <2 cases/100 PY	10	2.38 (1.80–3.16)	37.6	0.108
HIV incidence unknown^a^	2	0.49 (0.24–1.01)	0.0	0.400
**Associations with risk of HCV acquisition**				
Crude effect estimates	22	1.59 (1.29–1.96)	59.7	<0.001
Adjusted effect estimates	12	1.43 (1.13–1.80)	53.9	0.210
Male-only estimates^b^	14	1.51 (1.22–1.87)	0.0	0.986
Female-only estimates^b^	12	1.26 (0.90–1.77)	54.7	0.012
Crude subgroup analyses				
Published estimates	8	2.29 (1.79–2.93)	32.4	0.169
Unpublished estimates	14	1.29 (1.04–1.60)	37.4	0.077
Data collection midpoint <2010	10	1.98 (1.51–2.59)	65.5	0.002
Data collection midpoint ≥2010	12	1.19 (0.88–1.61)	40.8	0.069
HCV incidence ≥15 cases/100 PY	8	1.38 (0.98–1.95)	58.5	0.018
HCV incidence <15 cases/100 PY	8	1.78 (1.24–2.55)	79.0	<0.001
HCV incidence unknown^a^	6	1.45 (1.01–2.08)	0.0	0.626

HCV: hepatitis C virus; CI: confidence interval; LIC: low income country; LMIC: low-middle income country; UMIC: upper-middle income country; UIC: upper income country; PY: person-years.

Note: pooled effects combined males and females unless otherwise specified.

a Study incidence was not available in text.

b Studies could provide stratified data for males or females, data for males or females only, or combined males and females only.

**Fig. 3:**
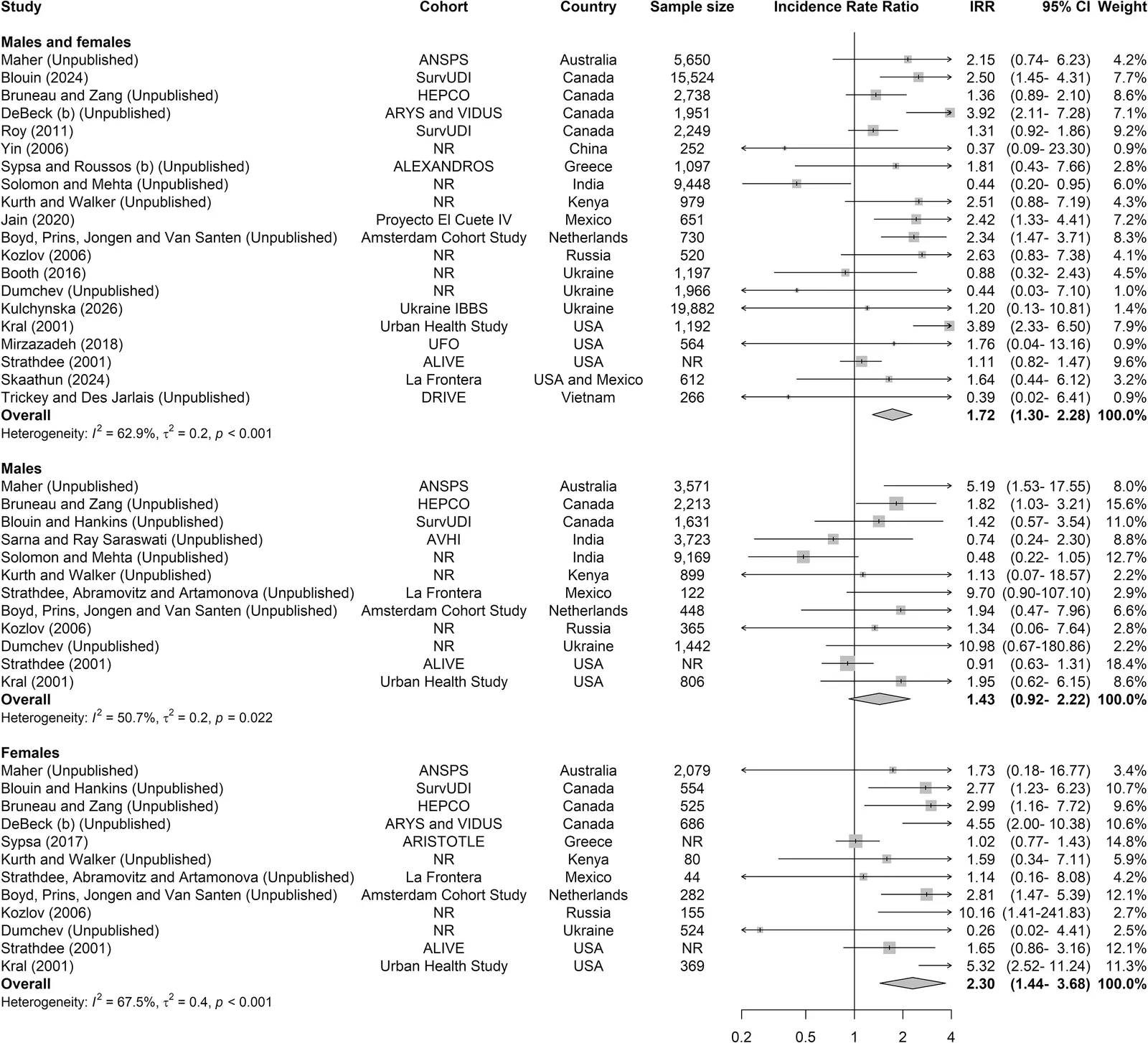
Pooled crude associations between recent sex work and risk of HIV acquisition among people who inject drugs, overall and by sex. IRR: incidence rate ratio; CI: confidence interval.

Recent sex work was also associated with HCV acquisition with a pooled IRR of 1.59 (95% CI: 1.29–1.96; N = 22) based on crude estimates, with moderately-high between-study heterogeneity (I^2^ = 60%, Table 3, Fig. 4). The association persisted, though slightly attenuated, when using adjusted estimates (IRR: 1.43; 95% CI: 1.13–1.80; N = 12; I^2^ = 54%). When crude estimates were stratified by sex, recent sex work was associated with HCV acquisition among men who inject drugs (IRR: 1.51; 95% CI: 1.22–1.87; N = 14; I^2^ = 0%), but not women who inject drugs (IRR: 1.26; 95% CI: 0.90–1.77; N = 12; I^2^ = 55%).

**Fig. 4:**
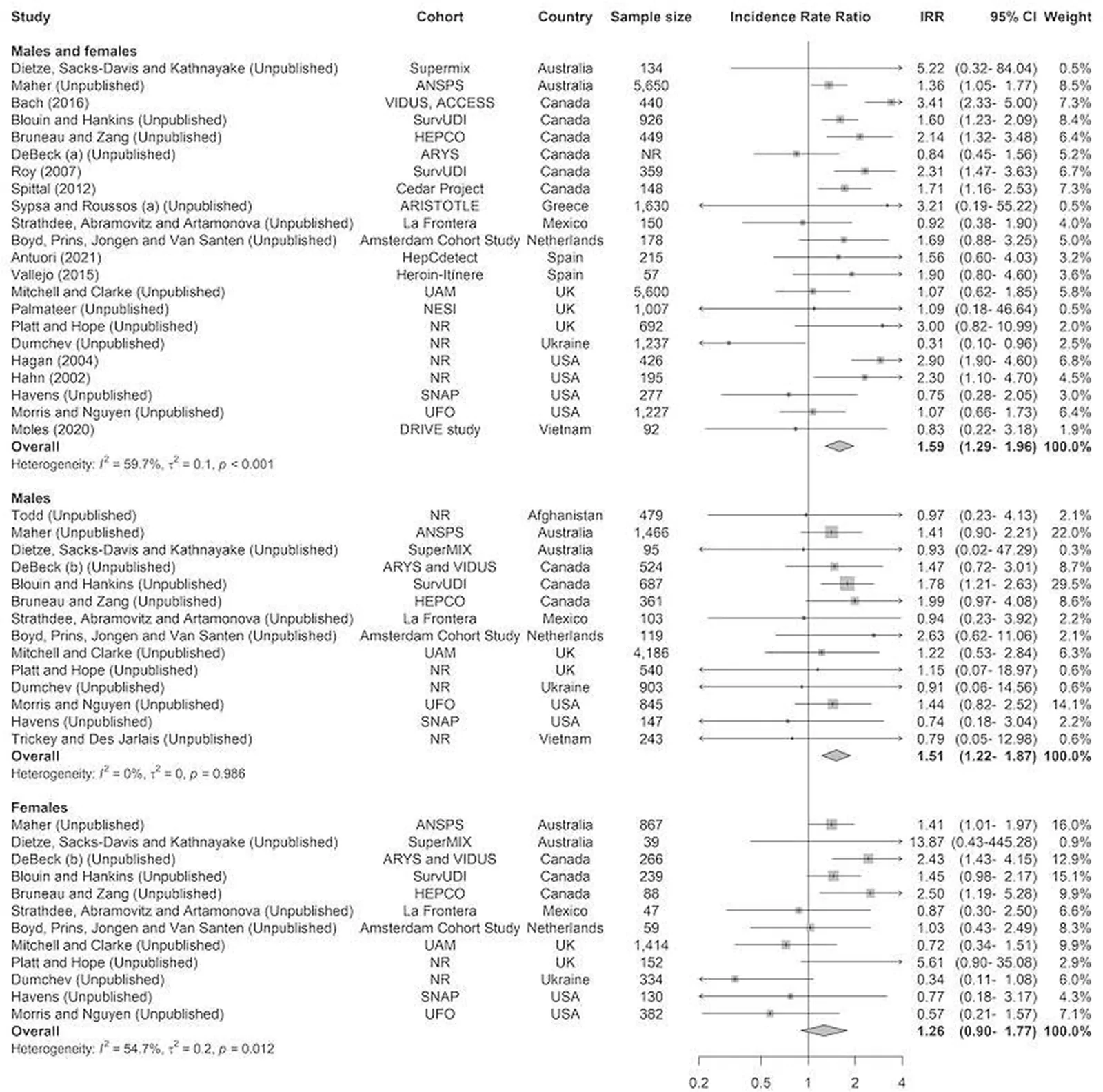
Pooled crude associations between recent sex work and risk of HCV acquisition among people who inject drugs, overall and by sex. HCV: hepatitis C virus; IRR: incidence rate ratio; CI: confidence interval.

There were fewer estimates of the association between lifetime sex work and HIV and HCV acquisition risk. When pooling crude estimates, we found no evidence of an association between lifetime sex work and HIV (IRR: 1.17; 95% CI: 0.76–1.79; N = 6; I^2^ = 52%) or HCV (IRR: 1.24; 95% CI: 0.95–1.63; N = 5; I^2^ = 7%) acquisition risk. Adjusted estimates also showed no evidence of an association with HIV (IRR: 1.10; 95% CI: 0.61–1.98; N = 4; I^2^ = 70%) or HCV (IRR: 1.21; 95% CI: 0.90–1.61; N = 4; I^2^ = 0%) acquisition.

Select sub-group analyses for male-with-male sex as the exposure are shown in Table 2 with complete results shown in Supplementary Material (Appendix 11 and Appendix 12). Subgroup analyses resulted in estimates that were largely similar with the main results across strata. However, we noted some differences. For the association between male-with-male sex and HIV acquisition risk, the magnitude of the association appeared larger in studies conducted in 2010 or later (vs earlier studies) and in HICs, whereas there was no clear association in LMICs. For male-with-male sex and HCV risk, an association was observed in studies with a shorter median duration of injection or a lower proportion of homeless PWID, whereas no clear association was evident in the corresponding studies with higher injecting duration or higher proportion homelessness.

For sex work and HIV risk, the magnitude of the association was greater in studies with a higher proportion of women, higher levels of recent incarceration and in HICs, whereas there was no clear association in studies from LMICs. For sex work and HCV risk, stronger associations were observed in published studies, in studies conducted before 2010, in HICs, and in studies with a lower proportion of homeless PWID. However, most pooled RRs across strata were based on small numbers of estimates, resulting in wide and overlapping 95% CIs.

When assessing differences between low- and high-incidence settings, we found no evidence that pooled effects differed between male-with-male sex and HIV incidence (<2 cases/100 PYs: [RR: 2.62; 95% CI: 1.45–4.74; N = 7; I^2^ = 66%] vs ≥2 cases/100 PY: [RR: 2.81; 95% CI: 1.36–5.82; N = 5; I^2^ = 0%]) and HCV incidence (<15 cases/100 PYs: [RR: 1.55; 95% CI: 0.79–2.37; N = 2; I^2^ = 70%] vs ≥15 cases/100 PY: [RR: 1.32; 95% CI: 1.01–1.73; N = 4; I^2^ = 0%]). For sex work, we found substantially higher pooled effects in low incidence studies for HIV (<2 cases/100 PYs: [RR: 2.38; 95% CI: 1.80–3.16; N = 10; I^2^ = 38%] vs ≥2 cases/100 PY: [RR: 1.24; 95% CI: 1.01–1.52; N = 8; I^2^ = 0%]), and slightly elevated pooled effects in low incidence studies for HCV incidence (<15 cases/100 PYs: [RR: 1.78; 95% CI: 1.24–2.55; N = 8; I^2^ = 79%] vs ≥15 cases/100 PY: [RR: 1.38; 95% CI: 0.98–1.95; N = 8; I^2^ = 59%]).

Visual inspection of funnel plots and the results from Egger's tests did not provide any evidence of asymmetry bias for our pooled estimates (Appendix 14). This finding was mostly consistent when assessing published and unpublished estimates separately, except we found evidence of asymmetry for published studies assessing the association between male-with-male sexual activity and HIV acquisition (P = 0.011).

## Discussion

In this global systematic review and meta-analysis, recent sex work and male-with-male sexual activity were associated with higher HIV acquisition risk among PWID. Male PWID who engaged in recent male-with-male sex had a 2.3-fold higher risk of HIV acquisition. Similarly, PWID engaged in recent sex work had a 1.7-fold higher risk of HIV acquisition, with this risk being higher among women who inject drugs (2.3-fold higher risk), but there being no evidence among men who inject drugs. In contrast, evidence regarding recent male-with-male sex or sex work and the association with HCV acquisition risk was weaker. There was no evidence that PWID reporting recent male-with-male sex had a higher risk of HCV acquisition. However, among PWID who recently engaged in sex work, there was evidence of a 1.6-fold increase in HCV acquisition risk, which persisted when considering men only (1.5-fold higher risk) but there being no evidence among women. These findings, based on crude estimates for recent exposure to male-with-male sex and sex work, were consistent when considering adjusted estimates. For lifetime exposure, findings were similar for the association between HIV acquisition risk and male-with-male sexual activity, but there was no evidence of an association for HCV acquisition risk or for exposure to sex work. Notably, the point estimates of pooled effects in which 95% confidence intervals crossed the null still suggest a higher risk of HIV and HCV acquisition among PWID engaging in male-with-male sexual activity or sex work, possibly reflecting a lack of statistical power rather than a lack of effect, given that we would expect these behaviours to elevate risk.

Previous studies have generally treated sex workers, MSM, and PWID as distinct populations when describing transmission risks and their burden of HIV and HCV.^9^ Our review is novel in quantifying the extent to which PWID who engage in male-with-male sex or sex work experience an increased risk of HIV and HCV acquisition compared to PWID without these behaviours.^3^^,^^5^^,^^44^, ^45^, ^46^, ^47^, ^48^, ^49^, ^50^ While our analyses cannot directly identify the mechanisms driving these associations, our findings might be explained by higher levels of stigma, discrimination, and criminalisation among PWID who engage in sexual and injecting behaviours that increase HIV and HCV acquisition risk, though clear data supporting this remain limited.^24^^,^^51^ These factors are, in turn, associated with reduced access to health and harm reduction services.^52^^,^^53^ These associations should also be interpreted in the context of underlying transmission dynamics for HIV and HCV. For example, the probability of HCV transmission is higher for parenteral exposure compared with sexual exposure, which may explain the lack of an association between male-with-male sex and HCV acquisition. The association between sex work and HCV acquisition may therefore be attributable to structural risks such as unstable housing, criminalisation, and reduced access to harm reduction services which are associated with increased injecting behaviours that increase HIV and HCV acquisition risk.^8^^,^^54^^,^^55^ For HIV, where sexual transmission is more efficient, the elevated risks observed for both male-with-male sex and sex work likely reflect a combination of direct sexual transmission risk, with significant a HIV burden among MSM and people engaged in sex work, and structural vulnerabilities that concurrently elevate injecting-related behaviours that increase HIV and HCV acquisition risk.

Furthermore, our study design does not allow us to distinguish between sexual and injecting transmission. Thus, a better understanding of the underlying mechanisms is needed to help inform intervention development. Initiatives to address these elevated risks should include the development of integrated, low-threshold sexual health and harm-reduction services alongside policy changes to reduce criminalisation, stigma, and barriers to care. This is particularly important given how common overlapping sexual exposures are among PWID, with our study finding that 11% of PWID reported recent sex work, while approximately 5% of male PWID reported recent male-with-male sexual activity and around 22% reported lifetime male-with-male sexual exposure. Recent evidence from global systematic reviews suggests that HIV and HCV incidence is higher for women who inject drugs compared with men,^3^ likely reflecting the structural vulnerabilities that women who inject drugs can experience, including gender-based violence and unequal power dynamics.^56^ Importantly, our analyses also suggest that the association between sex work and HIV acquisition is strong among women who inject drugs, with no association among men who inject drugs, with a much higher proportion of women who inject drugs reporting recent sex work (27% vs 5% in men who inject drugs). This underscores the added importance of ensuring that health and harm reduction services for PWID are equipped to support the needs of women engaged in sex work, including such things as STI testing and reproductive health services.^57^

A key strength of our analysis was the incorporation of both published and unpublished estimates. By including unpublished estimates, we increased statistical power and reduced the risk of publication bias.^58^ We found that the pooled associations were sometimes higher in published studies than in unpublished data, with the greatest discrepancy observed for the association between recent sex work and HCV acquisition. Including these additional unpublished data has likely made our review more representative, enhancing the generalisability of the findings by allowing a wider range of study settings to be included.

Our study has limitations, some of which reflects the underlying data available for inclusion. First, most estimates are from HICs, meaning our findings may be less generalisable to PWID located in LMICs. Second, there was variation across studies in how sex work and male-with-male sex were measured. For example, in four studies, lifetime male-with-male sexual activity was defined based on sexual identity rather than reported behaviour, which could have misclassified exposure, for example by excluding men who have sex with men that identify as heterosexual. Further, the sexual behaviour exposures were based on self-report and responses to these questions may vary across different cultures and could be under-reported in some settings due to behaviours being socially stigmatised. This misclassification is likely to have been largely non-differential with respect to the outcome, thereby biasing estimates toward the null. Third, we included three estimates of same-sex sexual activity in our analyses among participants who were not exclusively men (73%, 83% and 96% of samples were men), one in our pooled analysis of recent exposure and two in our pooled analysis of lifetime exposure. These studies were excluded in our sensitivity analyses, and the results were largely unchanged. Fourth, because our main analyses were crude, we cannot rule out confounding. We opted to prioritise crude estimates due, in part, to the premise that some of the factors included in adjustment (e.g. injecting practices, sexual behaviours, housing instability) may act as mediators rather than confounders, potentially attenuating the effect. In reality, the relationship between these variables is complex. For example, housing instability could also act as a confounder. Thus, this distinction cannot be reliably disentangled in a systematic review relying on estimates with adjustment sets pre-determined by the original study authors. However, adjusted analyses generally produced similar pooled effects. Fifth, there was moderate to high heterogeneity in most of our pooled estimates, limiting their generalisability. While we conducted subgroup analyses across a range of study-level study characteristics, these variables did not explain all the observed heterogeneity, and most subgroup estimates were based on small numbers of studies, resulting in wide, overlapping confidence intervals. Unmeasured sources of heterogeneity likely contributed to differences across studies, particularly variation in how sex work, and male-with-male sex were defined and measured, which was not standardised across included studies and could not be captured by our subgroup categories. Other unmeasured factors may also include the broader social and structural context surrounding sex work or male-with-male sex (e.g. stigma, social acceptance), accessibility of HIV/HCV prevention and harm reduction services, and the epidemic phase. Sixth, transgender PWID were not considered in our stratified analyses of the association between sex work and HIV/HCV incidence, as there was insufficient data to calculate meaningful estimates. Seventh, high baseline risk among PWID may attenuate relative measures of association. As baseline incidence rises, these measures compress toward the null even when the absolute effect of an exposure remains constant. Last, although we contacted cohorts systematically, unpublished estimates did not undergo formal peer review, and we cannot eliminate risk of selection bias as cohorts with prior published data were more likely to be identified for contact, and responding cohorts may systematically differ from non-responding cohorts in ways that could bias the pooled estimate in either direction.

In this global systematic review and meta-analysis among PWID, we found recent sex work was associated with increased HIV risk, particularly among women, and, to a lesser extent, with HCV risk, while male-with-male sexual activity was associated with higher HIV risk among male PWID, but not HCV risk. These findings highlight the need for integrated prevention and treatment services that address both injection-related and sexual risks. Rather than siloed approaches, services should combine access to harm reduction measures (i.e. sterile injecting equipment and OAT, HIV and HCV testing), with sexual health care including provision of pre-exposure prophylaxis and condoms, STI testing and treatment, and reproductive health services. Further research is needed on the intersectionality among PWID and the factors associated with elevated risk among PWID belonging to more than one population, particularly in LMICs where evidence remains limited. This research could help in the development of tailored interventions to mitigate for the increased risks that these PWID experience.

## Contributors

JD, JS, PV and AAA designed the study. JS and PV wrote the study protocol. JD conducted statistical analyses. JD, JS, BJN and AAA screened the articles and extracted data from the studies. AT, BK and LB accessed and verified the underlying data. JD, JW and PV contacted authors to request unpublished data. DA, MA, IA, KB, AB, JB, KDB, DDJ, PD, KD, CH, AH, JRH, KH, MH, VH, VJ, AK, LM, SM, HM, MDM, KP, NEP, LP, MP, KR, LRS, SR, RSD, AS, SAS, SSS, VS, DVS, GZ provided access to unpublished data. JD drafted the first version of the manuscript. JS, PV, AAA provided critical feedback on the first version of the manuscript. All authors read and approved the final manuscript.

## Data sharing statement

Data used in this study are available from the corresponding author upon reasonable request.

## Declaration of interests

KP declares support from the National Institutes of Health (NIH), New Mexico Department of Health, and New Mexico's State Opioid Response (SOR). VS's declares that their institution has received funding from Roche, AstraZeneca, and Gilead Sciences. KDB and JB declares support from AbbVie and Gilead Sciences. PD declares that their institution has received support from Navamedic. VS declares support from Roche, AstraZeneka, and Gilead Sciences, and is an unpaid member of the Scientific Committee of the European Union Drugs Agency. SM declares support from Abbott Diagnostics. All other authors have no competing interests to declare.
