# Supplementary material for: Sex work or male-with-male sexual activity and the risks of HIV and hepatitis C acquisition among people who inject drugs: a global systematic review and meta-analysis
Source: eClinicalMedicine. 2026 Oct 14;101:104268. doi: 10.1016/j.eclinm.2026.104268 (PMC13694819; doi:10.1016/j.eclinm.2026.104268)
Supplement: Supplementary file 1 [file mmc1.pdf]

Supplementary material

Supplement to: Dawe J, Trickey A, J, Walker J, et al. Sex work or male-with-male sexual activity and the risks of HIV and hepatitis C acquisition among people who inject drugs: a global systematic review and meta-analysis

Table of Contents

Appendix 1. PRISMA checklist 3

Appendix 2. Study protocol 6

Appendix 3. Search strategy 11

Appendix 4. Data extraction 14

Appendix 5. Frequencies of extracted associations 15

Appendix 6. Study characteristics 16

Appendix 7. Modified Newcastle-Ottawa scale 35

Appendix 8. Details of studies excluded for duplicate data 36

Appendix 9. Meta analyses of recent exposure to sex work or male-with-male sexual activity and HIV or HCV acquisition 38

Appendix 10. Meta analyses of lifetime exposure to sex work or male-with-male sexual activity and HIV or HCV acquisition 46

Appendix 11. Subgroup analyses of recent exposure to sex work or male-with-male sexual activity and HIV or HCV acquisition 54

Appendix 12. Subgroup analyses of recent exposure to male-with-male sex and HIV or HCV acquisition risk among male PWID 67

Appendix 13. Sensitivity analyses of association between male-with-male sex and risk of HCV acquisition, excluding studies where less than 100% of sample are male PWID 77

Appendix 14. Publication asymmetry 78

Appendix 15. Study quality of published studies for the effect of sex work or male-with-male sexual exposure on HIV and HCV acquisition risk 80

Appendix 16. Study quality of unpublished studies for the effect of sex work or male-with-male sexual exposure on HIV and HCV acquisition risk 82

References 84

Appendix 1. PRISMA checklist

Section and Topic	Item #	Checklist item	Location where item is reported
**TITLE**			
Title	1	Identify the report as a systematic review.	Page 1
**ABSTRACT**			
Abstract	2	See the PRISMA 2020 for Abstracts checklist.	Page 3
**INTRODUCTION**			
Rationale	3	Describe the rationale for the review in the context of existing knowledge.	Page 7
Objectives	4	Provide an explicit statement of the objective(s) or question(s) the review addresses.	Page 8
**METHODS**			
Eligibility criteria	5	Specify the inclusion and exclusion criteria for the review and how studies were grouped for the syntheses.	Page 8
Information sources	6	Specify all databases, registers, websites, organisations, reference lists and other sources searched or consulted to identify studies. Specify the date when each source was last searched or consulted.	Pages 8-9
Search strategy	7	Present the full search strategies for all databases, registers and websites, including any filters and limits used.	Supplementary material
Selection process	8	Specify the methods used to decide whether a study met the inclusion criteria of the review, including how many reviewers screened each record and each report retrieved, whether they worked independently, and if applicable, details of automation tools used in the process.	Page 8–9
Data collection process	9	Specify the methods used to collect data from reports, including how many reviewers collected data from each report, whether they worked independently, any processes for obtaining or confirming data from study investigators, and if applicable, details of automation tools used in the process.	Page 8–9
Data items	10a	List and define all outcomes for which data were sought. Specify whether all results that were compatible with each outcome domain in each study were sought (e.g. for all measures, time points, analyses), and if not, the methods used to decide which results to collect.	Pages 8–9
	10b	List and define all other variables for which data were sought (e.g. participant and intervention characteristics, funding sources). Describe any assumptions made about any missing or unclear information.	Supplementary material
Study quality assessment	11	Specify the methods used to assess study quality in the included studies, including details of the tool(s) used, how many reviewers assessed each study and whether they worked independently, and if applicable, details of automation tools used in the process.	Page 10 and supplementary material
Effect measures	12	Specify for each outcome the effect measure(s) (e.g. risk ratio, mean difference) used in the synthesis or presentation of results.	Page 10
Synthesis methods	13a	Describe the processes used to decide which studies were eligible for each synthesis (e.g. tabulating the study intervention characteristics and comparing against the planned groups for each synthesis (item #5)).	Pages 10–11
	13b	Describe any methods required to prepare the data for presentation or synthesis, such as handling of missing summary statistics, or data conversions.	Pages 10–11

Section and Topic	Item #	Checklist item	Location where item is reported
	13c	Describe any methods used to tabulate or visually display results of individual studies and syntheses.	Pages 10–11
	13d	Describe any methods used to synthesize results and provide a rationale for the choice(s). If meta-analysis was performed, describe the model(s), method(s) to identify the presence and extent of statistical heterogeneity, and software package(s) used.	Pages 10–11
	13e	Describe any methods used to explore possible causes of heterogeneity among study results (e.g. subgroup analysis, meta-regression).	Pages 10–11
	13f	Describe any sensitivity analyses conducted to assess robustness of the synthesized results.	Pages 10–11
Reporting bias assessment	14	Describe any methods used to assess study quality due to missing results in a synthesis (arising from reporting biases).	Pages 10–11
Certainty assessment	15	Describe any methods used to assess certainty (or confidence) in the body of evidence for an outcome.	Pages 10–11
**RESULTS**			
Study selection	16a	Describe the results of the search and selection process, from the number of records identified in the search to the number of studies included in the review, ideally using a flow diagram.	Page 11
	16b	Cite studies that might appear to meet the inclusion criteria, but which were excluded, and explain why they were excluded.	Page 11
Study characteristics	17	Cite each included study and present its characteristics.	Supplementary material
Study quality in studies	18	Present assessments of study quality for each included study.	Supplementary material
Results of individual studies	19	For all outcomes, present, for each study: (a) summary statistics for each group (where appropriate) and (b) an effect estimate and its precision (e.g. confidence/credible interval), ideally using structured tables or plots.	Supplementary material
Results of syntheses	20a	For each synthesis, briefly summarise the characteristics and study quality among contributing studies.	Supplementary material
	20b	Present results of all statistical syntheses conducted. If meta-analysis was done, present for each the summary estimate and its precision (e.g. confidence/credible interval) and measures of statistical heterogeneity. If comparing groups, describe the direction of the effect.	Pages 11–13 and supplementary material
	20c	Present results of all investigations of possible causes of heterogeneity among study results.	Pages 11–13 and supplementary material
	20d	Present results of all sensitivity analyses conducted to assess the robustness of the synthesized results.	Pages 11–13 and supplementary material

Section and Topic	Item #	Checklist item	Location where item is reported
Reporting biases	21	Present assessments of study quality due to missing results (arising from reporting biases) for each synthesis assessed.	Pages 11–13 and supplementary material
Certainty of evidence	22	Present assessments of certainty (or confidence) in the body of evidence for each outcome assessed.	Pages 11–13 and supplementary material
**DISCUSSION**			
Discussion	23a	Provide a general interpretation of the results in the context of other evidence.	Pages 14–16
	23b	Discuss any limitations of the evidence included in the review.	Pages 15
	23c	Discuss any limitations of the review processes used.	Pages 15
	23d	Discuss implications of the results for practice, policy, and future research.	Pages 14–16
**OTHER INFORMATION**			
Registration and protocol	24a	Provide registration information for the review, including register name and registration number, or state that the review was not registered.	Supplementary material
	24b	Indicate where the review protocol can be accessed, or state that a protocol was not prepared.	Supplementary material
	24c	Describe and explain any amendments to information provided at registration or in the protocol.	NA
Support	25	Describe sources of financial or non-financial support for the review, and the role of the funders or sponsors in the review.	End of article
Competing interests	26	Declare any competing interests of review authors.	End of article
Availability of data, code and other materials	27	Report which of the following are publicly available and where they can be found: template data collection forms; data extracted from included studies; data used for all analyses; analytic code; any other materials used in the review.	End of article

Appendix 2. Study protocol

This protocol has been registered with PROSPERO (CRD42020156262).

Review question

Our overall aim is to summarise hepatitis C virus (HCV) and HIV incidence among people who inject drugs (PWID) and to characterise geographic, temporal and individual differences that need to be understood to better orient prevention efforts.

Our specific aims (SA) are to:

- SA1: synthesise estimates of HIV and HCV incidence among PWID;
- SA2: explore differences in HIV and HCV incidence, with a focus on calendar time and geographic location;
- SA3: compare risks of HIV and HCV acquisition across subsets of PWID defined by individual characteristics, with a focus on age, gender, injection frequency, men who have sex with men (MSM) and involvement in sex work.

Searches

An existing database of search records created by our group, which compiled studies reporting HIV and HCV incidence among PWID published between January 1, 2000 and September 14, 2020, will be used as the basis for this systematic review.

The original database included all records up until June 13, 2017 and was recently updated to September 12, 2020.

The database has been used for two systematic reviews documenting the associations between HIV and HCV acquisition risk, and (i) incarceration and (ii) homelessness or unstable housing among PWID.

Without language restrictions, we have searched MEDLINE, Embase, and PsycINFO using terms related to HIV infection or transmission, HCV infection or transmission, injecting drug use, and study designs that could be used to evaluate HIV or HCV incidence.

No other restrictions were applied to the search.

The search strategy used in each of the three databases has been developed in consultation with a medical librarian and has been published (Stone et al Lancet Infect Dis 2018).

The reference lists of systematic reviews were also hand searched to retrieve any other potentially relevant papers or reports.

Types of study to be included

All study designs that have been used to measure HIV and HCV incidence in the past will be included, namely prospective and retrospective cohort studies, cross-sectional studies and randomised controlled studies (control arm).

Condition or domain being studied

Incident HIV and HCV infection.

Participants/population

People who have a history of injecting drug use, either recently (within the past year) or previously.

Intervention(s), exposure(s)

Individual-level characteristics (SA3), with a focus on young age, female gender, high injection frequency, being an MSM and involvement in sex work. Some variables will have been operationalised differently across studies. Therefore, we will use broad definitions when extracting data to allow more estimates to be pooled, and refine our criteria at the analysis stage. For example, we expect that some studies will have used different groupings when presenting age-stratified incidence data. We will therefore define "young age" as the most commonly reported threshold between 20 and 30 and allow for slight differences in the definition (e.g., if a threshold of ≤25 is retained to define young age, then a study using a threshold of ≤24 will also be included). The impact of using a flexible or strict cut-off will be examined in sensitivity analyses. Both gender and sex will be considered in our review and the impact of using the two constructs will be examined in sensitivity analyses. We plan to operationalise high injecting frequency as daily injecting or more, sex work as exchanging sexual services for money, drugs, or other material goods and MSM as reporting sex with another man or identifying as homosexual, gay or bisexual.

Comparator(s)/control

Comparisons: these will be defined according to the strata listed in the exposure section.

Context

In addition to published records, unpublished data will be obtained by contacting authors of published studies to request updated data if incidence estimates are old (e.g., <2010) and/or additional data relevant to this review (e.g., stratification by age if not published yet).

We will also request data from known investigators of ongoing research studies examining HIV/HCV incidence among PWID for which data have not yet been published.

Exclusion:

• Studies that did not report original results; 5

• Incidence estimated among incarcerated PWID, as infection rates and circumstances surrounding transmission are unlikely to be generalizable to the broader PWID population;

• Incidence estimated by self-reported infection status;

• Incidence estimates based on a sample size or total follow-up that are too small, defined here as <10 people or <10 person-years, respectively;

• Incidence estimated though methods other than those specified above (e.g., those relating age or duration of injection with prevalence, or mathematical modelling methods).

Main outcome(s)

Incident HIV and HCV infection. All analyses will be conducted separately for each outcome. We will include studies that measured new HIV/HCV infections by repeat testing of people at risk – the "gold standard method" for measuring incidence, and by tests of recent infection (e.g., based on markers of antibody avidity, detection of HCV RNA-positive among anti-HCV-negative participants). Only primary HCV infection will be considered. To be eligible, all reports must explicitly state that a diagnostic test - whether laboratory or point of care - was used to ascertain HIV and HCV infection status.

Measures of effect

Measure of association between individual-level characteristics (SA3) and HIV and HCV incidence will include incidence rate ratios, hazard ratios, risk ratios or alternatively- if none of these three are available or can be calculated using available data - odds ratios.

Additional outcome(s)

None

Data extraction (selection and coding)

Records obtained via the search strategy were imported into EndNote with the removal of duplicates. Records documenting HIV and HCV incidence have already been selected as part of the previous two reviews as follows: we initially screened titles and abstracts of each record against the inclusion criteria and kept those deemed potentially eligible. These then underwent full-text review. Reasons for exclusion were documented. Each step (i.e., title/abstract screening and full-text review) was done by one author, with 10% of records also screened by a second reviewer. If inconsistences between the lists of accepted records arose, all remaining references were double screened. Any ensuing discord that could not be resolved by discussion was adjudicated by a third author.

For the purposes of this review, a rescreening of eligible records will be done, using the same strategy, to exclude studies that did not distinguish between primary HCV and HCV reinfection.

Eligible records will next be extracted using Microsoft Excel into a standardized spreadsheet. This step will be done by the one author and will be checked in full for accuracy by a second author.

Data items:

- Study characteristics (e.g., lead author, journal name, year of publication, start/end dates, design, city/country, sampling strategy, recruitment venue, urbanicity, inclusion/exclusion criteria, attrition);

- Descriptive characteristics of study sample at baseline (e.g., age, proportion young, proportion female, duration of injecting, proportion who report sex work, being an MSM, high injection frequency, on opioid agonist treatment, homeless/unstably housed, incarcerated, type of drug injected);

- HIV/HCV incidence rate and characteristics (e.g., method of incidence estimation, testing frequency, number of people/observations at risk, number of incident cases, person-years at risk - items extracted overall and stratified by (i) calendar year, (ii) geographic settings, (iii) individual-level characteristics;

- Measures of association between individual-level characteristics and HIV/HCV incidence;

- Background HIV/HCV prevalence.

Study quality (quality) assessment

As in previous reviews conducted by our group (Stone et al Lancet Infect Dis 2018), we will use the Newcastle-Ottawa-Scale (NOS) to assess the quality of studies.

Study quality assessment will be carried out by two researchers independently and disagreements will be resolved by a third.

Strategy for data synthesis

We anticipate heterogeneity across studies, particularly with respect to HIV and HCV incidence. Therefore, although pooled estimates will be presented, the emphasis will be on quantifying and interpreting this heterogeneity.

We will use random-effects meta-analyses to pool incidence rates and effect measures comparing rates by individual-level characteristics.

Estimates will be transformed to the natural logarithmic scale and back-transformed for reporting.

Heterogeneity will be tested using the χ^2 test and quantified using the I^2-statistic.

Analysis of subgroups or subsets

We will conduct subgroup analyses and random-effects meta-regression to explore sources of heterogeneity (i.e., effect modification), focusing primarily on calendar year and geographic setting (SA2). Calendar year of data collection will be defined 6 as the midpoint between start and end dates of data collection. Partially aggregated data by calendar year will be used in meta-regression analyses, when available, using specific methods to account for clustering. Geographic setting will be defined using regional and socio-economic classifications by the World Bank. The other possible sources of heterogeneity that we will consider are:

• Study characteristics (e.g., sampling strategy, recruitment venue, method of incidence estimation);

• Participant characteristics (e.g., duration of injection, proportion receiving OAT, proportion incarcerated, proportion homeless, type of drug injected);

• Country-level coverage of harm-reduction programs, as measured in previous studies, and indicators of health, development, and inequality (e.g., Human Development Index, Gender Inequality Index).

Sensitivity analyses will be performed to assess the impact of restricting meta-analyses to:

• Estimates of HIV/HCV incidence post-2010 (if incidence spans a period pre- and post-2010, then the midpoint of data collection will be used to classify a study);

• Estimates of HIV/HCV incidence spanning a shorter period of time (e.g., ≤ 5 years);

• Estimates of HIV/HCV incidence estimated based on repeat testing;

• Studies at low/moderate study quality;

• Studies in which the recruitment occurred in the community;

• Studies with ≥90% recent PWID;

• Studies in which the threshold and/or definition to define individual-level characteristics is homogeneous (e.g., same threshold to classify young age).

Appendix 3. Search strategy

Medline database search strategy to identify relevant studies. Keywords are listed in regular type and medical subject headings (Mesh) terms are in **Bold.**

1. Hepatitis C OR HCV OR **exp hepatitis C/**

2. HIV OR human immunodeficiency virus OR exp **HIV seropositivity/** OR **exp HIV seroprevalence**/ OR **exp HIV infections**/ OR **exp HIV**/

3. 1 OR 2

4. IDU OR IDUs OR IVDU OR IVDUs OR PWID OR PWIDs

5. (substance* or drug*).mp. [mp=title, abstract, original title, name of substance word, subject heading word, floating sub-heading word, keyword heading word, organism supplementary concept word, protocol supplementary concept word, rare disease supplementary concept word, unique identifier, synonyms]

6. ((substance* or drug*) adj3 (inject* or intravenous)).mp. [mp=title, abstract, original title, name of substance word, subject heading word, floating sub-heading word, keyword heading word, organism supplementary concept word, protocol supplementary concept word, rare disease supplementary concept word, unique identifier, synonyms]

7. ((substance* or drug*) adj3 (abuse* or depend* or use* or misus* or addict*)).mp. [mp=title, abstract, original title, name of substance word, subject heading word, floating sub-heading word, keyword heading word, organism supplementary concept word, protocol supplementary concept word, rare disease supplementary concept word, unique identifier, synonyms]

8. exp substance abuse, intravenous/

9. 4 OR 6 OR 7 OR 8

10. Prevalence OR incidence OR epidemiol* OR survey OR rapid assessment OR situation assessment OR situational assessment OR RAR OR cohort OR surveillance OR seroprevalence OR seroincidence OR seroepidemiol* OR seroconv* OR screening OR **exp epidemiologic methods**/ OR **exp epidemiologic studies**/ OR **exp sentinel surveillance**/ OR **exp seroepidemiologic studies**/ OR **exp cohort studies**/ OR **exp cross-sectional studies**/ OR **exp longitudinal studies**/ OR **exp follow-up studies**/ OR **exp prospective studies**/

11. 3 AND 9 AND 10

12. Limit 11: animals/ not (humans/ and animals/)

13. 11 NOT 12

14. **(202009* or 202010* or 202011* or 202012* or 2021* or 2022*). dt, ez, ed.**

15. 13 AND 14.

Embase database search strategy to identify relevant studies. Keywords are listed in regular type and Emtree terms in Bold.

1. Hepatitis C OR HCV OR **exp hepatitis C**/ OR **exp Hepatitis C virus**/

2. HIV OR human immunodeficiency virus OR **exp human immunodeficiency virus**/ OR **exp human immunodeficiency virus infection**/ OR **exp human immunodeficiency virus prevalence**/

3. 1 OR 2

4. IDU OR IDUs OR IVDU OR IVDUs OR PWID OR PWIDs

5. ((substance* or drug*) adj3 (inject* or intravenous)).mp. [mp=title, abstract, original title, name of substance word, subject heading word, floating sub-heading word, keyword heading word, organism supplementary concept word, protocol supplementary concept word, rare disease supplementary concept word, unique identifier, synonyms]

6. ((substance* or drug*) adj3 (abuse* or depend* or use* or misus* or addict*)).mp. [mp=title, abstract, original title, name of substance word, subject heading word, floating sub-heading word, keyword heading word, organism supplementary concept word, protocol supplementary concept word, rare disease supplementary concept word, unique identifier, synonyms]

7. exp intravenous drug abuse/

8. 4 OR 5 OR 6 OR 7

9. Prevalence OR incidence OR epidemiol* OR survey OR rapid assessment OR situation assessment OR situational assessment OR RAR OR cohort OR surveillance OR seroprevalence OR seroincidence OR screening OR **exp seroepidemiology**/ OR **exp seroprevalence**/ OR **exp epidemiology**/ OR **exp prevalence**/ OR **exp epidemiologic data**/ OR **exp incidence**/ OR **exp observational study**/ OR **exp cohort analysis**/

10. 3 AND 8 AND 9

11. Limit 10: animals/ not (humans and animals/)

12. 10 NOT 11

13. **Limit 12 to dc=20200901 – 20221212**

PyscINFO database search strategy to identify relevant studies. Keywords are listed in regular type and Thesaurus terms in **Bold**.

1. Hepatitis C OR HCV OR **exp Hepatitis**/

2. HIV OR human immunodeficiency virus OR **exp HIV**/

3. 1 OR 2

4. IDU OR IDUs OR IVDU OR IVDUs OR PWID OR PWIDs 8

5. ((substance* or drug*) adj3 (inject* or intravenous)).mp. [mp=title, abstract, original title, name of substance word, subject heading word, floating sub-heading word, keyword heading word, organism supplementary concept word, protocol supplementary concept word, rare disease supplementary concept word, unique identifier, synonyms]

6. ((substance* or drug*) adj3 (abuse* or depend* or use* or misus* or addict*)).mp. [mp=title, abstract, original title, name of substance word, subject heading word, floating sub-heading word, keyword heading word, organism supplementary concept word, protocol supplementary concept word, rare disease supplementary concept word, unique identifier, synonyms]

7. exp intravenous drug usage/

8. 4 OR 5 OR 6 OR 7

9. (prevalence or incidence or epidemiol* or survey or rapid assessment or situation assessment or situational assessment or RAR or Cohort or surveillance or seroprevalence or seroincidence or screening).mp. or **exp epidemiology**/ or **exp surveys**/ or **exp cohort analysis**/ or **exp longitudinal studies**/ or **exp follow-up studies**/ or **exp prospective studies**/ [mp=title, abstract, original title, name of substance word, subject heading word, floating sub-heading word, keyword heading word, organism supplementary concept word, protocol supplementary concept word, rare disease supplementary concept word, unique identifier, synonyms]

10. 4 AND 8 AND 9

11. Limit 10: animals/ not (humans/ and animals/)

12. 10 NOT 11

13. Limit 12 to up=20200901 - 20221212

Appendix 4. Data extraction

Data were extracted from included studies using Microsoft Excel. We extracted the following variables:

Study characteristics

- First author
- Year of publication (if published data)
- Journal study was published in
- Name of study cohort
- Study data collection years
- Study city and country
- Study design
- Sampling strategy
- Method of incidence assessment
- Testing frequency
- Mean and median duration of follow-up
- Proportion lost to follow-up and how this was defined
- Whether the association between basic demographics (i.e., age and gender) and loss to follow-up was statistically significant

Participant characteristics

- Baseline distribution of gender or sex
- Mean and median age
- Mean and median duration of injection
- Injecting frequency
- Definition of male-with-male sex or sex work
- Number and proportion recently/ever engaged in male-with-male sex or sex work
- Number and proportion recently/ever received opioid agonist treatment[a]
- Number and proportion recently/ever lived in unstable housing[a]
- Number and proportion recently/ever incarcerated[a]

[a] Recently exposed extracted if available, otherwise lifetime exposure extracted

Outcomes

- Study HIV and/or HCV incident cases
- Study HIV and/or HCV incidence rate
- Total study duration at risk measured in person-years
- Measures of association and 95% CI comparing HIV or HCV acquisition among PWID who engaged in male-with-male sex or sex work compared with those who did not

Appendix 5. Frequencies of extracted associations

	Sex work – males and females		Sex work – males		Sex work – females		Male-with-male sex		Total		
	HIV	HCV	HIV	HCV	HIV	HCV	HIV	HCV	HIV	HCV	Total
Recent											
Crude	20	22	12	14	12	12	13	8	57	56	113
Adjusted	11	12	6	10	7	11	6	6	30	39	69
Lifetime											
Crude	6	5	7	5	3	4	8	12	24	26	50
Adjusted	4	4	3	4	3	3	8	5	18	16	34
Total	41	43	28	33	25	30	35	31	129	137	266

Appendix 6. Study characteristics

Study details

Study	Journal	Cohort	City	Country	Study design	Sampling strategy	Recruitment	Inclusion criteria	Exclusion criteria
Aladashvili (Unpublished)	NA	NA	Multi-city	Georgia	Prospective cohort	Convenience and peer referral	Community-based	PWID aged between 18 and 65 and resided in one of the Georgian cities of Tbilisi, Batumi, and Poti	NR
Antuori (2021) (1)	Hepatology	HepCdetect	Barcelona	Spain	Cross-sectional	Convenience	Community-based	PWID ≥ 18 years old who had injected drugs over the previous 6 months.	NR
Bach (2016) (2)	American Journal of Public Health	ACCESS and VIDUS	Vancouver	Canada	Prospective cohort	Convenience and peer referral	Mix	18 years of age, living within the Greater Vancouver Area, and having a history of illicit drug use	NR
Blouin (2024) (3)	International Journal of Drug Policy	SurvUDI	Eastern Central Canada	Canada	Prospective cohort	Convenience	Community-based	Aged 14 and older, injecting at least once within the past 6 months, speaking French or English and being able to provide informed consent	NR
Blouin and Hankins (Unpublished)	NA	SurvUDI	Eastern Central Canada	Canada	Prospective cohort	Convenience	Community-based	Aged 14 and older, injecting at least once within the past 6 months, speaking French or English and being able to provide informed consent	NR
Booth (2016) (4)	Lancet HIV	NR	Odessa, Donetsk, Nikolayev	Ukraine	RCT	Peer referral	Network	Aged ≥16, injected drugs in past 30 days, willing to be interviewed and tested for HIV, not cognitively impaired, HIV-negative at baseline	Too impaired to consent, HIV-positive at baseline, not meeting other inclusion criteria
Boyd, Prins, Jongen and Van Santen (Unpublished)	NA	Amsterdam Cohort Study	Amsterdam	Netherlands	Prospective cohort	Convenience	Health service	Use hard drugs (heroin, cocaine, amphetamines), informed consent	NR
Bruneau and Zang (Unpublished)	NA	HEPCO	Montreal	Canada	Prospective cohort	Convenience	Community-based	≥18 years, injected drugs in past 6 months	HIV positive at baseline, recruited before 1992 (for this analysis)

Study	Journal	Cohort	City	Country	Study design	Sampling strategy	Recruitment	Inclusion criteria	Exclusion criteria
DeBeck (a) (Unpublished)	NA	ARYS	Vancouver	Canada	Prospective cohort	Convenience	Community-based	Age (14 to 26 years) and use of drugs other than marijuana in the past 30 days	NR
DeBeck (b) (Unpublished)	NA	ARYS and VIDUS	Vancouver	Canada	Prospective cohort	Convenience	Community-based	NR	NR
Dietze, Sacks-Davis and Kathnayake (Unpublished)	NA	SuperMIX	Melbourne	Australia	Prospective cohort	Convenience	Community-based	Injected either heroin and/or amphetamines at least once a month for the 6 months preceding baseline interview, willing to provide detailed contact information, holding a valid Australian universal health care insurance (Medicare) number and currently residing in Melbourne or the Greater Geelong region	NR
Dumchev (Unpublished)	NA	NA	Multi-city	Ukraine	Prospective cohort	Convenience	Community-based	NR	NR
Friedman (2024) (5)	Clinical Infectious Diseases	La Frontera	San Diego, Tijuana	USA, Mexico	Prospective cohort	Convenience	Community-based	≥18 years, injected drugs in past month (confirmed by marks), English/Spanish, resident of SD/TJ	HCV or HIV seropositive at baseline (for respective analyses), missing serology
Geddes (2020) (6)	Clinical Infectious Diseases	INC3 (ACS, ATAHC, BAHSTION, BBAASH, HCVC, HITS-p, HITS-c, HepCo, SuperMIX, UFO)	Amsterdam, Sydney, Boston, Baltimore, Montreal, Melbourne, San Francisco	Multiple	Prospective cohort	Convenience	Mix	NR	No injection history, no follow-up, sex not recorded, no OAT exposure data
Hagan (2004) (7)	Epidemiology	RAVEN	Seattle	USA	Prospective cohort	Probability	Mix	Eligibility criteria included having injected an illicit drug at least once in the previous year, age 14 or older, and English or Spanish speaking.	NR
Hahn (2002) (8)	Journal of Infectious Diseases	NR	San Francisco	USA	Prospective cohort	Convenience	Community-based	<30 years old, injected in past month, HCV-negative at baseline	HCV-positive at baseline, no follow-up

Study	Journal	Cohort	City	Country	Study design	Sampling strategy	Recruitment	Inclusion criteria	Exclusion criteria
Havens and Young (Unpublished)	NA	SNAP	Kentucky	USA	Prospective cohort	Respondent-driven	Community-based	18+ years of age, residing in an Appalachian Kentucky county, and having used at least one of the following to get high in the past 30 days: prescription opioids, heroin, crack/cocaine or methamphetamine	NR
Hill (2021) (9)	Open Form Infectious Diseases	NR	San Diego	USA	Prospective cohort	Convenience	HIV clinic	PWH, treated with DAA, achieved SVR, ≥1 post-SVR HCV RNA test	No post-SVR HCV RNA, or genotype change before SVR
Jain (2020) (10)	AIDS and Behavior	Proyecto El Cuete IV	Tijuana	Mexico	Prospective cohort	Targeted	Community-based	≥18 years, injected in past month, visual evidence of injection, English/Spanish, consent, plan to stay ≥24 months, no current intervention study, HIV-negative at baseline, ≥1 follow-up	HIV-positive at baseline, no follow-up
Kozlov (2006) (11)	AIDS	NR	St Petersburg	Russia	Prospective cohort	Mix	Community-based	HIV-negative, injected ≥3 times/week in past month or ≥3 times in past 3 months used after another	HIV-positive at baseline
Kozlov (2016) (12)	Medicine	NR	St Petersburg	Russia	Prospective cohort	Snowball	Clinic-based	HIV-negative, injected/shared paraphernalia in past 6 months, age ≥18	HIV-positive at baseline
Kral (2001) (13)	The Lancet	Urban Health Study	San Francisco	USA	Prospective cohort	Targeted	Community-based	Recent injection (past 30 days), visible signs of injection, HIV-negative at baseline	HIV-positive at baseline, >3 years between last negative and first positive, missing sex
Kulchynska (2026) (14)	AIDS	Ukraine IBBS	Multi-city	Ukraine	Cross-sectional	Respondent-driven	Community-based	NR	NR
Kurth and Walker (Unpublished)	NA	NR	Nairobi	Kenya	Prospective cohort	Respondent-driven	Community-based	≥18 years old, living in Nairobi or Coastal Kenya (Coast Province including Kwale, Mombasa, Kilifi counties), lifetime history of injecting drugs, report using any illicit, non-prescribed drugs by any	NR

Study	Journal	Cohort	City	Country	Study design	Sampling strategy	Recruitment	Inclusion criteria	Exclusion criteria
								route of administration in past year, able and willing to provide informed consent	
Lucas (2015) (15)	AIDS	NR	15 cities	India	Cross-sectional	Respondent-driven	Community-based	Age ≥18, injected in prior 2 years, verbal consent, valid coupon	Seeds excluded from analysis
Maher (Unpublished)	NA	ANSPS	Multi-city	Australia	Cross-sectional	Convenience	NSPs	Needle Syringe Program client	NR
Martin (2014) (16)	PLOS One	Bangkok Tenofovir Study	Bangkok	Thailand	RCT	Convenience	Community-based	HIV-negative, age 20–60, injected drugs in previous year, consent	HIV-positive, age <20 or >60, not injected in previous year, did not consent
Martinello (2023) (17)	AIDS	REACT	Multi-city	Multiple	Prospective cohort	Clinical trial	Community-based	Recent HCV infection (<12 months), treated with sofosbuvir-velpatasvir, end of treatment response, ≥1 posttreatment follow-up	No posttreatment follow-up, virological failure without subsequent response
Mehta (2006) (18)	Journal of Acquired Immune Deficiency Syndrome	ALIVE	Baltimore	USA	Prospective cohort	Targeted	Community-based	Age ≥18, nonmedical injection drug use in prior 11 years, HIV-negative, AIDS-free at entry	HIV-positive at baseline, AIDS at entry
Mirzazadeh (2018) (19)	AIDS and Behavior	UFO	San Francisco	USA	Prospective cohort	Convenience	Community-based	<30 years, injected in past month, English-speaking, HCV-negative (since 2003), planned to stay 3+ months	HCV-positive (since 2003), no baseline blood, no follow-up, no follow-up blood
Mitchell and Clarke (Unpublished)	NA	UAM	Multi-city	UK	Cross-sectional	Convenience	Health and harm reduction services	Current or lifetime injecting drug use	NR
Moles (2020) (20)	Scientific Reports	DRIVE	HaiPhong	Vietnam	Prospective cohort	Respondent-driven	Network	Active PWID (recent skin marks, positive urine test), not on MAT, not planning to move, not sentenced to prison, HIV- or HCV-negative	NR
Morris and Nguyen (Unpublished)	NA	UFO	San Francisco	USA	Prospective cohort	Convenience	Community-based	Less than 30 years of age; self reported use of injection drugs in the past 30 days, ability to provide informed consent, understanding spoken English and,	NR

Study	Journal	Cohort	City	Country	Study design	Sampling strategy	Recruitment	Inclusion criteria	Exclusion criteria
								after 2005, self-reported HCV-negative or unknown status	
Mravcik (Unpublished)	NA	NR	Multi-city	Czech Republic	Prospective cohort	Convenience	Low-threshold treatment program	Injection drug use within the last year, aged over 15 years, informed consent, mental and physical fitness to participate in the study	NR
Palmateer (Unpublished)	NA	NESI	Scotland	UK	Cross-sectional	Convenience	Community-based	Injected drugs on at least one occasion and if it was the first time that they had participated in the current survey year	NR
Platt and Hope (Unpublished)	NA	NR	Multi-city	UK	Cross-sectional	Convenience	Community-based	NR	NR
Read (2024) (21)	Viruses	Kirketon Road Centre	Sydney	Australia	Retrospective cohort	Convenience	Low-threshold treatment program	Previous DAA treatment, past 6 month IDU	NR
Roy (2007) (22)	International Journal of STD & AIDS	SurvUDI	Eastern Central Canada	Canada	Prospective cohort	Convenience	Community-based	Injected at least once in past 6 months, participated ≥2 times, provided consent	NR
Roy (2011) (23)	AIDS	SurvUDI	Eastern Central Canada	Canada	Prospective cohort	Convenience	Community-based	Injected at least once in past 6 months, participated ≥2 times, provided consent	NR
Sarna and Ray Saraswati (Unpublished)	NA	AVHI	Delhi	India	Prospective cohort	Convenience, targeted and peer referral	Community-based	Injected at least once in the last 3 months	NR
Scheim (2018) (24)	AIDS and Behavior	VIDUS	Vancouver	Canada	Prospective cohort	Snowball	Community-based	Male, HIV-negative at baseline, injected drugs in past month, ≥18 years, ≥2 follow-up visits, Vancouver resident	HIV-positive at baseline, <2 follow-up visits
Skaathun (2024) (25)	The Lancet Regional Health	La Frontera	Multi-city	USA and Mexico	Prospective cohort	Convenience	Community-based	Age ≥18, injected drugs in last month, lived in SD or Tijuana, consent	NR
Solomon and Mehta (Unpublished)	NA	NR	Multi-city	India	Prospective cohort	Respondent-driven	Community-based	18 years or older, reported injecting drug use in the prior 2 years, provided verbal informed consent, and presented	NR

Study	Journal	Cohort	City	Country	Study design	Sampling strategy	Recruitment	Inclusion criteria	Exclusion criteria
								a valid recruitment coupon	
Solomon, Mehta and Clipman (Unpublished)	NA	The Spatial Network Study	New Delhi	India	Prospective cohort	Respondent-driven	Community-based	≥18 years of age, provide written informed consent, recruited to participate in the study via a network referral card, match description provided by their recruiter, and not identified as a duplicate participant by biometric (fingerprint) match	NR
Spada (2018) (26)	Journal of Urban Health	NR	Country-wide	Italy	Prospective cohort	Consecutive sampling	Urban rehabilitation centers	Attending DDRC, anti-HCV negative at last screening, any age, consent	Anti-HCV positive at baseline
Spittal (2012) (27)	BMC Public Health	Cedar Project	Vancouver, Prince George	Canada	Prospective cohort	Convenience	Community-based	Aboriginal, age 14–30, used illicit drugs (not marijuana) in past month, HCV-negative, ≥1 follow-up	HCV-positive at baseline, no follow-up
Strathdee (2001) (28)	Arch Intern Med	ALIVE	Baltimore	USA	Prospective cohort	Convenience	Community-based	Age ≥18, history of injection drug use in prior 10 years, AIDS-free at entry, HIV-negative	HIV-positive at baseline, AIDS at entry, >3 years between last negative and first positive test
Strathdee, Abramovitz and Artamonova (Unpublished)	NA	La Frontera	Tijuana	Mexico	Prospective cohort	Convenience	Community-based	≥18 years or older, report injecting drugs within the last month (as evidenced through injection stigmata), and report living in San Diego County or Tijuana	NR
Suntharasamai (2009) (29)	Addiction	AIDSVAX B/E HIV	Bangkok	Thailand	RCT	Convenience	Drug-treatment clinics	HIV-negative, IDU, attending BMA drug-treatment clinics, consent	HIV-positive at baseline, no follow-up
Sypsa (2017) (30)	The Journal of Infectious Diseases	ARISTOTLE	Athens	Greece	Prospective cohort	Respondent-driven	Community-based	RDS coupon, injected drugs in past 12 months, ≥18 years, Athens resident	NA
Sypsa and Roussos (a) (Unpublished)	NA	ARISTOTLE	Athens	Greece	Prospective cohort	Respondent-driven	Community-based	18 years or older who had injected drugs in the past 12 months and resided in the Athens metropolitan area	NR

Study	Journal	Cohort	City	Country	Study design	Sampling strategy	Recruitment	Inclusion criteria	Exclusion criteria
Sypsa and Roussos (b) (Unpublished)	NA	ALEXANDROS	Thessaloniki	Greece	Prospective cohort	Respondent-driven	Community-based	Aged 18 years or older, they reported injecting drugs without a prescription in the past, lived in Thessaloniki metropolitan area, had not previously participated in the current RDS round, and presented a valid RDS coupon.	NR
Todd (Unpublished)	NA	NR	Kabul	Afghanistan	Prospective cohort	Time-location	Community-based	Aged≥18 years; recently injecting drugs (within the prior 30 days); currently residing in Kabul; Dari or Pashto speakers (Dari and Pashto are the major official languages of Afghanistan); and able to provide informed consent	NR
Trickey and Des Jarlais (Unpublished)	NA	DRIVE	Haiphong	Vietnam	Prospective cohort	Respondent-driven	Community-based	Aged 18 years or above, currently injecting, defined by self-report of drug injection, confirmed by the detection of heroin in urine and presence of recent skin marks	NR
Vallejo (2015) (31)	European Journal of Epidemiology	Heroin-Itínere	Barcelona, Madrid, Seville	Spain	Prospective cohort	Targeted street sampling and chain-referral	Community-based	≤30 years, heroin use ≥12 days in past 12 months, ≥1 day in past 3 months, injected in past 12 months	NA
Yin (2006) (32)	Chinese Medical Journal	Sichuan	Xichang	China	Prospective cohort	Convenience	Community-based	HIV-seronegative PWID	NR
Zhang (2007) (33)	International Journal of Infectious Diseases	HPTN 033	Xinjiang	China	Prospective cohort	Targeted	Community-based	HIV-negative, injected drugs ≥3 times/week in past month or ≥3 times in past 3 months used after another, willing for 12 months, able to consent and provide locator info, ≥16 years old	Psychiatric disorder, unsafe to participate, unable to consent, or would interfere with study

Participant details

Study	Median age (years)	Mean age (years)	Percentage lifetime incarcerated	Percent recently homeless	Percent recently on OAT	Median/mean years of injecting
Aladashvili (Unpublished)	HIV: 27; HCV: 27	NR	HIV: 19%; HCV: 11%	NR	NR	HIV: 6 years; HCV: 6 years
Antuori (2021) (1)	NR	NR	66%	15%	NR	NR
Bach (2016) (2)	29	NR	NR	28%	12%	NR
Blouin (2024) (3)	NR	37	11%	40%	NR	NR
Blouin and Hankins (Unpublished)	HIV SW: 35; HIV MSM: 36; HCV SW: 31; HCV MSM: 33	NR	HIV SW: 12%; HIV MSM: 14%; HCV SW: 10%; HCV MSM: 13%	HIV SW: 44%; HIV MSM: 47%; HCV SW: 43%; HCV MSM: 46%	HIV SW: 17%; HIV MSM: 16%; HCV SW: 14%; HCV MSM: 13%	HIV SW: 10 years; HIV MSM: 11 years; HCV SW: 5 years; HCV MSM: 7 years
Booth (2016) (4)	NR	32	NR	NR	NR	12 years
Boyd, Prins, Jongen and Van Santen (Unpublished)	HIV SW: 30; HIV MSM: 31; HCV SW: 29; HCV MSM: 29	NR	NR	NR	HIV SW: 23%; HIV MSM: 24%; HCV SW: 23%; HCV MSM: 23%	NR
Bruneau and Zang (Unpublished)	NR	HIV SW: 34; HIV MSM: 35; HCV SW: 35; HCV MSM: 36	HIV SW: 49%; HIV MSM: 49%; HCV SW: 14%; HCV MSM: 16%	HIV SW: 38%; HIV MSM: 43%; HCV SW: 42%; HCV MSM: 45%	HIV SW: 6%; HIV MSM: 6%; HCV SW: 17%; HCV MSM: 15%	HIV SW: 10 years; HIV MSM: 11 years; HCV SW: 9 years; HCV MSM: 10 years
DeBeck (a) (Unpublished)	NR	NR	NR	NR	NR	NR
DeBeck (b) (Unpublished)	HIV: 33 HCV: 25	HIV: 35 HCV: 27	HIV: 20%: HCV: 20%	HIV: 42%; HCV: 50%	HIV: 40%; HCV: 28%	NR
Dietze, Sacks-Davis and Kathnayake (Unpublished)	NR	28	43%	28%	64%	12 years
Dumchev (Unpublished)	NR	HIV: 34 HCV: 33	HIV: 4%; HCV: 9%	NR	HIV: 4%; HCV: 5%	HIV: 14 years; HCV: 13 years
Friedman (2024) (5)	43	NR	9%	42%	6%	19 years
Geddes (2020) (6)	26	NR	NR	29%	100%	7 years
Hagan (2004) (7)	NR	NR	NR	NR	46%	NR
Hahn (2002) (8)	22	NR	30%	69%	NR	NR
Havens and Young (Unpublished)	NR	32	56%	NR	NR	10 years
Hill (2021) (9)	52	NR	NR	NR	NR	NR
Jain (2020) (10)	NR	Overall: 37; Females: 35; Males: 39	NR	NR	NR	Females: 12 years; Males 18 years
Kozlov (2006) (11)	24	NR	NR	NR	NR	NR
Kozlov (2016) (12)	30	31	NR	NR	NR	10 years

Study	Median age (years)	Mean age (years)	Percentage lifetime incarcerated	Percent recently homeless	Percent recently on OAT	Median/mean years of injecting
Kral (2001) (13)	Overall: 42; Females: 41; Males: 43	NR	NR	Overall: 30%; Females: 31%; Males: 30%	Overall: 17%; Females: 20%; Males: 16%	Overall: 19 years; Females: 19 years; Males: 19 years
Kulchynska (2026) (14)	NR	NR	34%	NR	11%	NR
Kurth and Walker (Unpublished)	31	NR	35%	22%	1%	2 years
Lucas (2015) (15)	Northeast: 29; North/Central: 30	NR	Overall: 50%; Northeast: 52%; North/Central: 50%	NR	Overall: 12%; Northeast: 8%; North/Central: 19%	Northeast: 7 years; North/Central: 8 years
Maher (Unpublished)	HIV SW: 29; HIV MSM: 33; HCV SW: 28; HCV MSM: 31	NR	HIV SW: 17%; HIV MSM: 16%; HCV SW: 14%; HCV MSM: 10%	NR	HIV SW: 45%; HIV MSM: 33%; HCV SW: 24%; HCV MSM: 21%	HIV SW: 11 years; HIV MSM: 12 years; HCV SW: 7 years; HCV MSM: 8 years
Martin (2014) (16)	31	NR	23%	NR	NR	NR
Martinello (2023) (17)	NR	NR	12%	NR	NR	NR
Mehta (2006) (18)	35	NR	NR	NR	NR	14 years
Mirzazadeh (2018) (19)	NR	NR	81%	69%	19%	NR
Mitchell and Clarke (Unpublished)	SW: 36; MSM: 36	NR	SW: 60%; MSM: 66%	SW: 41%; MSM: 41%	NR	SW: 11 years; MSM: 12 years
Moles (2020) (20)	36	NR	NR	NR	0	7 years
Morris and Nguyen (Unpublished)	SW: 24; MSM: 24	NR	SW: 72%; MSM: 76%	SW: 47%; MSM: 48%	SW: 15%; MSM: 14%	SW: 5 years; MSM: 5 years
Mravcik (Unpublished)	NR	23	30%	NR	NR	NR
Palmateer (Unpublished)	NR	NR	NR	NR	NR	NR
Platt and Hope (Unpublished)	SW: 31; MSM: 32	NR	SW: 42%; MSM: 41%	SW: 62%; MSM: 67%	SW: 71%; MSM: 71%	SW: 10 years; MSM: 10 years
Read (2024) (21)	45	NR	75%	53%	47%	NR
Roy (2007) (22)	NR	32	NR	NR	NR	10 years
Roy (2011) (23)	NR	NR	NR	NR	NR	NR
Sarna and Ray Saraswati (Unpublished)	30	32	NR	40%	6%	4 years
Scheim (2018) (24)	39	NR	NR	NR	NR	NR
Skaathun (2024) (25)	43	NR	NR	NR	NR	23 years
Solomon and Mehta (Unpublished)	SW: 29; MSM: 29	NR	SW: 7%; MSM: 7%	SW: 7%; MSM: 7%	SW: 22%; MSM: 22%	SW: 6 years; MSM: 6 years
Solomon, Mehta and Clipman (Unpublished)	HIV: 28; HCV: 27	HIV: 31; HCV: 31	HIV: 29%: HCV: 30%	HIV: 20%: HCV: 13%	NR	HIV: 4 years; HCV: 4 years
Spada (2018) (26)	NR	30.8	5%	6%	79%	NR

Study	Median age (years)	Mean age (years)	Percentage lifetime incarcerated	Percent recently homeless	Percent recently on OAT	Median/mean years of injecting
Spittal (2012) (27)	NR	24	NR	NR	NR	NR
Strathdee (2001) (28)	35	NR	NR	NR	NR	14 years
Strathdee, Abramovitz and Artamonova (Unpublished)	NR	HIV SW: 45; HIV MSM: 45; HCV SW: 44; HCV MSM: 44	HIV SW: 8%; HIV MSM: 10%; HCV SW: 9%; HCV MSM: 11%	HIV SW: 62%; HIV MSM: 74%; HCV SW: 55%; HCV MSM: 68%	HIV SW: 4%; HIV MSM: 2%; HCV SW: 1%; HCV MSM: 0%	HIV SW: 22 years; HIV MSM: 24 years; HCV SW: 22 years; HCV MSM: 23 years
Suntharasamai (2009) (29)	26	NR	78%	NR	NR	NR
Sypsa (2017) (30)	NR	36	52%	24%	21%	NR
Sypsa and Roussos (a) (Unpublished)	SW: 36; MSM: 33	SW: 37; MSM: 34	SW: 9%; MSM: 15%	SW: 33%; MSM: 39%	SW: 19%; MSM: 19%	SW: 17 years; MSM: 11 years
Sypsa and Roussos (b) (Unpublished)	SW: 37; MSM: 41	SW: 36; MSM: 38	SW: 11%; MSM: 0%	SW: 18%; MSM: 0%	SW: 24%; MSM: 10%	SW: 16 years; MSM: 14 years
Todd (Unpublished)	NR	SW: 30; MSM: 30	SW: 1%; MSM: 1%	SW: 23%; MSM: 23%	NR	SW: 4 years; MSM: 4 years
Trickey and Des Jarlais (Unpublished)	SW: 39; MSM: 40	SW: 40; MSM: 40	NR	NR	SW: 11%; MSM: 10%	NR
Vallejo (2015) (31)	NR	NR	49%	33%	48%	NR
Yin (2006) (32)	NR	28	NR	NR	NR	NR
Zhang (2007) (33)	29.3	NR	NR	NR	NR	NR

HIV and HCV outcomes

Study	Testing frequency	HIV sample	HCV sample	HIV cases	HIV follow-up	HIV incidence/ 100 PYs	95% CI	HCV cases	HCV follow-up	HCV incidence/ 100 PYs	95% CI
Aladashvili (Unpublished)	6	855	370	21	1846	1.1	NR	103	701	14.7	NR
Antuori (2021) (1)	NA	NR	410	NR	NR	NR	NR	29	NR	NR	NR
Bach (2016) (2)	6	NR	440	NR	NR	NR	NR	142	1868	7.6	NR
Blouin (2024) (3)	6	4074	1413	340	19998	1.7	NR	664	3454	19.2	NR
Blouin and Hankins (Unpublished)	6	2185	926	NR	NR	1.7	NR	664	3454	19.2	NR
Booth (2016) (4)	6	589	NR	158	497.1	31.8	NR	NR	NR	NR	NR
Boyd, Prins, Jongen and Van Santen (Unpublished)	6	730	178	NR	NR	1.5	NR	NR	NR	NR	NR
Bruneau and Zang (Unpublished)	6	2738	449	175	NR	0.3	NR	143	NR	10.6	NR
DeBeck (a) (Unpublished)	6	664	559	10	1612	0.6	NR	103	945	10.9	NR
DeBeck (b) (Unpublished)	6	1954	811	10	1612	0.5	NR	103	945	10.3	NR
Dietze, Sacks-Davis and Kathnayake (Unpublished)	3	NR	134	NR	NR	NR	NR	NR	NR	NR	NR
Dumchev (Unpublished)	6	1966	1237	40	3401.1	1.6	NR	261	1230.8	21.2	NR
Friedman (2024) (5)	6	363	398	10	773.4	1.3	NR	102	699.3	14.3	NR
Geddes (2020) (6)	3-6	NR	701	NR	NR	NR	NR	148	1427	11.2	NR
Hagan (2004) (7)	6-12	NR	484	NR	NR	NR	NR	134	1153	11.6	NR
Hahn (2002) (8)	3	NR	195	NR	NR	NR	NR	48	191	25.1	NR
Havens and Young (Unpublished)	6-12	NR	277	NR	NR	NR	NR	108	1201.8	9	NR
Hill (2021) (9)	NR	NR	118	NR	NR	NR	NR	3	198.5	1.5	NR
Jain (2020) (10)	6	651	NR	43	3440	1.3	NR	NR	NR	NR	NR
Kozlov (2006) (11)	6	417	NR	20	443.5	4.5	NR	NR	NR	NR	NR
Kozlov (2016) (12)	6	380	NR	28	389	7.2	NR	NR	NR	NR	NR
Kral (2001) (13)	6	1192	NR	58	NR	1.2	NR	NR	NR	NR	NR
Kulchynska (2026) (14)	NA	27852	NR	180	NR	NR	NR	NR	NR	NR	NR
Kurth and Walker (Unpublished)	6	979	NR	30	1153.2	2.6	NR	NR	NR	NR	NR
Lucas (2015) (15)	NA	14481	NR	155	NR	1.5	NR	NR	NR	NR	NR
Maher (Unpublished)	12	5650	2333	NR	NR	0.1	NR	NR	NR	7.6	NR

Study	Testing frequency	HIV sample	HCV sample	HIV cases	HIV follow-up	HIV incidence/ 100 PYs	95% CI	HCV cases	HCV follow-up	HCV incidence/ 100 PYs	95% CI
Martin (2014) (16)	1	2413	NR	50	9665	0.5	NR	NR	NR	NR	NR
Martinello (2023) (17)	3	NR	140	NR	NR	NR	NR	17	82	21.1	NR
Mehta (2006) (18)	6	1983	NR	304	14770	2.1	NR	NR	NR	NR	NR
Mirzazadeh (2018) (19)	3	564	NR	10	1073.8	0.9	NR	NR	NR	NR	NR
Mitchell and Clarke (Unpublished)	NA	NR	5600	NR	NR	NR	NR	NR	NR	NR	NR
Moles (2020) (20)	3-12	199	99	0	206	0	NR	18	108	19.4	NR
Morris and Nguyen (Unpublished)	3	NR	1227	NR	NR	NR	NR	245	983	24.9	NR
Mravcik (Unpublished)	3-6	NR	174	NR	NR	NR	NR	13	108	12.1	NR
Palmateer (Unpublished)	NA	NR	1032	NR	NR	NR	NR	20	NR	NR	NR
Platt and Hope (Unpublished)	NA	NR	692	NR	NR	NR	NR	15	NR	NR	NR
Read (2024) (21)	NA	NR	321	NR	NR	NR	NR	36	NR	11	NR
Roy (2007) (22)	6	NR	543	NR	NR	NR	NR	199	733	27.1	NR
Roy (2011) (23)	6	2249	NR	276	10106.6	2.7	NR	NR	NR	NR	NR
Sarna and Ray Saraswati (Unpublished)	NR	3764	NR	NR	NR	9.1	NR	NR	NR	NR	NR
Scheim (2018) (24)	6	1131	NR	102	8075	1.3	NR	NR	NR	NR	NR
Skaathun (2024) (25)	6	563	NR	9	663.4	1.4	NR	NR	NR	NR	NR
Solomon and Mehta (Unpublished)	NA	9448	NR	NR	NR	NR	NR	NR	NR	NR	NR
Solomon, Mehta and Clipman (Unpublished)	6	777	402	NR	NR	21.4	NR	NR	NR	NR	NR
Spada (2018) (26)	6	NR	284	NR	NR	NR	NR	17	291.4	5.8	NR
Spittal (2012) (27)	6	NR	148	NR	NR	NR	NR	45	338.6	11.6	NR
Strathdee (2001) (28)	6	1874	NR	277	8826.5	3.1	NR	NR	NR	NR	NR
Strathdee, Abramovitz and Artamonova (Unpublished)	6	166	150	7	337	2.1	NR	52	245.1	21.2	NR
Suntharasamai (2009) (29)	6	2546	NR	211	6206	3.4	NR	NR	NR	NR	NR
Sypsa (2017) (30)	3	1521	NR	45	1004	4.5	NR	NR	NR	NR	NR
Sypsa and Roussos (a) (Unpublished)	3	NR	1630	NR	NR	NR	NR	9	NR	14.8	NR
Sypsa and Roussos (b) (Unpublished)	NR	1097	NR	27	389	7	NR	NR	NR	NR	NR

Study	Testing frequency	HIV sample	HCV sample	HIV cases	HIV follow-up	HIV incidence/ 100 PYs	95% CI	HCV cases	HCV follow-up	HCV incidence/ 100 PYs	95% CI
Todd (Unpublished)	3-6	NR	479	NR	NR	NR	NR	150	412	36.4	NR
Trickey and Des Jarlais (Unpublished)	NR	266	NR	NR	NR	5.7	NR	NR	NR	NR	NR
Vallejo (2015) (31)	12	NR	77	NR	NR	NR	NR	42	105.4	39.8	NR
Yin (2006) (32)	6	333	NR	12	473.5	2.5	NR	NR	NR	NR	NR
Zhang (2007) (33)	6-12	508	NR	42	475	8.8	NR	NR	NR	NR	NR

HIV and HCV incidence by exposure status among PWID with recent exposure to male-with-male sex or sex work

Study	Exposure	Outcome	Incidence/ 100 PYs among exposed	95% CI	Incidence/ 100 PYs among unexposed	95% CI
Antuori (2021)	Sex work (All)	HCV	-	---	-	---
Bach (2016)	Sex work (All)	HCV	-	---	-	---
Blouin (2024)	Sex work (All)	HIV	NR	---	NR	---
Blouin and Hankins (Unpublished)	Male-with-male sex	HCV	NR	---	NR	---
Blouin and Hankins (Unpublished)	Sex work (All)	HCV	-	---	-	---
Blouin and Hankins (Unpublished)	Sex work (Females)	HCV	-	---	-	---
Blouin and Hankins (Unpublished)	Sex work (Males)	HCV	-	---	-	---
Blouin and Hankins (Unpublished)	Male-with-male sex	HIV	1.7	0.8-2.6	0.8	0.6-1.1
Blouin and Hankins (Unpublished)	Sex work (Females)	HIV	1.3	0.6-2.1	0.88	0.4-1.4
Blouin and Hankins (Unpublished)	Sex work (Males)	HIV	1.9	0.6-3.2	0.9	0.7-1.1
Booth (2016)	Sex work (All)	HIV	NR	---	NR	---
Boyd, Prins, Jongen and Van Santen (Unpublished)	Sex work (All)	HCV	-	---	-	---
Boyd, Prins, Jongen and Van Santen (Unpublished)	Sex work (Females)	HCV	-	---	-	---
Boyd, Prins, Jongen and Van Santen (Unpublished)	Sex work (Males)	HCV	-	---	-	---
Boyd, Prins, Jongen and Van Santen (Unpublished)	Sex work (All)	HIV	2.4	1.6-3.5	1.0	0.8-1.3
Boyd, Prins, Jongen and Van Santen (Unpublished)	Sex work (Females)	HIV	2.4	1.6-3.6	0.9	0.5-1.4
Boyd, Prins, Jongen and Van Santen (Unpublished)	Sex work (Males)	HIV	2.1	0.5-8.4	1.1	0.8-1.4
Bruneau and Zang (Unpublished)	Male-with-male sex	HCV	NR	---	NR	---
Bruneau and Zang (Unpublished)	Sex work (All)	HCV	-	---	-	---
Bruneau and Zang (Unpublished)	Sex work (Females)	HCV	-	---	-	---
Bruneau and Zang (Unpublished)	Sex work (Males)	HCV	-	---	-	---
Bruneau and Zang (Unpublished)	Male-with-male sex	HIV	-	---	-	---
Bruneau and Zang (Unpublished)	Sex work (All)	HIV	NR	---	NR	---
Bruneau and Zang (Unpublished)	Sex work (Females)	HIV	-	---	-	---
Bruneau and Zang (Unpublished)	Sex work (Males)	HIV	NR	---	NR	---
DeBeck (a) (Unpublished)	Sex work (All)	HCV	9.3	5.2-16.9	11.2	9.1-13.7
DeBeck (b) (Unpublished)	Male-with-male sex	HCV	5.9	3.2-11.0	5.8	4.8-7.0
DeBeck (b) (Unpublished)	Sex work (Females)	HCV	14.9	9.5-23.4	6.1	4.6-8.2

Study	Exposure	Outcome	Incidence/ 100 PYs among exposed	95% CI	Incidence/ 100 PYs among unexposed	95% CI
DeBeck (b) (Unpublished)	Sex work (Males)	HCV	8.5	4.3-17.0	5.8	4.8-7.0
DeBeck (b) (Unpublished)	Male-with-male sex	HIV	1.2	0.5-3.3	0.3	0.2-0.4
DeBeck (b) (Unpublished)	Sex work (All)	HIV	1.2	0.7-2.0	0.3	0.2-0.4
DeBeck (b) (Unpublished)	Sex work (Females)	HIV	1.4	0.8-2.3	0.3	0.2-0.6
Dietze, Sacks-Davis and Kathnayake (Unpublished)	Sex work (All)	HCV	-	---	-	---
Dietze, Sacks-Davis and Kathnayake (Unpublished)	Sex work (Females)	HCV	-	---	-	---
Dietze, Sacks-Davis and Kathnayake (Unpublished)	Sex work (Males)	HCV	-	---	-	---
Dumchev (Unpublished)	Sex work (All)	HCV	6.7	0.0-14.3	21.7	19.1-24.4
Dumchev (Unpublished)	Sex work (Females)	HCV	7.1	0.0-15.2	20.9	15.8-26.1
Dumchev (Unpublished)	Sex work (Males)	HCV	20.0	0.0-75.5	22.1	19.0-25.2
Dumchev (Unpublished)	Sex work (All)	HIV	0.7	0.0-105.5	1.3	0.8-1.8
Dumchev (Unpublished)	Sex work (Females)	HIV	0.8	0.0-5.7	2.9	1.7-4.6
Dumchev (Unpublished)	Sex work (Males)	HIV	14.3	0.0-105.5	1.3	0.8-1.8
Hagan (2004)	Sex work (All)	HCV	-	---	-	---
Hahn (2002)	Sex work (All)	HCV	43.0	20.6-77.6	24.4	17.5-32.8
Havens (Unpublished)	Sex work (All)	HCV	6.9	0.1-13.6	9.1	7.3-10.8
Havens (Unpublished)	Sex work (Females)	HCV	7.3	0.0-17.5	9.5	6.9-12.1
Havens (Unpublished)	Sex work (Males)	HCV	6.4	0.0-15.4	8.7	6.3-11.1
Jain (2020)	Sex work (All)	HIV	NR	---	NR	---
Kozlov (2006)	Sex work (All)	HIV	9.7	3.6-21.1	3.7	2.0-6.2
Kozlov (2006)	Sex work (Females)	HIV	10.9	3.5-25.4	1.1	0.1-6.0
Kozlov (2006)	Sex work (Males)	HIV	6.1	0.2-33.8	4.5	2.4-7.7
Kral (2001)	Male-with-male sex	HIV	-	---	-	---
Kral (2001)	Sex work (All)	HIV	NR	---	NR	---
Kral (2001)	Sex work (Females)	HIV	-	---	-	---
Kral (2001)	Sex work (Males)	HIV	NA	---	NA	---
Kulchynska (2026)	Sex work (All)	HIV	NR	---	NR	---
Kurth and Walker (Unpublished)	Sex work (All)	HIV	6.0	1.6-15.4	2.4	1.6-3.5
Kurth and Walker (Unpublished)	Sex work (Females)	HIV	8.5	2.3-21.7	5.3	1.1-15.6

Study	Exposure	Outcome	Incidence/ 100 PYs among exposed	95% CI	Incidence/ 100 PYs among unexposed	95% CI
Kurth and Walker (Unpublished)	Sex work (Males)	HIV	2.6	0.0-24.1	2.3	1.5-3.4
Lucas (2015)	Male-with-male sex	HIV	-	---	-	---
Maher (Unpublished)	Sex work (All)	HCV	9.0	7.0-11.5	6.8	6.3-7.3
Maher (Unpublished)	Sex work (Females)	HCV	8.6	6.3-11.7	6.4	5.6-7.3
Maher (Unpublished)	Sex work (Males)	HCV	9.80	6.3-15.2	7.0	6.4-7.6
Maher (Unpublished)	Sex work (All)	HIV	0.1	0.1-0.4	0.1	0.0-0.1
Maher (Unpublished)	Sex work (Females)	HIV	0.05	0.0-0.3	0.03	0.0-0.1
Maher (Unpublished)	Sex work (Males)	HIV	0.4	0.1-1.4	0.1	0.1-0.1
Martin (2014)	Male-with-male sex	HIV	0.5	0.0-1.7	0.6	0.4-0.8
Mirzazadeh (2018)	Male-with-male sex	HIV	4.0	0.8-11.7	1.0	0.4-2.2
Mirzazadeh (2018)	Sex work (All)	HIV	1.4	0.0-7.8	0.8	0.3-1.6
Mitchell and Clarke (Unpublished)	Male-with-male sex	HCV	NR	---	NR	---
Mitchell and Clarke (Unpublished)	Sex work (All)	HCV	-	---	-	---
Mitchell and Clarke (Unpublished)	Sex work (Females)	HCV	NA	---	NA	---
Mitchell and Clarke (Unpublished)	Sex work (Males)	HCV	-	---	-	---
Moles (2020)	Sex work (All)	HCV	-	---	-	---
Morris and Nguyen (Unpublished)	Sex work (All)	HCV	NR	---	NR	--0.0
Morris and Nguyen (Unpublished)	Sex work (Females)	HCV	NR	---	NR	---
Morris and Nguyen (Unpublished)	Sex work (Males)	HCV	NR	---	NR	---
Palmateer (Unpublished)	Sex work (All)	HCV	-	---	-	---
Platt and Hope (Unpublished)	Male-with-male sex	HCV	NA	---	NA	---
Platt and Hope (Unpublished)	Sex work (All)	HCV	-	---	-	---
Platt and Hope (Unpublished)	Sex work (Females)	HCV	NA	---	NA	---
Platt and Hope (Unpublished)	Sex work (Males)	HCV	-	---	-	---
Roy (2007)	Male-with-male sex	HCV	NR	---	NR	---
Roy (2007)	Sex work (All)	HCV	-	---	-	---
Roy (2011)	Sex work (All)	HIV	NR	---	NR	---
Sarna and Ray Saraswati (Unpublished)	Sex work (Males)	HIV	6.7	2.1-20.6	9.1	8.1-10.2
Skaathun (2024)	Sex work (All)	HIV	1.8	0.0-3.6	1.1	0.1-2.1

Study	Exposure	Outcome	Incidence/ 100 PYs among exposed	95% CI	Incidence/ 100 PYs among unexposed	95% CI
Solomon and Mehta (Unpublished)	Male-with-male sex	HIV	5.8	0.6-10.7	5.0	4.0-5.9
Solomon and Mehta (Unpublished)	Sex work (All)	HIV	2.3	0.0-4.6	5.1	4.2-6.1
Solomon and Mehta (Unpublished)	Sex work (Males)	HIV	2.6	0.0-5.2	5.0	4.1-6.0
Spittal (2012)	Sex work (All)	HCV	-	---	-	---
Strathdee (2001)	Male-with-male sex	HIV	10.4	---	3.0	---
Strathdee (2001)	Sex work (All)	HIV	3.6	2.7-4.5	3.2	2.8-3.7
Strathdee (2001)	Sex work (Females)	HIV	4.7	2.3-8.4	3.3	2.4-4.3
Strathdee (2001)	Sex work (Males)	HIV	3.4	2.5-4.4	3.2	2.7-3.8
Strathdee, Abramovitz and Artamonova (Unpublished)	Male-with-male sex	HCV	6.9	0.0-20.5	22.4	15.0-29.8
Strathdee, Abramovitz and Artamonova (Unpublished)	Sex work (All)	HCV	-	---	19.7	5.1-34.3
Strathdee, Abramovitz and Artamonova (Unpublished)	Sex work (Females)	HCV	19.6	2.4-37.8	22.6	9.2-35.9
Strathdee, Abramovitz and Artamonova (Unpublished)	Sex work (Males)	HCV	19.9	0.0-47.4	21.1	14.0-28.2
Strathdee, Abramovitz and Artamonova (Unpublished)	Male-with-male sex	HIV	4.3	0.0-16.2	1.4	0.0-2.9
Strathdee, Abramovitz and Artamonova (Unpublished)	Sex work (Females)	HIV	5.3	0.0-12.7	4.7	0.0-11.2
Strathdee, Abramovitz and Artamonova (Unpublished)	Sex work (Males)	HIV	7.9	0.0-23.5	0.8	0.0-2.0
Suntharasamai (2009)	Male-with-male sex	HIV	-	---	3.2	2.7-3.6
Sypsa (2017)	Male-with-male sex	HIV	-	---	-	---
Sypsa (2017)	Sex work (Females)	HIV	-	---	-	---
Sypsa and Roussos (a) (Unpublished)	Sex work (All)	HCV	47.5	3.0-759.9	14.8	7.8-27.9
Sypsa and Roussos (b) (Unpublished)	Male-with-male sex	HIV	23.3	3.3-165.7	6.6	4.3-10.0
Sypsa and Roussos (b) (Unpublished)	Sex work (All)	HIV	12.3	3.1-49.0	6.8	4.6-10.0
Todd (Unpublished)	Male-with-male sex	HCV	65.8	33.9-127.5	36.5	31.9-41.6
Todd (Unpublished)	Sex work (Males)	HCV	36.0	11.8-100.9	36.7	32.1-41.9
Trickey and Des Jarlais (Unpublished)	Sex work (Males)	HCV	-	---	4.9	---
Trickey and Des Jarlais (Unpublished)	Sex work (All)	HIV	2.3	0.0-8.6	5.8	0.0-12.1
Vallejo (2015)	Sex work (All)	HCV	93.8	---	48.9	---
Yin (2006)	Sex work (All)	HIV	NR	---	NR	---

HIV and HCV incidence by exposure status among PWID with lifetime exposure to male-with-male sex or sex work

Study	Exposure	Outcome	Incidence/ 100 PYs among exposed	95% CI	Incidence/ 100 PYs among unexposed	95% CI
Aladashvili (Unpublished)	Male-with-male sex	HCV	20.9	6.8-48.7	14.5	11.8-17.6
Aladashvili (Unpublished)	Male-with-male sex	HIV	5.5	1.5-14.0	1.5	1.0-0.6
Aladashvili (Unpublished)	Sex work (Male)	HCV	22.0	4.5-64.3	14.5	11.8-17.7
Aladashvili (Unpublished)	Sex work (Male)	HIV	3.6	0.4-12.8	1.1	0.6-1.7
Boyd, Prins, Jongen and Van Santen (Unpublished)	Male-with-male sex	HCV	NR	---	NR	---
Boyd, Prins, Jongen and Van Santen (Unpublished)	Male-with-male sex	HIV	0.3	0.1-1.0	1.7	1.3-1.0
Bruneau and Zang (Unpublished)	Sex work (All)	HCV	-	---	-	---
Bruneau and Zang (Unpublished)	Sex work (All)	HIV	NR	---	NR	---
Bruneau and Zang (Unpublished)	Sex work (Female)	HCV	-	---	-	---
Bruneau and Zang (Unpublished)	Sex work (Female)	HIV	-	---	-	---
Bruneau and Zang (Unpublished)	Sex work (Male)	HCV	-	---	-	---
Bruneau and Zang (Unpublished)	Sex work (Male)	HIV	NR	---	NR	---
Dietze, Sacks-Davis and Kathnayake (Unpublished)	Male-with-male sex	HCV	9.5	1.3-6.7	15.3	9.2-25.3
Friedman (2024)	Male-with-male sex	HCV	NR	---	NR	---
Geddes (2020)	Male-with-male sex	HCV	NR	---	NR	---
Hill (2021)	Male-with-male sex	HCV	4.6	0.6-16.7	0.6	0.0-3.6
Kozlov (2016)	Sex work (All)	HIV	9.7	1.2-35.2	7.4	4.6-11.3
Kozlov (2016)	Sex work (Male)	HIV	11.1	1.3-40.2	7.7	4.4-12.5
Kurth and Walker (Unpublished)	Sex work (All)	HIV	4.9	1.6-11.4	2.4	1.5-3.4
Kurth and Walker (Unpublished)	Sex work (Female)	HIV	6.7	1.8-17.0	6.9	1.4-20.2
Kurth and Walker (Unpublished)	Sex work (Male)	HIV	2.4	0.1-13.3	2.2	1.4-3.3
Maher (Unpublished)	Male-with-male sex	HCV	6.2	4.7-8.1	7.2	6.5-7.9
Maher (Unpublished)	Male-with-male sex	HIV	0.6	0.3-1.0	0.09	0.0-0.0
Martinello (2023)	Male-with-male sex	HCV	21.7	16.7-44.4	21.1	13.1-33.9
Mehta (2006)	Male-with-male sex	HIV	-	---	-	---
Mirzazadeh (2018)	Male-with-male sex	HIV	2.6	1.1-5.2	1.5	0.3-0.0
Mitchell and Clarke (Unpublished)	Sex work (All)	HCV	-	---	-	---
Mitchell and Clarke (Unpublished)	Sex work (Female)	HCV	-	---	-	---

Study	Exposure	Outcome	Incidence/ 100 PYs among exposed	95% CI	Incidence/ 100 PYs among unexposed	95% CI
Mitchell and Clarke (Unpublished)	Sex work (Male)	HCV	-	---	-	---
Morris and Nguyen (Unpublished)	Male-with-male sex	HCV	NR	---	NR	---
Mravcik (Unpublished)	Sex work (All)	HCV	10.8	0.0-32.1	12.2	5.3-19.0
Mravcik (Unpublished)	Sex work (Female)	HCV	19.4	0.0-57.5	10.6	1.3-15.7
Mravcik (Unpublished)	Sex work (Male)	HCV	12.3	0.0-46.3	14.5	4.1-24.9
Read (2024)	Male-with-male sex	HCV	10.3	4.9-21.5	12.6	6.5-24.2
Scheim (2018)	Male-with-male sex	HIV	-	---	-	---
Scheim (2018)	Sex work (Male)	HIV	NR	---	NR	---
Solomon and Mehta (Unpublished)	Male-with-male sex	HIV	10.0	6.3-13.6	5.5	4.6-3.7
Solomon and Mehta (Unpublished)	Sex work (All)	HIV	3.7	1.5-5.7	5.1	4.2-6.1
Solomon, Mehta and Clipman (Unpublished)	Male-with-male sex	HCV	NR	---	NR	---
Solomon, Mehta and Clipman (Unpublished)	Male-with-male sex	HIV	20.5	14.6-28.8	25.7	21.6-18.1
Spada (2018)	Male-with-male sex	HCV	13.8	1.9-97.6	5.6	3.4-9.3
Spittal (2012)	Sex work (All)	HCV	-	---	-	---
Strathdee, Abramovitz and Artamonova (Unpublished)	Male-with-male sex	HIV	3.2	0.0-6.6	1.3	0.3-0.0
Strathdee, Abramovitz and Artamonova (Unpublished)	Sex work (All)	HCV	18.6	11.2-26.1	33.0	---
Strathdee, Abramovitz and Artamonova (Unpublished)	Sex work (All)	HIV	2.5	0.0-4.9	1.7	0.0-3.6
Strathdee, Abramovitz and Artamonova (Unpublished)	Sex work (Female)	HCV	19.9	8.6-21.3	28.6	0.6-56.5
Strathdee, Abramovitz and Artamonova (Unpublished)	Sex work (Female)	HIV	4.8	0.0-16.8	5.7	0.0-16.8
Strathdee, Abramovitz and Artamonova (Unpublished)	Sex work (Male)	HCV	17.5	7.6-27.4	23.4	14.1-32.8
Strathdee, Abramovitz and Artamonova (Unpublished)	Sex work (Male)	HIV	1.00	0.0-3.0	1.3	0.0-3.0
Zhang (2007)	Sex work (All)	HIV	14.4	6.2-28.4	8.1	5.6-11.3
Zhang (2007)	Sex work (Male)	HIV	NR	---	NR	---

Appendix 7. Modified Newcastle-Ottawa scale

1. Representativeness of the cohort (one star)
   a) Truly representative (one star)
   b) Not representative (no star)
   d) No description of the derivation of the cohort (no star)
2. Selection of the non-exposed cohort (one star)
   a) Drawn from the same community as the exposed cohort (one star)
   b) Drawn from a different source (no star)
   c) No description of the derivation of the non-exposed cohort (no star)
3. Ascertainment of exposure (one star)
   a) Personal recall with timeframe from structured questionnaire (one star)
   b) Written self-report (no star)
   c) Not stated (no star)
4. Demonstration that outcome of interest was not present at start of study (one star)
   a) Yes (one star)
   b) No (no star)
5. Comparability of cohorts on the basis of the design or analysis controlled for confounders (one star)
   a) Data/ results adjusted for relevant predictors/risk factors/confounders e.g. age, sex work typology, etc. (one star)
   b) Cohorts are not comparable on the basis of the design or analysis controlled for confounders (no star)
6. Assessment of incident infection (one star)
   a) Details of HIV testing method given with confirmatory testing (one star)
   b) no details of HIV testing method (no star)
7. Was follow-up testing schedule adequate? (one star)
   a) defined and sufficiently long (i.e., >=1 year), or not applicable (HIV incidence estimated based on tests for recent infection) (one star)
   b) not sufficiently long (i.e., <1 month) or not defined (no star)
8. Adequacy of follow-up of cohorts (one star)
   a) complete follow-up or very small number (<=20%) lost to follow up, or not applicable (HIV incidence estimated based on tests for recent infection) (one star)
   b) more than 20% lost to follow up (no star)
   c) Not reported (no star)

Appendix 8. Details of studies excluded for duplicate data

Study	Cohort	Study period	Sample size	Exposure	Outcome
Ahamad (2015)	VIDUS	1996 – 2013	1639	Sex work	HIV
Blouin (2016)	SurvUDI	2004 – 2014	1362	Sex work	HIV
Boileau (2005)	HEPCO	1992 – 2001	1147	Sex work	HIV
Bruneau (2011)	St Luc Cohort	1992 – 2008	2137	Sex work and MSM	HIV
Corneil (2006)	VIDUS	1996 – 2003	1013	Sex work	HIV
Craib (2003)	VIDUS	1996 – 2000	941	Sex work	HIV
DeBeck (2009)	VIDUS	1996 – 2005	1048	Sex work	HIV
Hankins (2002)	SurvUDI	1995 – 2000	1386	Sex work	HIV
Hill (2020)	NR	2014 – 2019	200	MSM	HCV
Ickowicz (2015)	VIDUS	1996 – 2013	1682	Sex work	HIV
Jacka (2018a)	HEPCO	2004 – 2016	440	MSM	HCV
Jacka (2018b)	HEPCO	2004 – 2016	432	MSM	HCV
Jacka (2019a)	HEPCO	2004 – 2018	436	MSM	HCV
Jacka (2019b)	HEPCO	2004 – 2017	440	MSM	HCV
Kerr (2016)	VIDUS	1996 – 2012	1647	Sex work	HIV
Kral (2003)	Urban Health Study	1987 – 1998	8065	Sex work and MSM	HIV
Le Marchand (2015)	INC3	NR	1064	MSM	HCV
Miller (2002)	VIDUS	NR	48	Sex work	HIV
Miller (2006)	VIDUS	1996 – 2003	196	Sex work	HIV
Montain (2016)	VIDUS	1996 – 2013	1639	Sex work	HIV

Nelson (2002)	ALIVE	1988 – 1998	1846	Sex work	HIV
Patrick (2001)	VIDUS	1996 – 1999	1345	Sex work	HCV
Shannon (2010)	VIDUS	1996 – 2007	179	Sex work	HCV
Skaathun (2022)	La Frontera	NR	286	MSM	HIV
Spittal (2002)	VIDUS	1996 – 2000	939	MSM	HIV
Tyndall (2003)	VIDUS	1996 – 2000	940	Sex work	HIV
Van Den Berg (2007)	Amsterdam Cohort Study	1985 – 2005	803	Sex work	HCV
Wood (2007)	VIDUS	1996 – 2004	1035	Sex work	HIV
Young (2016)	VIDUS	1996 – 2013	1682	Sex work	HIV

Appendix 9. Meta analyses of recent exposure to sex work or male-with-male sexual activity and HIV or HCV acquisition

Meta-analysis of studies reporting the crude pooled association between recent exposure to sex work and HIV acquisition risk among male and female PWID

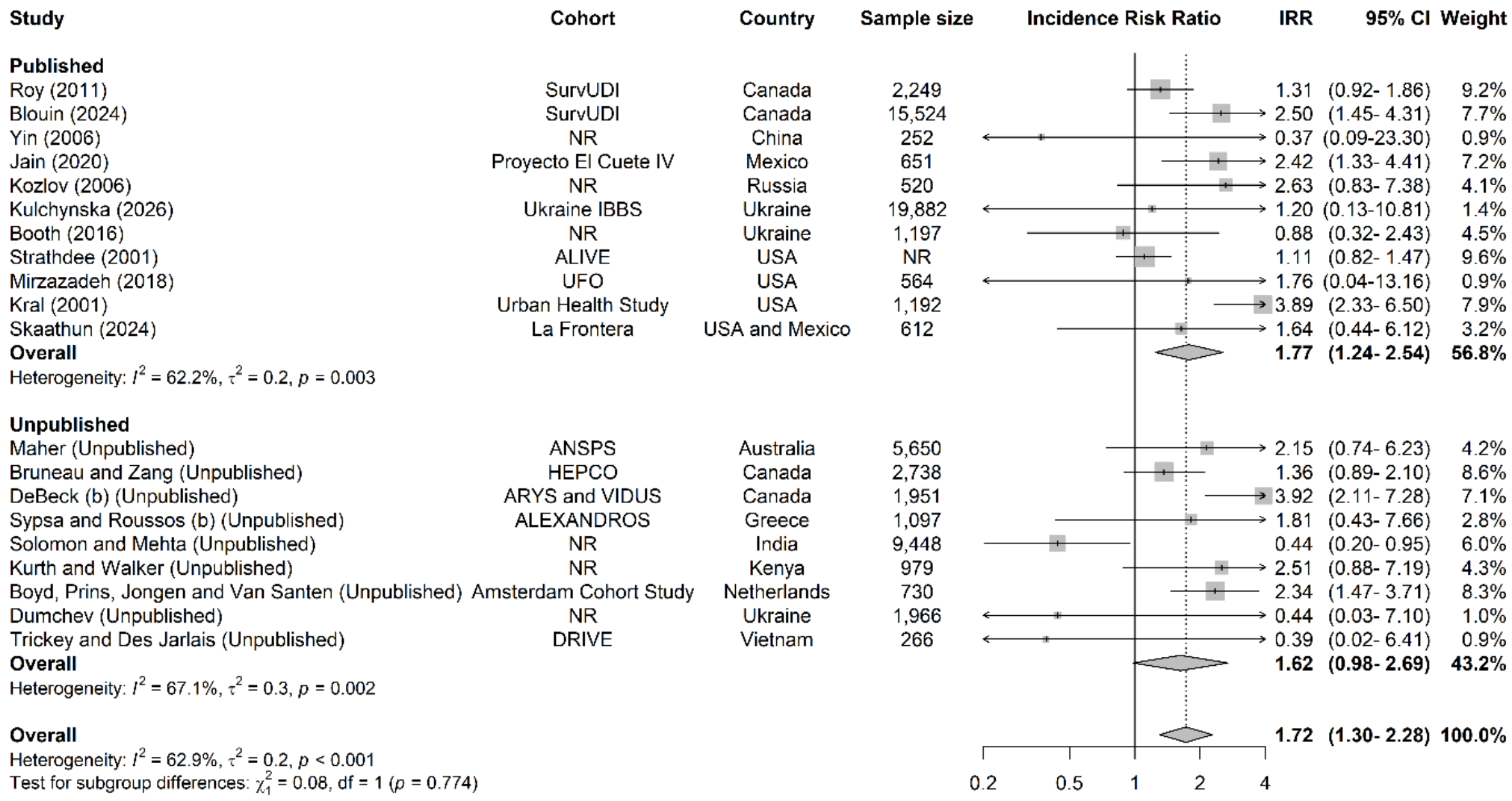

Meta-analysis of studies reporting the crude pooled association between recent exposure to sex work and HCV acquisition risk among male and female PWID

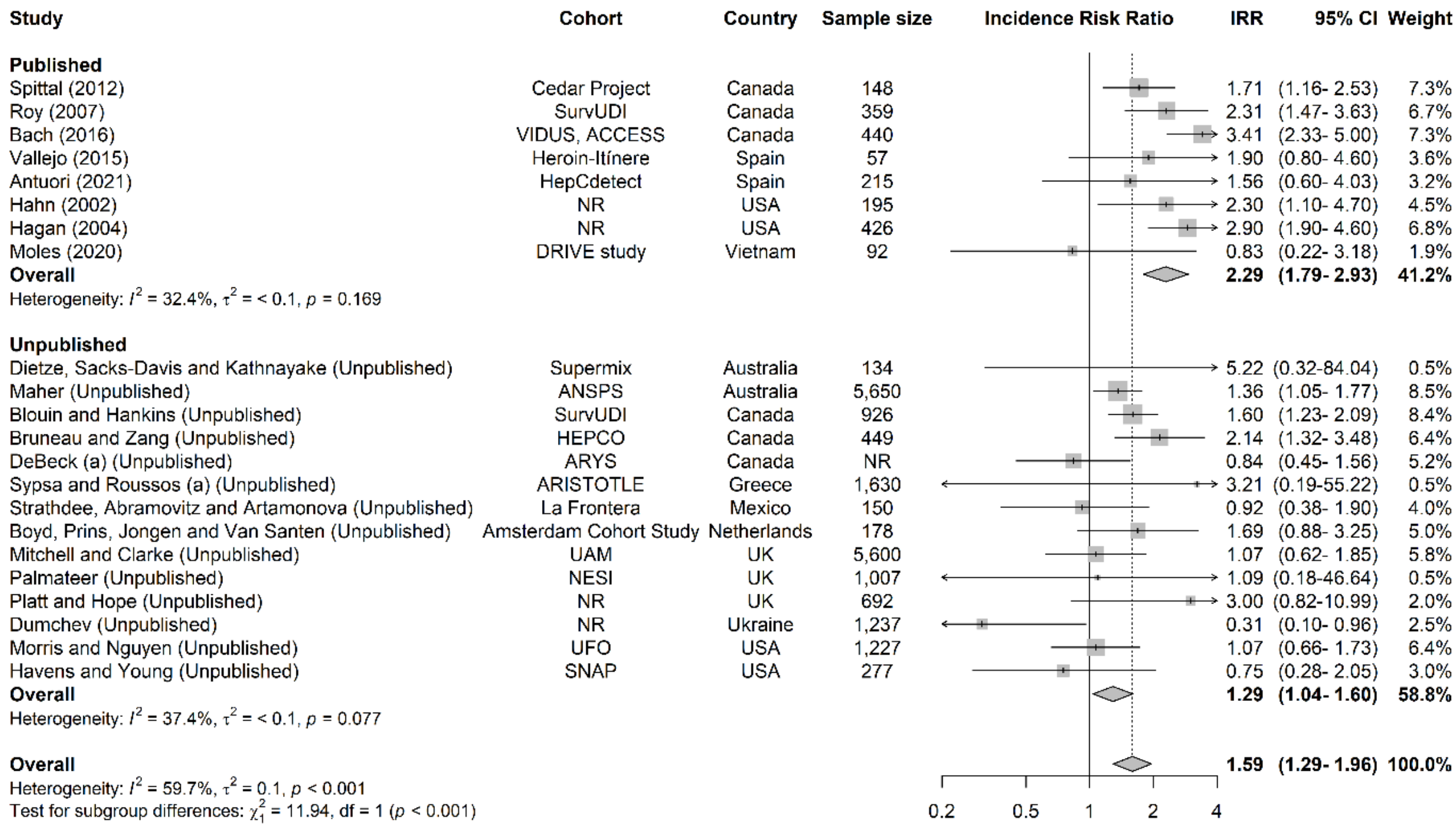

Meta-analysis of studies reporting the crude pooled association between recent exposure to sex work and HIV acquisition risk among male PWID

Study	Cohort	Country	Sample size	IRR	95% CI	Weight
**Unpublished**						
Maher (Unpublished)	ANSPS	Australia	3,571	5.19	(1.53- 17.55)	8.0%
Blouin and Hankins (Unpublished)	SurvUDI	Canada	1,631	1.42	(0.57- 3.54)	11.0%
Bruneau and Zang (Unpublished)	HEPCO	Canada	2,213	1.82	(1.03- 3.21)	15.6%
Sarna and Ray Saraswati (Unpublished)	AVHI	India	3,723	0.74	(0.24- 2.30)	8.8%
Solomon and Mehta (Unpublished)	NR	India	9,169	0.48	(0.22- 1.05)	12.7%
Kurth and Walker (Unpublished)	NR	Kenya	899	1.13	(0.07- 18.57)	2.2%
Strathdee, Abramovitz and Artamonova (Unpublished)	La Frontera	Mexico	122	9.70	(0.90-107.10)	2.9%
Boyd, Prins, Jongen and Van Santen (Unpublished)	Amsterdam Cohort Study	Netherlands	448	1.94	(0.47- 7.96)	6.6%
Dumchev (Unpublished)	NR	Ukraine	1,442	10.98	(0.67-180.86)	2.2%
**Overall**				**1.63**	**(0.89- 3.01)**	**70.1%**
Heterogeneity: $I^2 = 56.5\%$, $\tau^2 = 0.4$, $p = 0.018$						
**Published**						
Kozlov (2006)	NR	Russia	365	1.34	(0.06- 7.64)	2.8%
Kral (2001)	Urban Health Study	USA	806	1.95	(0.62- 6.15)	8.6%
Strathdee (2001)	ALIVE	USA	NR	0.91	(0.63- 1.31)	18.4%
**Overall**				**0.98**	**(0.70- 1.39)**	**29.9%**
Heterogeneity: $I^2 = 0\%$, $\tau^2 = 0$, $p = 0.449$						
**Overall**				**1.43**	**(0.92- 2.22)**	**100.0%**
Heterogeneity: $I^2 = 50.7\%$, $\tau^2 = 0.2$, $p = 0.022$						
Test for subgroup differences: $\chi^2_1 = 2.02$, df = 1 ($p = 0.155$)						

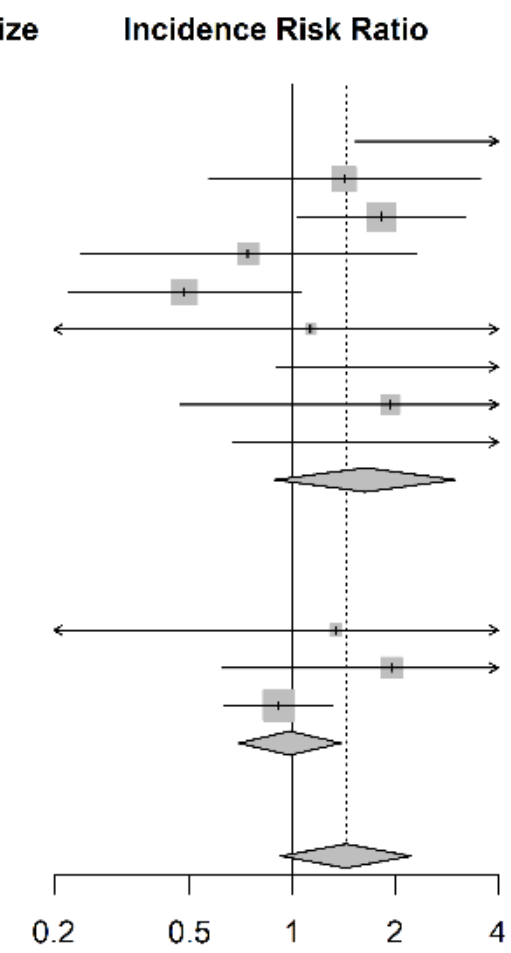

Meta-analysis of studies reporting the crude pooled association between recent exposure to sex work and HIV acquisition risk among female PWID

Study	Cohort	Country	Sample size	IRR	95% CI	Weight
**Unpublished**						
Maher (Unpublished)	ANSPS	Australia	2,079	1.73	(0.18- 16.77)	3.4%
DeBeck (b) (Unpublished)	ARYS and VIDUS	Canada	686	4.55	(2.00- 10.38)	10.6%
Blouin and Hankins (Unpublished)	SurvUDI	Canada	554	2.77	(1.23- 6.23)	10.7%
Bruneau and Zang (Unpublished)	HEPCO	Canada	525	2.99	(1.16- 7.72)	9.6%
Kurth and Walker (Unpublished)	NR	Kenya	80	1.59	(0.34- 7.11)	5.9%
Strathdee, Abramovitz and Artamonova (Unpublished)	La Frontera	Mexico	44	1.14	(0.16- 8.08)	4.2%
Boyd, Prins, Jongen and Van Santen (Unpublished)	Amsterdam Cohort Study	Netherlands	282	2.81	(1.47- 5.39)	12.1%
Dumchev (Unpublished)	NR	Ukraine	524	0.26	(0.02- 4.41)	2.5%
**Overall**				**2.76**	**(1.91- 3.97)**	**59.1%**
Heterogeneity: $I^2 = 0\%$, $\tau^2 = 0$, $p = 0.559$						
**Published**						
Sypsa (2017)	ARISTOTLE	Greece	NR	1.02	(0.77- 1.43)	14.8%
Kozlov (2006)	NR	Russia	155	10.16	(1.41-241.83)	2.7%
Kral (2001)	Urban Health Study	USA	369	5.32	(2.52- 11.24)	11.3%
Strathdee (2001)	ALIVE	USA	NR	1.65	(0.86- 3.16)	12.1%
**Overall**				**2.30**	**(0.93- 5.67)**	**40.9%**
Heterogeneity: $I^2 = 84\%$, $\tau^2 = 0.6$, $p < 0.001$						
**Overall**				**2.30**	**(1.44- 3.68)**	**100.0%**
Heterogeneity: $I^2 = 67.5\%$, $\tau^2 = 0.4$, $p < 0.001$						
Test for subgroup differences: $\chi^2_1 = 0.13$, df = 1 ($p = 0.716$)						

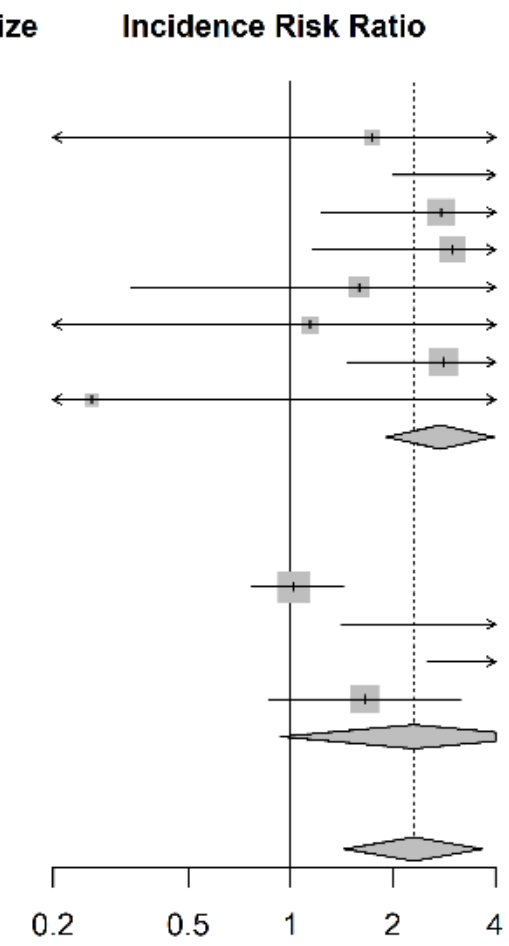

Meta-analysis of studies reporting the crude pooled association between recent exposure to sex work and HCV acquisition risk among male PWID

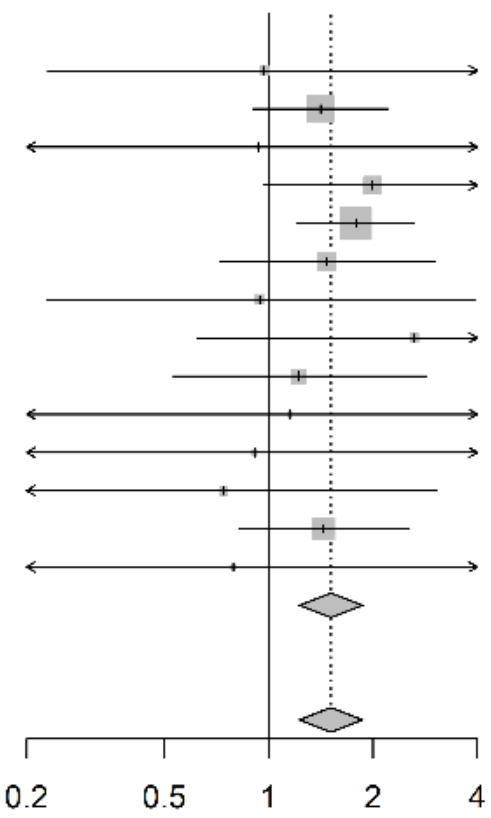

Study	Cohort	Country	Sample size	IRR	95% CI	Weight
**Unpublished**						
Todd (Unpublished)	NR	Afghanistan	479	0.97	(0.23- 4.13)	2.1%
Maher (Unpublished)	ANSPS	Australia	1,466	1.41	(0.90- 2.21)	22.0%
Dietze, Sacks-Davis and Kathnayake (Unpublished)	SuperMIX	Australia	95	0.93	(0.02-47.29)	0.3%
Bruneau and Zang (Unpublished)	HEPCO	Canada	361	1.99	(0.97- 4.08)	8.6%
Blouin and Hankins (Unpublished)	SurvUDI	Canada	687	1.78	(1.21- 2.63)	29.5%
DeBeck (b) (Unpublished)	ARYS and VIDUS	Canada	524	1.47	(0.72- 3.01)	8.7%
Strathdee, Abramovitz and Artamonova (Unpublished)	La Frontera	Mexico	103	0.94	(0.23- 3.92)	2.2%
Boyd, Prins, Jongen and Van Santen (Unpublished)	Amsterdam Cohort Study	Netherlands	119	2.63	(0.62-11.06)	2.1%
Mitchell and Clarke (Unpublished)	UAM	UK	4,186	1.22	(0.53- 2.84)	6.3%
Platt and Hope (Unpublished)	NR	UK	540	1.15	(0.07-18.97)	0.6%
Dumchev (Unpublished)	NR	Ukraine	903	0.91	(0.06-14.56)	0.6%
Havens and Young (Unpublished)	SNAP	USA	147	0.74	(0.18- 3.04)	2.2%
Morris and Nguyen (Unpublished)	UFO	USA	845	1.44	(0.82- 2.52)	14.1%
Trickey and Des Jarlais (Unpublished)	NR	Vietnam	243	0.79	(0.05-12.98)	0.6%
**Overall**				**1.51**	**(1.22- 1.87)**	**100.0%**
Heterogeneity: I^2 = 0%, τ^2 = 0, p = 0.986						
**Overall**				**1.51**	**(1.22- 1.87)**	**100.0%**
Heterogeneity: I^2 = 0.0%, τ^2 = 0, p = 0.986						
Test for subgroup differences: χ^2_0 = 0.00, df = 0 (p = NA)						

Incidence Risk Ratio (axis: 0.2, 0.5, 1, 2, 4)

Meta-analysis of studies reporting the crude pooled association between recent exposure to sex work and HCV acquisition risk among female PWID

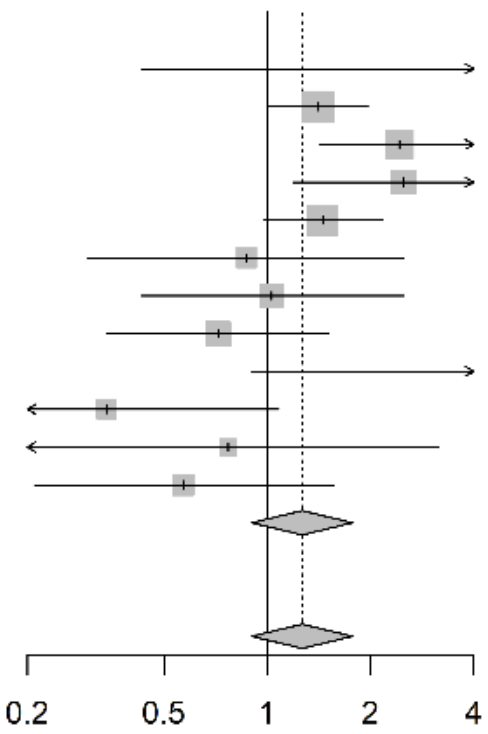

Study	Cohort	Country	Sample size	IRR	95% CI	Weight
**Unpublished**						
Dietze, Sacks-Davis and Kathnayake (Unpublished)	SuperMIX	Australia	39	13.87	(0.43-445.28)	0.9%
Maher (Unpublished)	ANSPS	Australia	867	1.41	(1.01- 1.97)	16.0%
DeBeck (b) (Unpublished)	ARYS and VIDUS	Canada	266	2.43	(1.43- 4.15)	12.9%
Bruneau and Zang (Unpublished)	HEPCO	Canada	88	2.50	(1.19- 5.28)	9.9%
Blouin and Hankins (Unpublished)	SurvUDI	Canada	239	1.45	(0.98- 2.17)	15.1%
Strathdee, Abramovitz and Artamonova (Unpublished)	La Frontera	Mexico	47	0.87	(0.30- 2.50)	6.6%
Boyd, Prins, Jongen and Van Santen (Unpublished)	Amsterdam Cohort Study	Netherlands	59	1.03	(0.43- 2.49)	8.3%
Mitchell and Clarke (Unpublished)	UAM	UK	1,414	0.72	(0.34- 1.51)	9.9%
Platt and Hope (Unpublished)	NR	UK	152	5.61	(0.90- 35.08)	2.9%
Dumchev (Unpublished)	NR	Ukraine	334	0.34	(0.11- 1.08)	6.0%
Havens and Young (Unpublished)	SNAP	USA	130	0.77	(0.18- 3.17)	4.3%
Morris and Nguyen (Unpublished)	UFO	USA	382	0.57	(0.21- 1.57)	7.1%
**Overall**				**1.26**	**(0.90- 1.77)**	**100.0%**
Heterogeneity: I^2 = 54.7%, τ^2 = 0.2, p = 0.012						
**Overall**				**1.26**	**(0.90- 1.77)**	**100.0%**
Heterogeneity: I^2 = 54.7%, τ^2 = 0.2, p = 0.012						
Test for subgroup differences: χ^2_0 = 0.00, df = 0 (p = NA)						

Incidence Risk Ratio (axis: 0.2, 0.5, 1, 2, 4)

Meta-analysis of studies reporting the adjusted pooled association between recent exposure to sex work and HIV acquisition risk among male and female PWID

Study	Cohort	Country	Sample size	IRR	95% CI	Weight
**Published**						
Roy (2011)	SurvUDI	Canada	2,249	1.37	(0.93-2.03)	13.7%
Blouin (2024)	SurvUDI	Canada	15,524	2.23	(1.46-3.42)	13.2%
Jain (2020)	Proyecto El Cuete IV	Mexico	651	2.25	(1.05-4.80)	8.5%
Kozlov (2006)	NR	Russia	520	1.86	(0.65-5.26)	5.9%
**Overall**				**1.78**	**(1.36-2.34)**	**41.3%**
Heterogeneity: $I^2 = 5.6\%$, $\tau^2 = < 0.1$, $p = 0.365$						
**Unpublished**						
Maher (Unpublished)	ANSPS	Australia	5,650	2.09	(0.72-6.07)	5.7%
Bruneau and Zang (Unpublished)	HEPCO	Canada	2,738	1.39	(0.90-2.14)	13.1%
DeBeck (b) (Unpublished)	ARYS and VIDUS	Canada	1,951	3.80	(2.03-7.12)	10.2%
Sypsa and Roussos (b) (Unpublished)	ALEXANDROS	Greece	1,097	1.81	(0.43-7.66)	3.7%
Solomon and Mehta (Unpublished)	NR	India	9,448	0.43	(0.20-0.90)	8.7%
Kurth and Walker (Unpublished)	NR	Kenya	979	2.32	(0.81-6.65)	5.8%
Boyd, Prins, Jongen and Van Santen (Unpublished)	Amsterdam Cohort Study	Netherlands	730	1.95	(1.14-3.31)	11.6%
**Overall**				**1.66**	**(0.99-2.80)**	**58.7%**
Heterogeneity: $I^2 = 71.3\%$, $\tau^2 = 0.3$, $p = 0.002$						
**Overall**				**1.73**	**(1.27-2.36)**	**100.0%**
Heterogeneity: $I^2 = 58.8\%$, $\tau^2 = 0.1$, $p = 0.007$						
Test for subgroup differences: $\chi^2_1 = 0.06$, df = 1 ($p = 0.810$)						

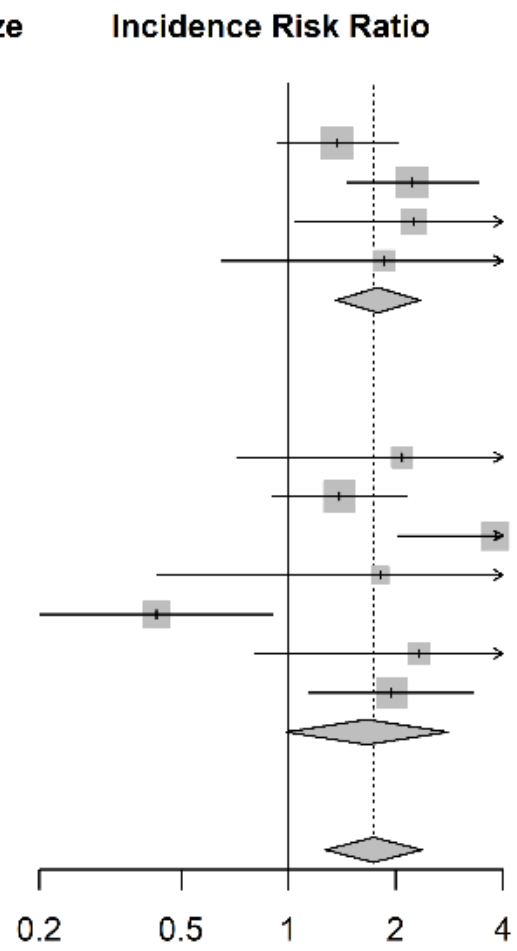

Meta-analysis of studies reporting the adjusted pooled association between recent exposure to sex work and HCV acquisition risk among male and female PWID

Study	Cohort	Country	Sample size	IRR	95% CI	Weight
**Published**						
Spittal (2012)	Cedar Project	Canada	148	1.59	(1.05- 2.42)	11.6%
Roy (2007)	SurvUDI	Canada	359	2.61	(1.64- 4.16)	10.7%
**Overall**				**2.01**	**(1.24- 3.27)**	**22.3%**
Heterogeneity: $I^2 = 58.6\%$, $\tau^2 = < 0.1$, $p = 0.120$						
**Unpublished**						
Maher (Unpublished)	ANSPS	Australia	5,650	1.32	(1.02- 1.72)	15.1%
Blouin and Hankins (Unpublished)	SurvUDI	Canada	926	1.49	(1.14- 1.95)	14.9%
Bruneau and Zang (Unpublished)	HEPCO	Canada	449	2.13	(1.31- 3.45)	10.3%
Strathdee, Abramovitz and Artamonova (Unpublished)	La Frontera	Mexico	150	1.04	(0.43- 2.19)	5.7%
Boyd, Prins, Jongen and Van Santen (Unpublished)	Amsterdam Cohort Study	Netherlands	178	1.79	(0.86- 3.76)	6.5%
Mitchell and Clarke (Unpublished)	UAM	UK	5,600	0.82	(0.44- 1.55)	7.9%
Platt and Hope (Unpublished)	NR	UK	692	4.07	(1.02-16.21)	2.5%
Dumchev (Unpublished)	NR	Ukraine	1,237	0.32	(0.08- 0.83)	3.3%
Morris and Nguyen (Unpublished)	UFO	USA	1,227	1.06	(0.56- 2.01)	7.7%
Havens and Young (Unpublished)	SNAP	USA	277	0.84	(0.25- 2.01)	3.9%
**Overall**				**1.30**	**(1.01- 1.67)**	**77.7%**
Heterogeneity: $I^2 = 46.8\%$, $\tau^2 = < 0.1$, $p = 0.050$						
**Overall**				**1.43**	**(1.13- 1.80)**	**100.0%**
Heterogeneity: $I^2 = 53.9\%$, $\tau^2 < 0.1$, $p = 0.013$						
Test for subgroup differences: $\chi^2_1 = 2.48$, df = 1 ($p = 0.115$)						

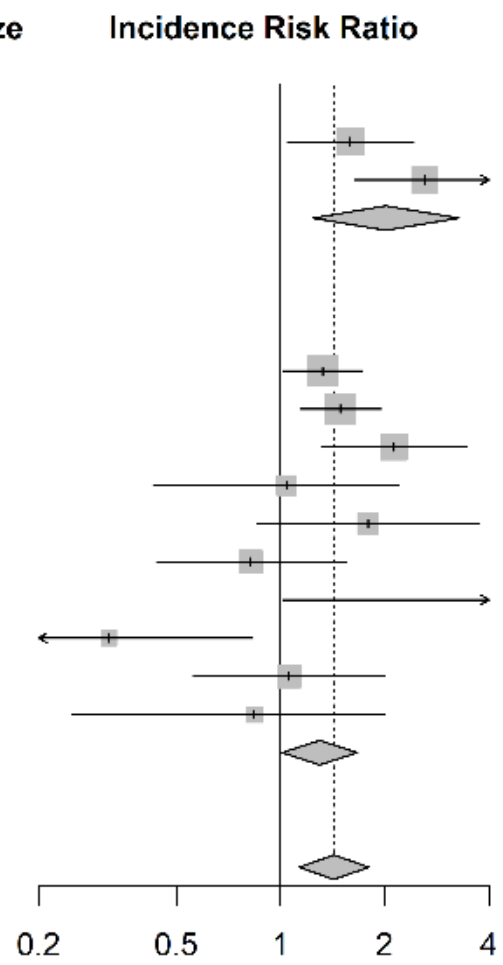

Meta-analysis of studies reporting the adjusted pooled association between recent exposure to sex work and HIV acquisition risk among male PWID

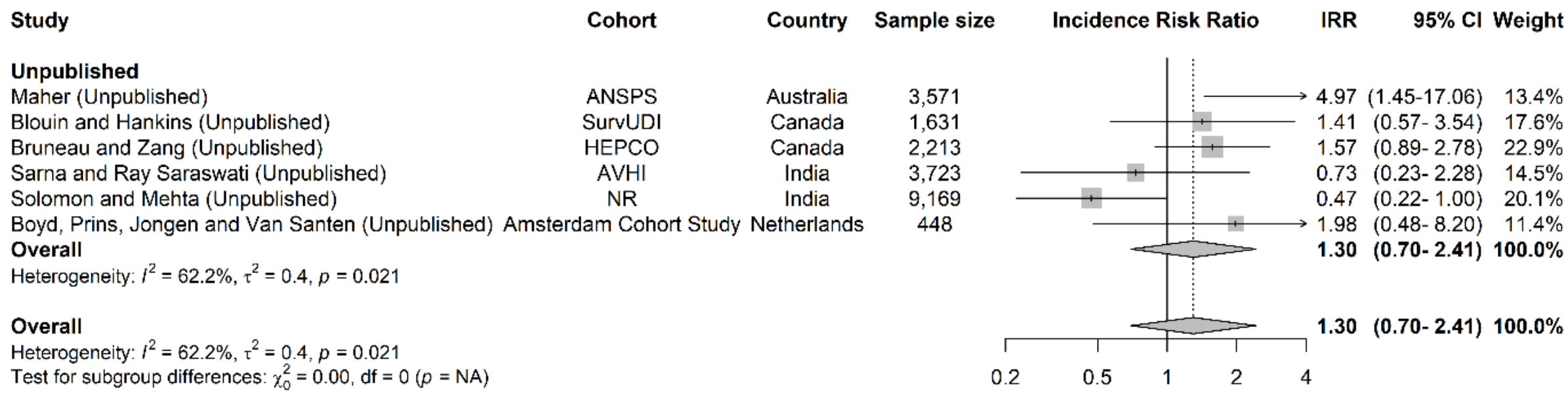

Meta-analysis of studies reporting the adjusted pooled association between recent exposure to sex work and HIV acquisition risk among female PWID

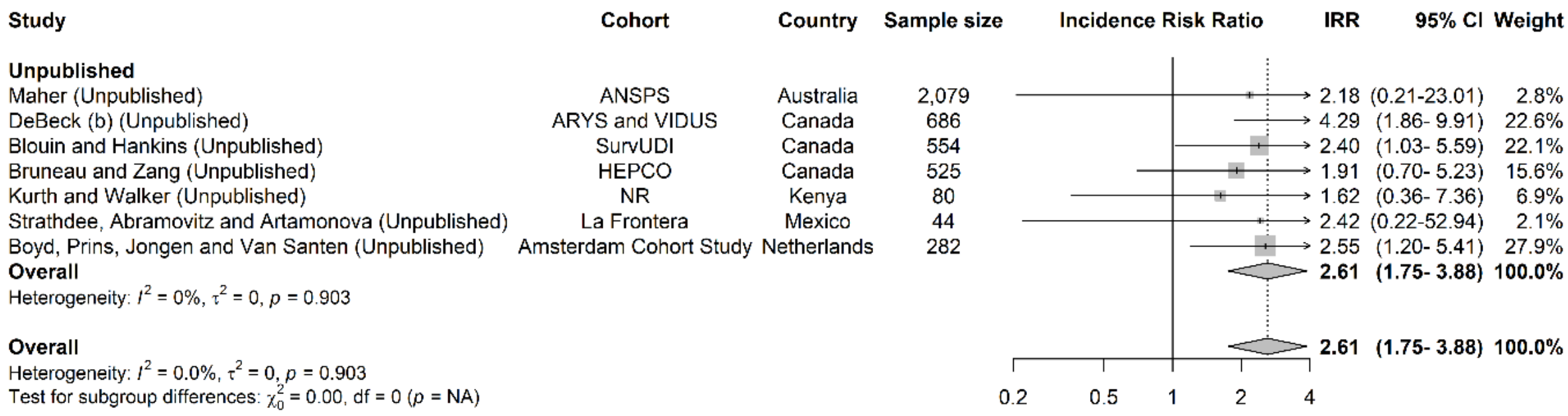

Meta-analysis of studies reporting the adjusted pooled association between recent exposure to sex work and HCV acquisition risk among male PWID

Study	Cohort	Country	Sample size	IRR	95% CI	Weight
**Unpublished**						
Todd (Unpublished)	NR	Afghanistan	479	1.07	(0.25- 4.54)	2.3%
Maher (Unpublished)	ANSPS	Australia	1,466	1.32	(0.84- 2.07)	23.3%
Bruneau and Zang (Unpublished)	HEPCO	Canada	361	1.79	(0.86- 3.70)	8.9%
Blouin and Hankins (Unpublished)	SurvUDI	Canada	687	1.68	(1.13- 2.49)	30.3%
DeBeck (b) (Unpublished)	ARYS and VIDUS	Canada	524	1.24	(0.60- 2.52)	9.2%
Strathdee, Abramovitz and Artamonova (Unpublished)	La Frontera	Mexico	103	1.01	(0.16- 3.31)	2.1%
Boyd, Prins, Jongen and Van Santen (Unpublished)	Amsterdam Cohort Study	Netherlands	119	2.76	(0.64-11.86)	2.2%
Mitchell and Clarke (Unpublished)	UAM	UK	4,186	1.37	(0.58- 3.22)	6.4%
Havens and Young (Unpublished)	SNAP	USA	147	0.82	(0.13- 2.73)	2.0%
Morris and Nguyen (Unpublished)	UFO	USA	845	1.45	(0.80- 2.63)	13.4%
**Overall**				**1.47**	**(1.18- 1.82)**	**100.0%**
Heterogeneity: $I^2 = 0\%$, $\tau^2 = 0$, $p = 0.969$						
**Overall**				**1.47**	**(1.18- 1.82)**	**100.0%**
Heterogeneity: $I^2 = 0.0\%$, $\tau^2 = 0$, $p = 0.969$						
Test for subgroup differences: $\chi^2_0 = 0.00$, df = 0 (p = NA)						

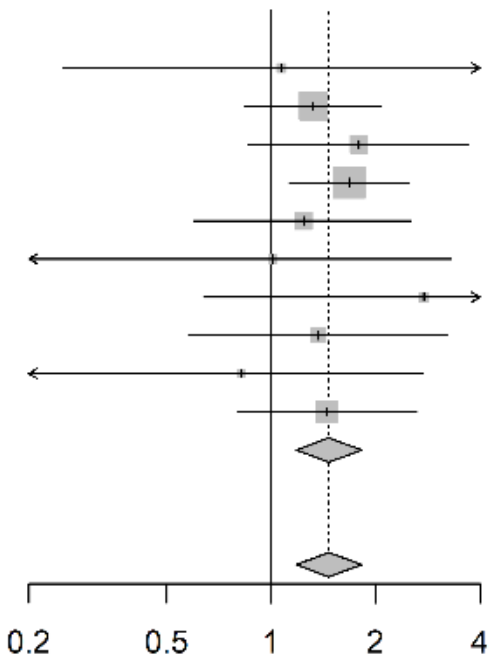

Meta-analysis of studies reporting the adjusted pooled association between recent exposure to sex work and HCV acquisition risk among female PWID

Study	Cohort	Country	Sample size	IRR	95% CI	Weight
**Unpublished**						
Maher (Unpublished)	ANSPS	Australia	867	1.38	(0.99- 1.93)	19.7%
DeBeck (b) (Unpublished)	ARYS and VIDUS	Canada	266	1.74	(0.99- 3.05)	14.2%
Bruneau and Zang (Unpublished)	HEPCO	Canada	88	2.52	(1.17- 5.46)	10.3%
Blouin and Hankins (Unpublished)	SurvUDI	Canada	239	1.33	(0.87- 2.01)	17.6%
Strathdee, Abramovitz and Artamonova (Unpublished)	La Frontera	Mexico	47	0.85	(0.26- 2.36)	6.4%
Boyd, Prins, Jongen and Van Santen (Unpublished)	Amsterdam Cohort Study	Netherlands	59	1.34	(0.46- 3.94)	6.7%
Mitchell and Clarke (Unpublished)	UAM	UK	1,414	0.54	(0.23- 1.25)	9.2%
Platt and Hope (Unpublished)	NR	UK	152	4.79	(0.76-30.33)	2.7%
Dumchev (Unpublished)	NR	Ukraine	334	0.30	(0.07- 0.82)	5.4%
Havens and Young (Unpublished)	SNAP	USA	130	0.75	(0.12- 2.47)	3.9%
Morris and Nguyen (Unpublished)	UFO	USA	382	0.52	(0.11- 2.39)	3.8%
**Overall**				**1.20**	**(0.87- 1.66)**	**100.0%**
Heterogeneity: $I^2 = 44.3\%$, $\tau^2 = 0.1$, $p = 0.056$						
**Overall**				**1.20**	**(0.87- 1.66)**	**100.0%**
Heterogeneity: $I^2 = 44.3\%$, $\tau^2 = 0.1$, $p = 0.056$						
Test for subgroup differences: $\chi^2_0 = 0.00$, df = 0 (p = NA)						

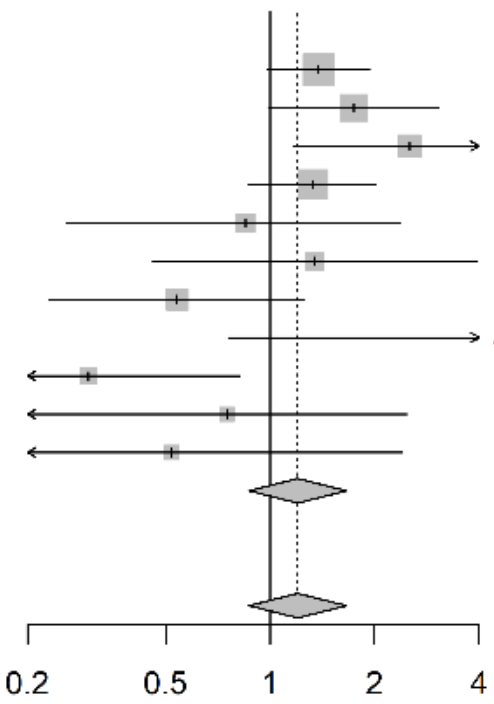

Meta-analysis of studies reporting the crude pooled association between recent exposure to male-with-male sex and HIV acquisition risk among male PWID

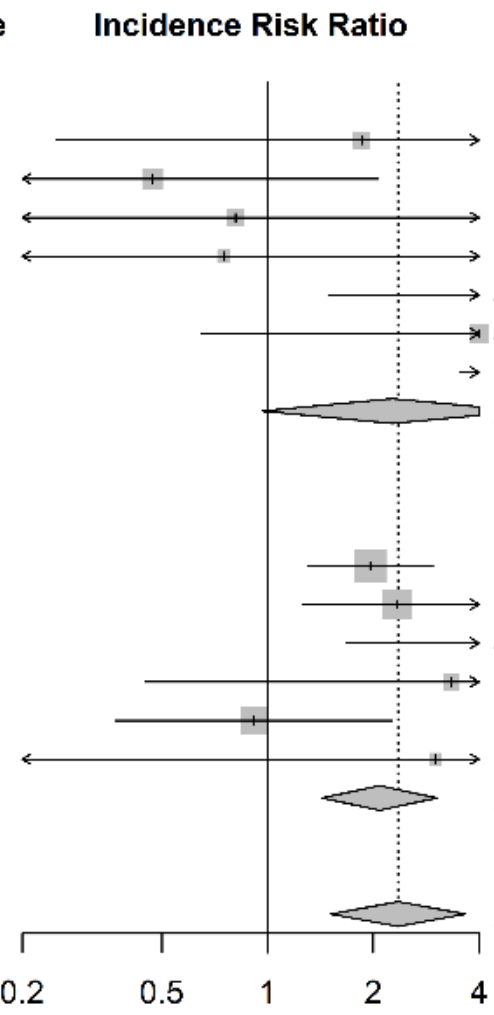

Study	Cohort	Country	Sample size	IRR	95% CI	Weight
**Published**						
Sypsa (2017)	ARISTOTLE	Greece	NR	1.85	(0.25-13.50)	3.9%
Lucas (2015)	NR	India	NR	0.47	(0.11- 2.07)	6.1%
Suntharasamai (2009)	AIDSVAX B/E HIV	Thailand	NR	0.81	(0.11- 5.87)	3.9%
Martin (2014)	Bangkok Tenofovir Study	Thailand	1,913	0.75	(0.05-12.20)	2.3%
Strathdee (2001)	ALIVE	USA	NR	4.04	(1.50-10.87)	9.8%
Mirzazadeh (2018)	UFO	USA	369	4.00	(0.65-18.89)	5.1%
Kral (2001)	Urban Health Study	USA	806	6.90	(3.52-13.49)	13.3%
**Overall**				**2.26**	**(0.97- 5.30)**	**44.3%**
Heterogeneity: I^2 = 59.7%, τ^2 = 0.7, p = 0.021						
**Unpublished**						
Bruneau and Zang (Unpublished)	HEPCO	Canada	2,213	1.96	(1.30- 2.95)	16.4%
Blouin and Hankins (Unpublished)	SurvUDI	Canada	1,633	2.34	(1.26- 4.36)	13.9%
DeBeck (b) (Unpublished)	ARYS and VIDUS	Canada	1,216	4.89	(1.67-14.32)	9.0%
Sypsa and Roussos (b) (Unpublished)	ALEXANDROS	Greece	952	3.33	(0.45-24.75)	3.9%
Solomon and Mehta (Unpublished)	NR	India	9,170	0.91	(0.37- 2.27)	10.6%
Strathdee, Abramovitz and Artamonova (Unpublished)	La Frontera	Mexico	126	3.00	(0.15-57.92)	2.0%
**Overall**				**2.08**	**(1.43- 3.03)**	**55.7%**
Heterogeneity: I^2 = 17.5%, τ^2 = < 0.1, p = 0.300						
**Overall**				**2.35**	**(1.51- 3.66)**	**100.0%**
Heterogeneity: I^2 = 51.3%, τ^2 = 0.3, p = 0.017						
Test for subgroup differences: χ^2_1 = 0.03, df = 1 (p = 0.860)						

Meta-analysis of studies reporting the crude pooled association between recent exposure to male-with-male sex and HCV acquisition risk among male PWID

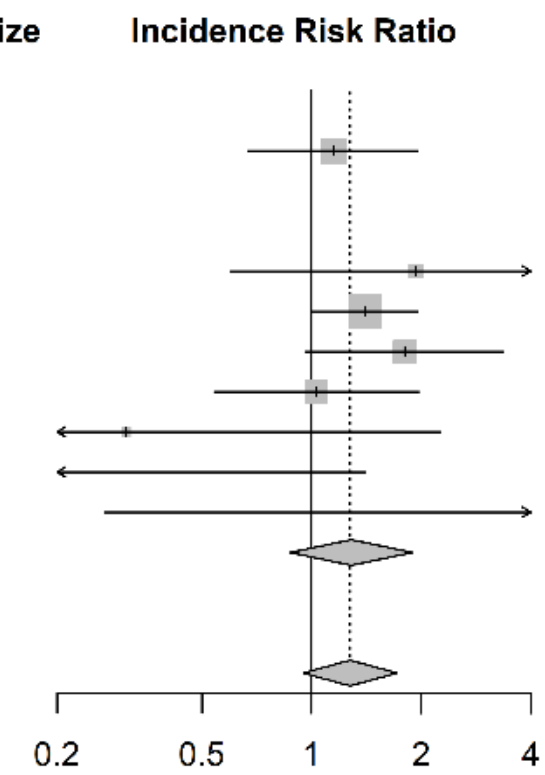

Study	Cohort	Country	Sample size	IRR	95% CI	Weight
**Published**						
Roy (2007)	SurvUDI	Canada	NR	1.15	(0.67- 1.96)	20.6%
**Unpublished**						
Todd (Unpublished)	NR	Afghanistan	479	1.93	(0.60- 6.16)	5.9%
Blouin and Hankins (Unpublished)	SurvUDI	Canada	689	1.40	(1.01- 1.95)	35.5%
Bruneau and Zang (Unpublished)	HEPCO	Canada	361	1.80	(0.96- 3.36)	16.6%
DeBeck (b) (Unpublished)	ARYS and VIDUS	Canada	521	1.03	(0.54- 1.97)	15.8%
Strathdee, Abramovitz and Artamonova (Unpublished)	La Frontera	Mexico	103	0.31	(0.04- 2.26)	2.1%
Mitchell and Clarke (Unpublished)	UAM	UK	4,631	0.19	(0.03- 1.40)	2.3%
Platt and Hope (Unpublished)	NR	UK	731	4.20	(0.27-64.90)	1.2%
**Overall**				**1.28**	**(0.87- 1.89)**	**79.4%**
Heterogeneity: I^2 = 30.2%, τ^2 = < 0.1, p = 0.198						
**Overall**				**1.28**	**(0.95- 1.72)**	**100.0%**
Heterogeneity: I^2 = 21.0%, τ^2 < 0.1, p = 0.263						
Test for subgroup differences: χ^2_1 = 0.11, df = 1 (p = 0.743)						

Meta-analysis of studies reporting the adjusted pooled association between recent exposure to male-with-male sex and HIV acquisition risk among male PWID

Study	Cohort	Country	Sample size	IRR	95% CI	Weight
**Published**						
Kral (2001)	Urban Health Study	USA	806	8.80	(3.70-20.50)	17.6%
**Unpublished**						
Bruneau and Zang (Unpublished)	HEPCO	Canada	2,213	1.73	(1.14- 2.61)	23.5%
Blouin and Hankins (Unpublished)	SurvUDI	Canada	1,633	2.39	(1.27- 4.47)	20.7%
DeBeck (b) (Unpublished)	ARYS and VIDUS	Canada	1,216	3.70	(1.25-11.00)	14.6%
Sypsa and Roussos (b) (Unpublished)	ALEXANDROS	Greece	952	6.40	(0.77-53.30)	6.5%
Solomon and Mehta (Unpublished)	NR	India	9,170	0.87	(0.36- 2.12)	17.1%
**Overall**				**1.95**	**(1.25- 3.06)**	**82.4%**
Heterogeneity: I^2 = 37.2%, τ^2 = < 0.1, p = 0.173						
**Overall**				**2.66**	**(1.43- 4.98)**	**100.0%**
Heterogeneity: I^2 = 71.3%, τ^2 = 0.4, p = 0.004						
Test for subgroup differences: χ^2_1 = 9.33, df = 1 (p = 0.002)						

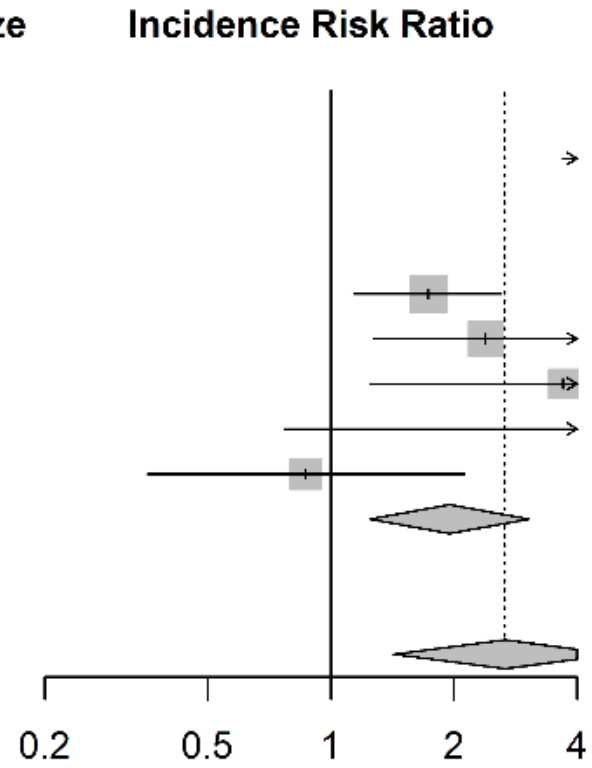

Meta-analysis of studies reporting the adjusted pooled association between recent exposure to male-with-male sex and HCV acquisition risk among male PWID

Study	Cohort	Country	Sample size	IRR	95% CI	Weight
**Unpublished**						
Todd (Unpublished)	NR	Afghanistan	479	2.15	(0.66-6.96)	10.8%
Blouin and Hankins (Unpublished)	SurvUDI	Canada	689	1.43	(1.03-1.99)	35.6%
Bruneau and Zang (Unpublished)	HEPCO	Canada	361	1.76	(0.94-3.30)	23.5%
DeBeck (b) (Unpublished)	ARYS and VIDUS	Canada	521	0.73	(0.38-1.41)	22.5%
Strathdee, Abramovitz and Artamonova (Unpublished)	La Frontera	Mexico	103	0.44	(0.02-2.65)	3.1%
Mitchell and Clarke (Unpublished)	UAM	UK	4,631	0.21	(0.03-1.56)	4.5%
**Overall**				**1.19**	**(0.76-1.86)**	**100.0%**
Heterogeneity: I^2 = 43.9%, τ^2 = 0.1, p = 0.112						
**Overall**				**1.19**	**(0.76-1.86)**	**100.0%**
Heterogeneity: I^2 = 43.9%, τ^2 = 0.1, p = 0.112						
Test for subgroup differences: χ^2_0 = 0.00, df = 0 (p = NA)						

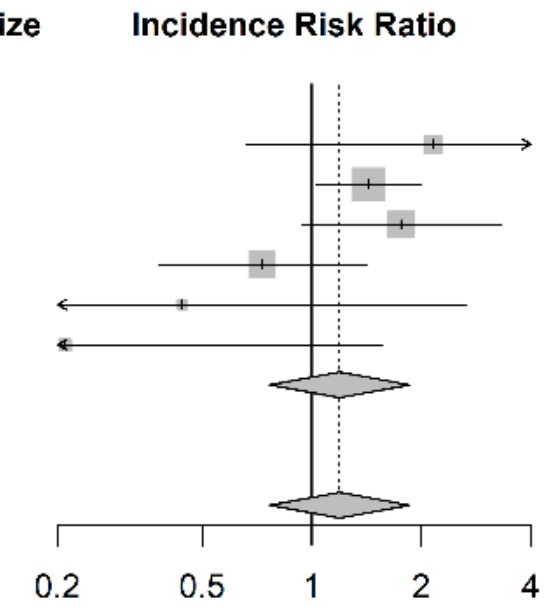

Appendix 10. Meta analyses of lifetime exposure to sex work or male-with-male sexual activity and HIV or HCV acquisition

Meta-analysis of studies reporting the crude pooled association between lifetime exposure to sex work and HIV acquisition risk among male and female PWID

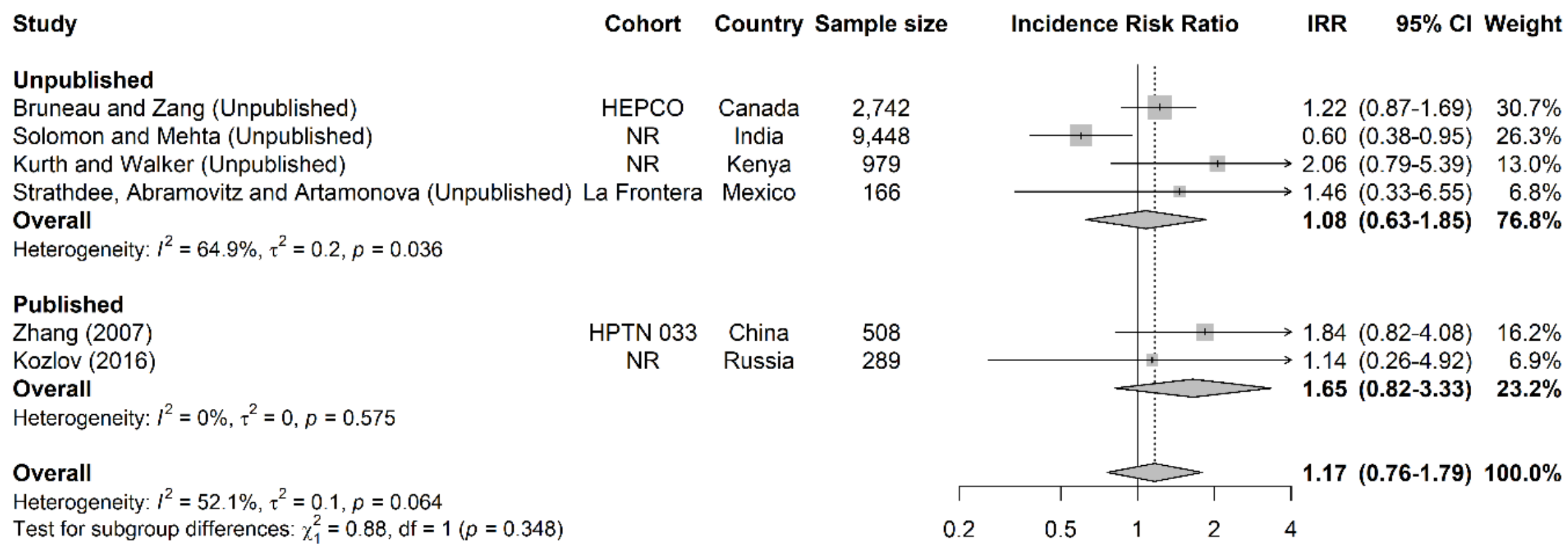

Meta-analysis of studies reporting the crude pooled association between lifetime exposure to sex work and HCV acquisition risk among male and female PWID

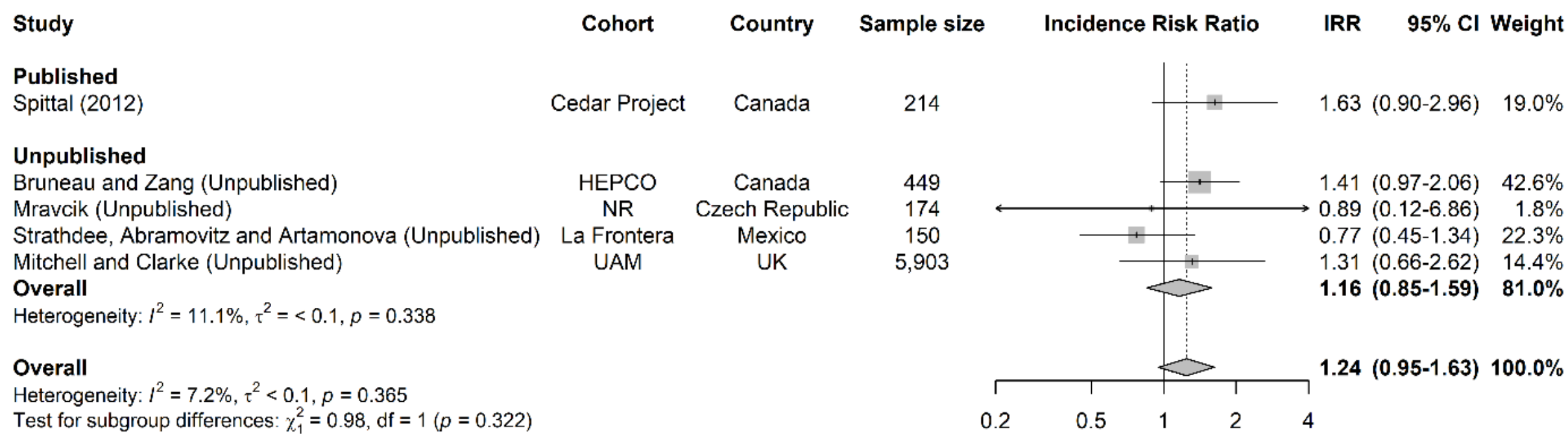

Meta-analysis of studies reporting the crude pooled association between lifetime exposure to sex work and HIV acquisition risk among male PWID

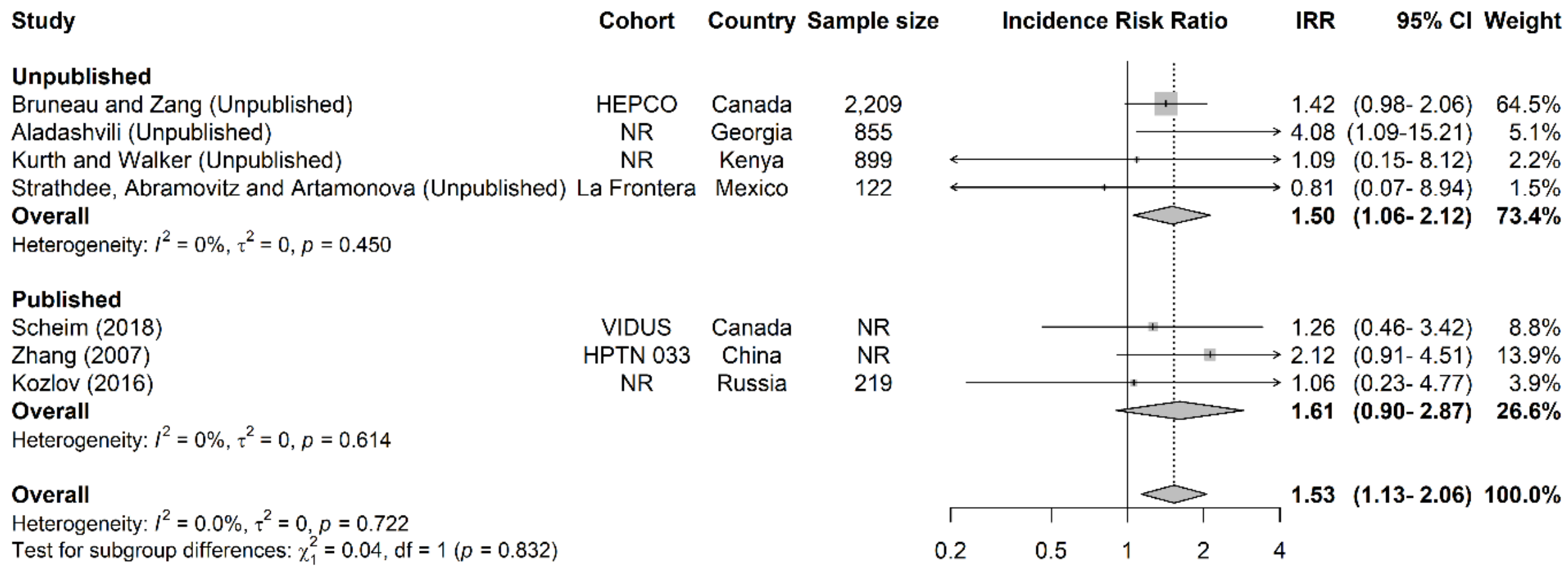

Meta-analysis of studies reporting the crude pooled association between lifetime exposure to sex work and HIV acquisition risk among female PWID

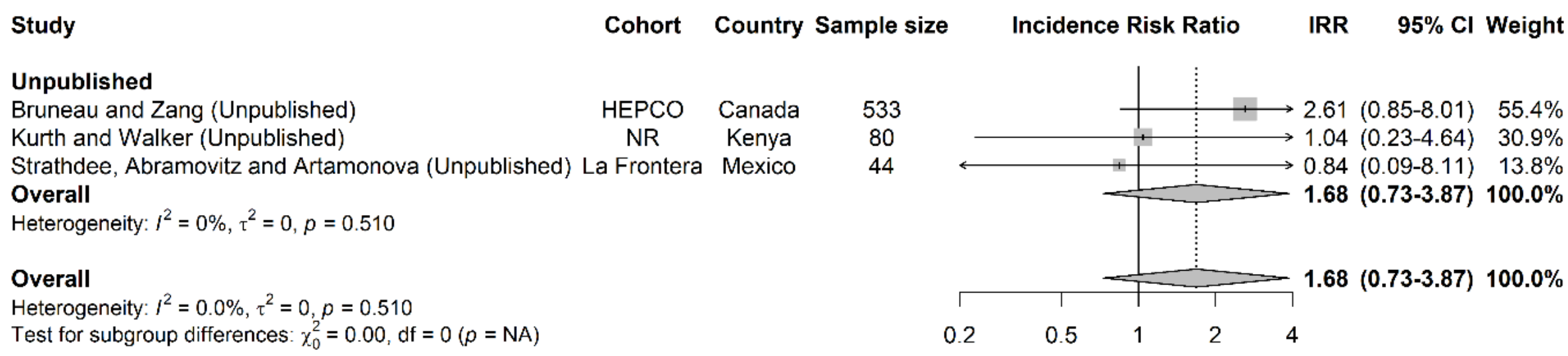

Meta-analysis of studies reporting the crude pooled association between lifetime exposure to sex work and HCV acquisition risk among male PWID

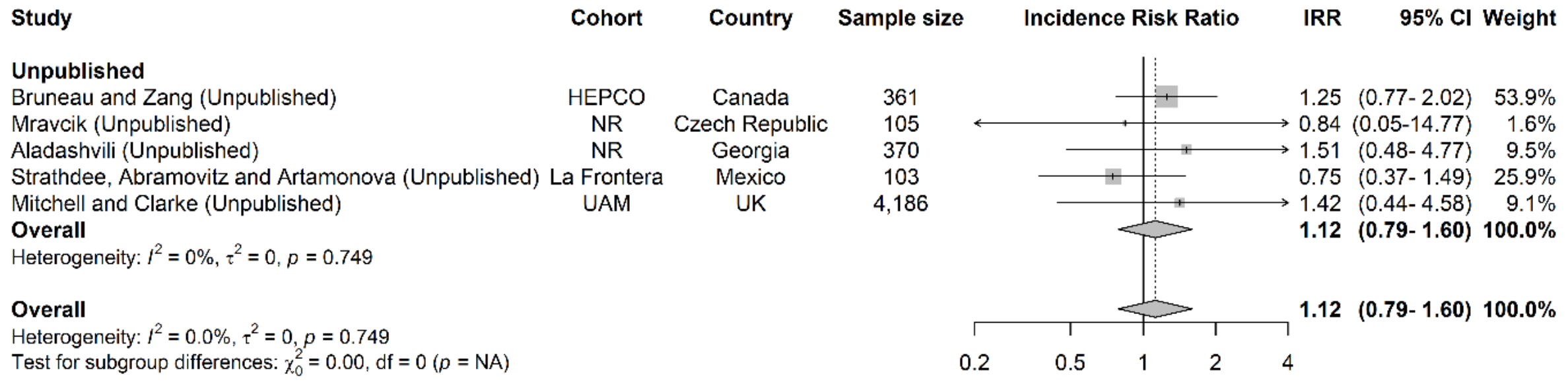

Meta-analysis of studies reporting the crude pooled association between lifetime exposure to sex work and HCV acquisition risk among female PWID

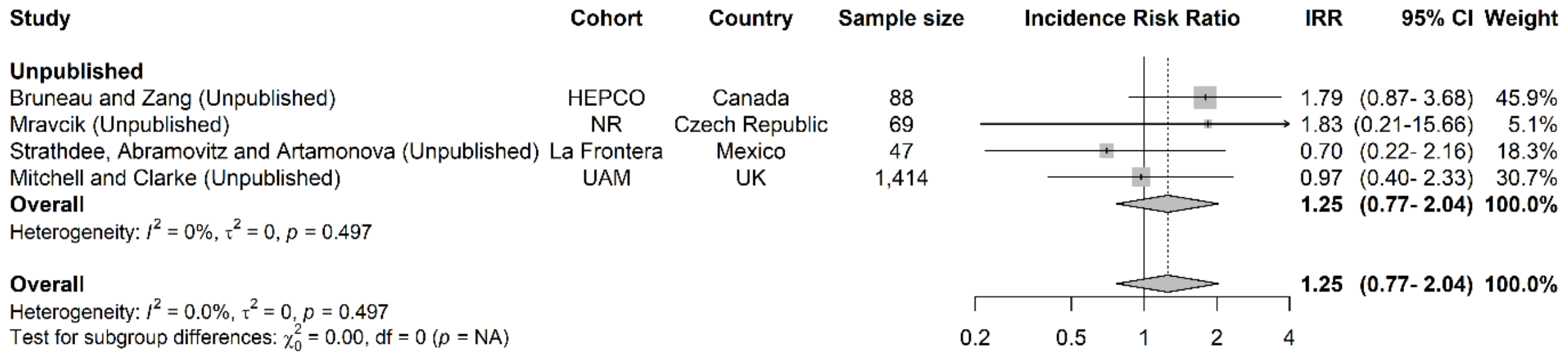

Meta-analysis of studies reporting the adjusted pooled association between lifetime exposure to sex work and HIV acquisition risk among male and female PWID

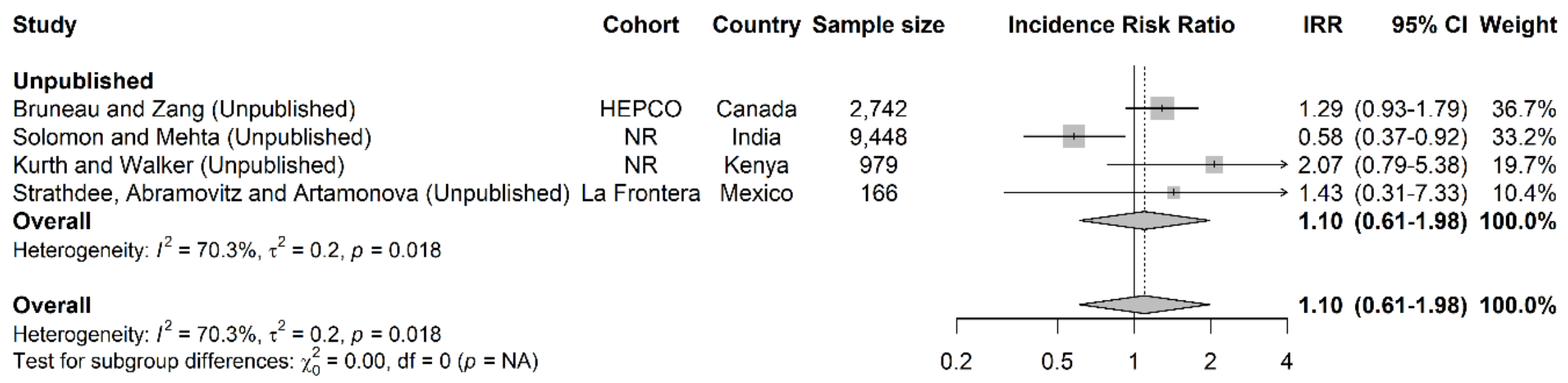

Meta-analysis of studies reporting the adjusted pooled association between lifetime exposure to sex work and HCV acquisition risk among male and female PWID

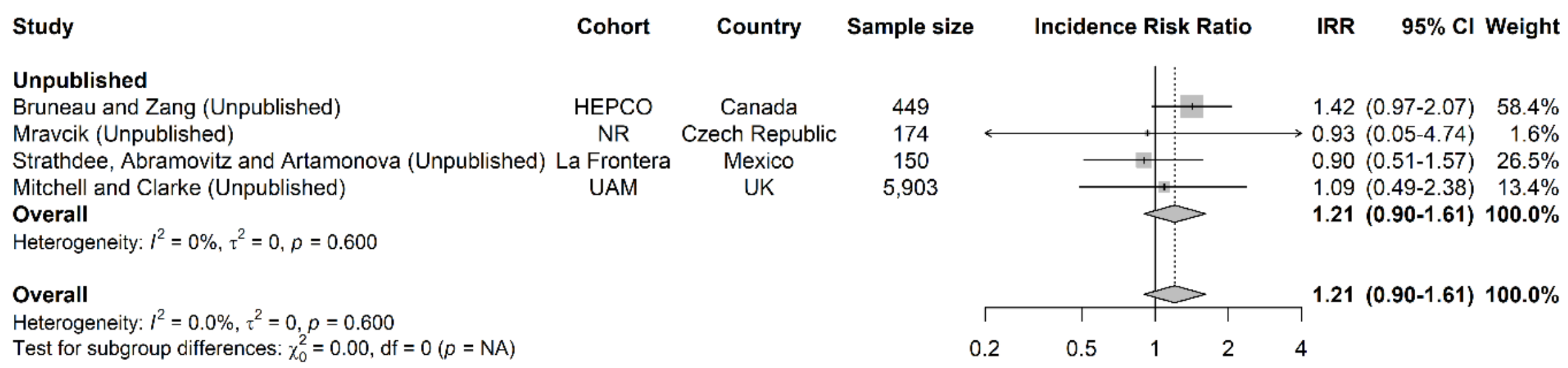

Meta-analysis of studies reporting the adjusted pooled association between lifetime exposure to sex work and HIV acquisition risk among male PWID

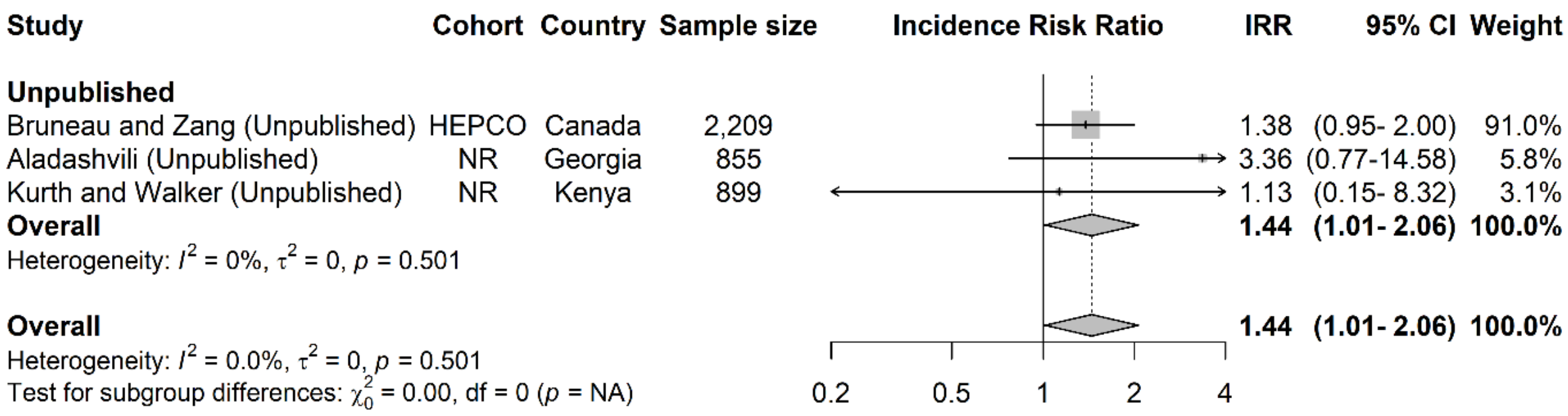

Meta-analysis of studies reporting the adjusted pooled association between lifetime exposure to sex work and HIV acquisition risk among female PWID

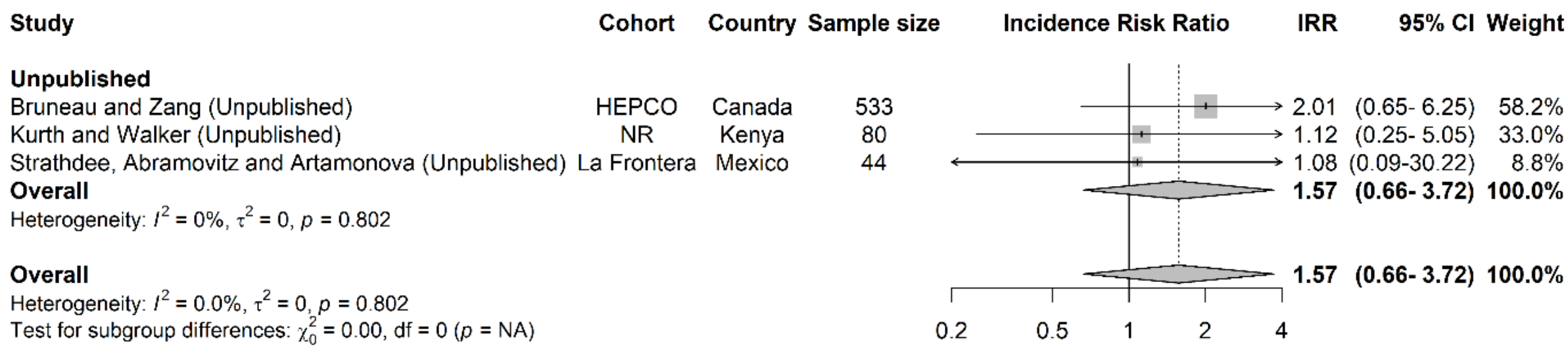

Meta-analysis of studies reporting the adjusted pooled association between lifetime exposure to sex work and HCV acquisition risk among male PWID

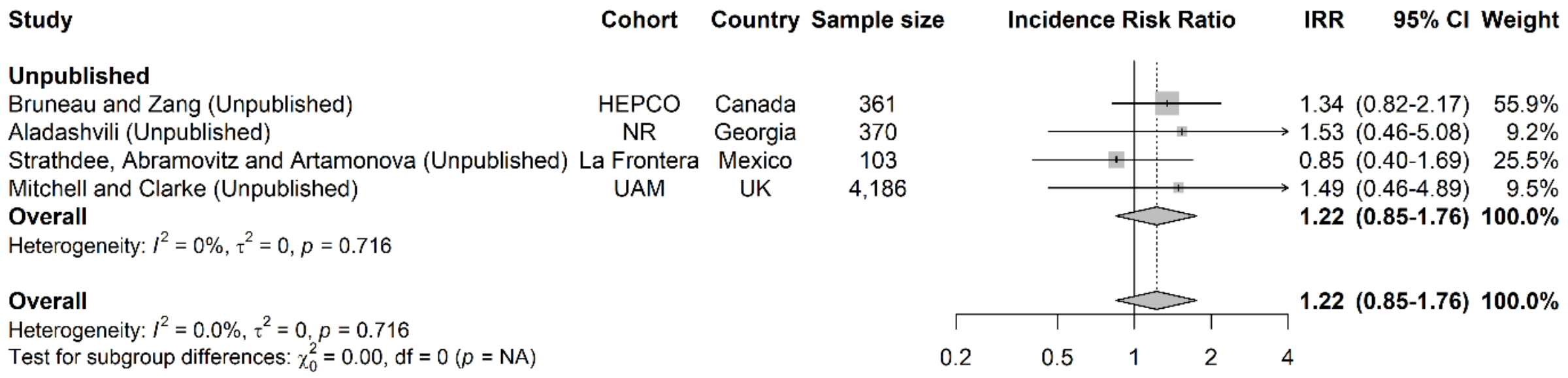

Meta-analysis of studies reporting the adjusted pooled association between lifetime exposure to sex work and HCV acquisition risk among female PWID

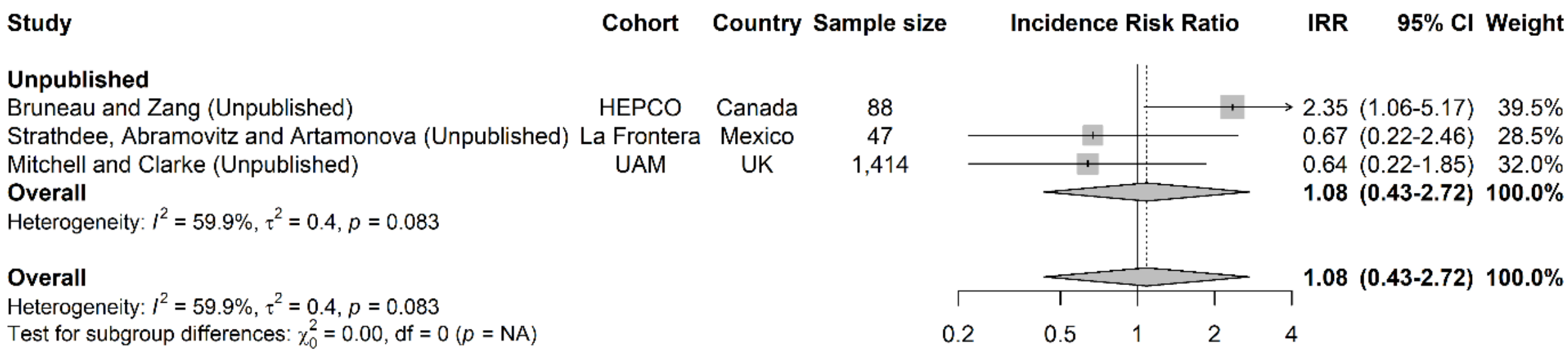

Meta-analysis of studies reporting the crude pooled association between lifetime exposure to male-with-male sex and HIV acquisition risk among male PWID

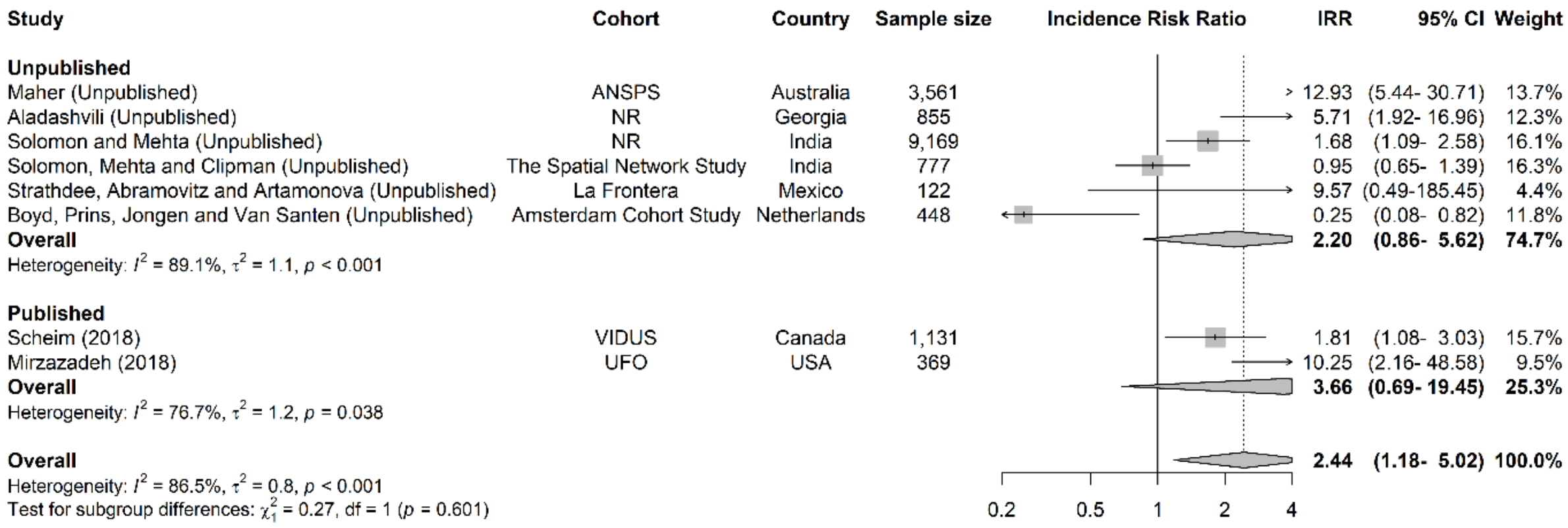

Meta-analysis of studies reporting the crude pooled association between lifetime exposure to male-with-male sex and HCV acquisition risk among male PWID

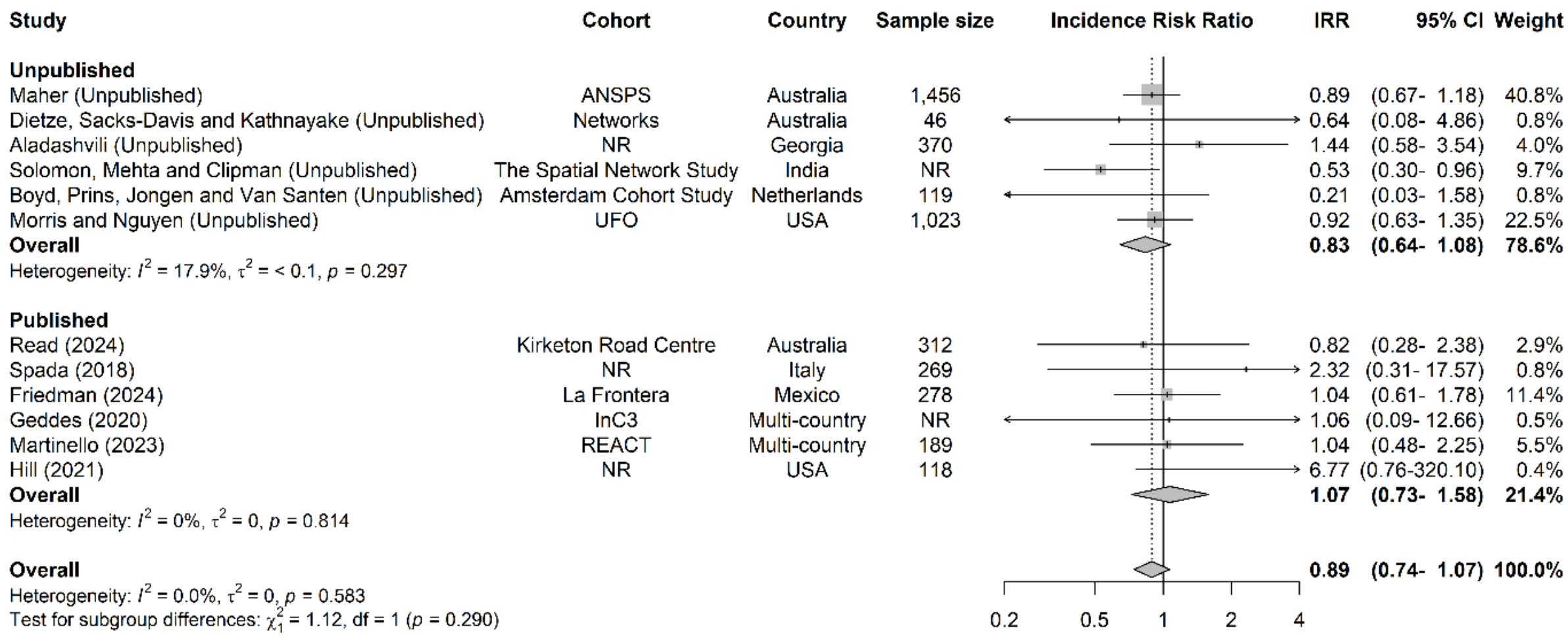

Meta-analysis of studies reporting the adjusted pooled association between lifetime exposure to male-with-male sex and HIV acquisition risk among male PWID

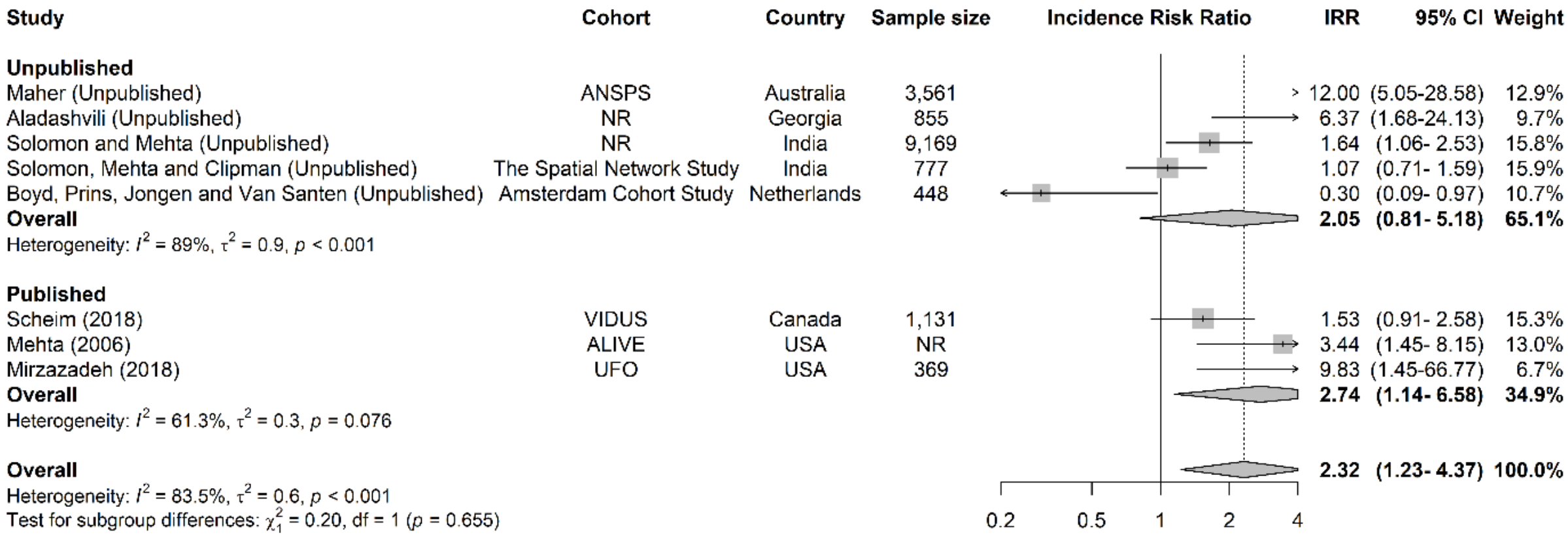

Meta-analysis of studies reporting the adjusted pooled association between lifetime exposure to male-with-male sex and HCV acquisition risk among male PWID

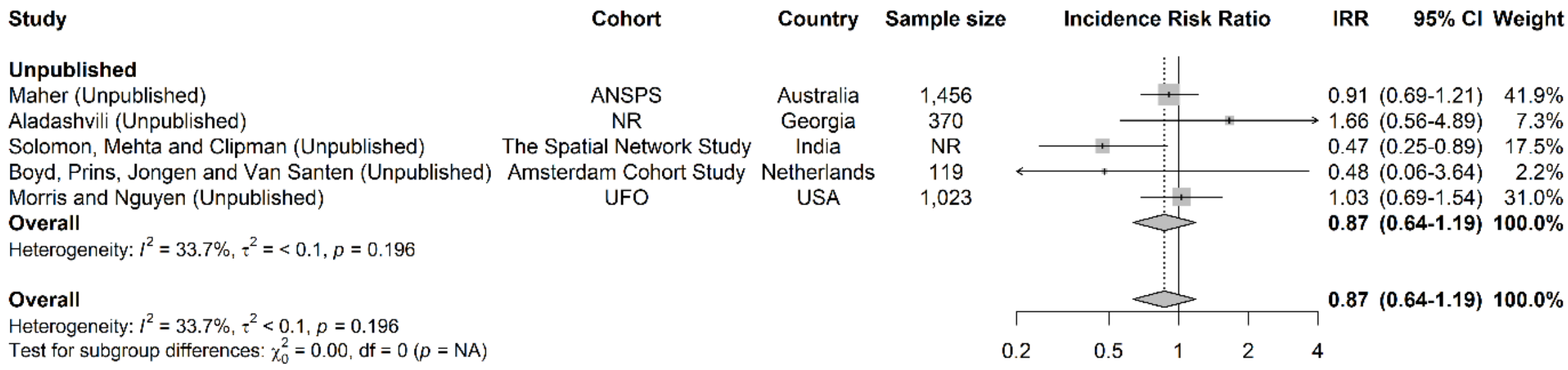

Appendix 11. Subgroup analyses of recent exposure to sex work or male-with-male sexual activity and HIV or HCV acquisition

Subgroup analysis of recent exposure to sex work and HIV acquisition risk among male and female PWID, by incidence estimation method

Study	Country	Sample size	IRR	95% CI	Weight
**Low**					
Blouin (2024)	Canada	15,524	2.50	(1.45- 4.31)	7.7%
Boyd, Prins, Jongen and Van Santen (Unpublished)	Netherlands	730	2.34	(1.47- 3.71)	8.3%
Bruneau and Zang (Unpublished)	Canada	2,738	1.36	(0.89- 2.10)	8.6%
DeBeck (b) (Unpublished)	Canada	1,951	3.92	(2.11- 7.28)	7.1%
Dumchev (Unpublished)	Ukraine	1,966	0.44	(0.03- 7.10)	1.0%
Jain (2020)	Mexico	651	2.42	(1.33- 4.41)	7.2%
Kral (2001)	USA	1,192	3.89	(2.33- 6.50)	7.9%
Maher (Unpublished)	Australia	5,650	2.15	(0.74- 6.23)	4.2%
Mirzazadeh (2018)	USA	564	1.76	(0.04-13.16)	0.9%
Skaathun (2024)	USA and Mexico	612	1.64	(0.44- 6.12)	3.2%
**Overall**			**2.38**	**(1.80- 3.16)**	**56.2%**
Heterogeneity: I^2 = 37.6%, τ^2 = < 0.1, p = 0.108					
**High**					
Booth (2016)	Ukraine	1,197	0.88	(0.32- 2.43)	4.5%
Kozlov (2006)	Russia	520	2.63	(0.83- 7.38)	4.1%
Kurth and Walker (Unpublished)	Kenya	979	2.51	(0.88- 7.19)	4.3%
Roy (2011)	Canada	2,249	1.31	(0.92- 1.86)	9.2%
Strathdee (2001)	USA	NR	1.11	(0.82- 1.47)	9.6%
Sypsa and Roussos (b) (Unpublished)	Greece	1,097	1.81	(0.43- 7.66)	2.8%
Trickey and Des Jarlais (Unpublished)	Vietnam	266	0.39	(0.02- 6.41)	0.9%
Yin (2006)	China	252	0.37	(0.09-23.30)	0.9%
**Overall**			**1.24**	**(1.01- 1.52)**	**36.4%**
Heterogeneity: I^2 = 0%, τ^2 = 0, p = 0.511					
**Missing**					
Kulchynska (2026)	Ukraine	19,882	1.20	(0.13-10.81)	1.4%
Solomon and Mehta (Unpublished)	India	9,448	0.44	(0.20- 0.95)	6.0%
**Overall**			**0.49**	**(0.24- 1.01)**	**7.4%**
Heterogeneity: I^2 = 0%, τ^2 = 0, p = 0.400					
**Overall**			**1.72**	**(1.30- 2.28)**	**100.0%**
Heterogeneity: I^2 = 62.9%, τ^2 = 0.2, p < 0.001					
Test for subgroup differences: χ^2_2 = 22.73, df = 2 (p < 0.001)					

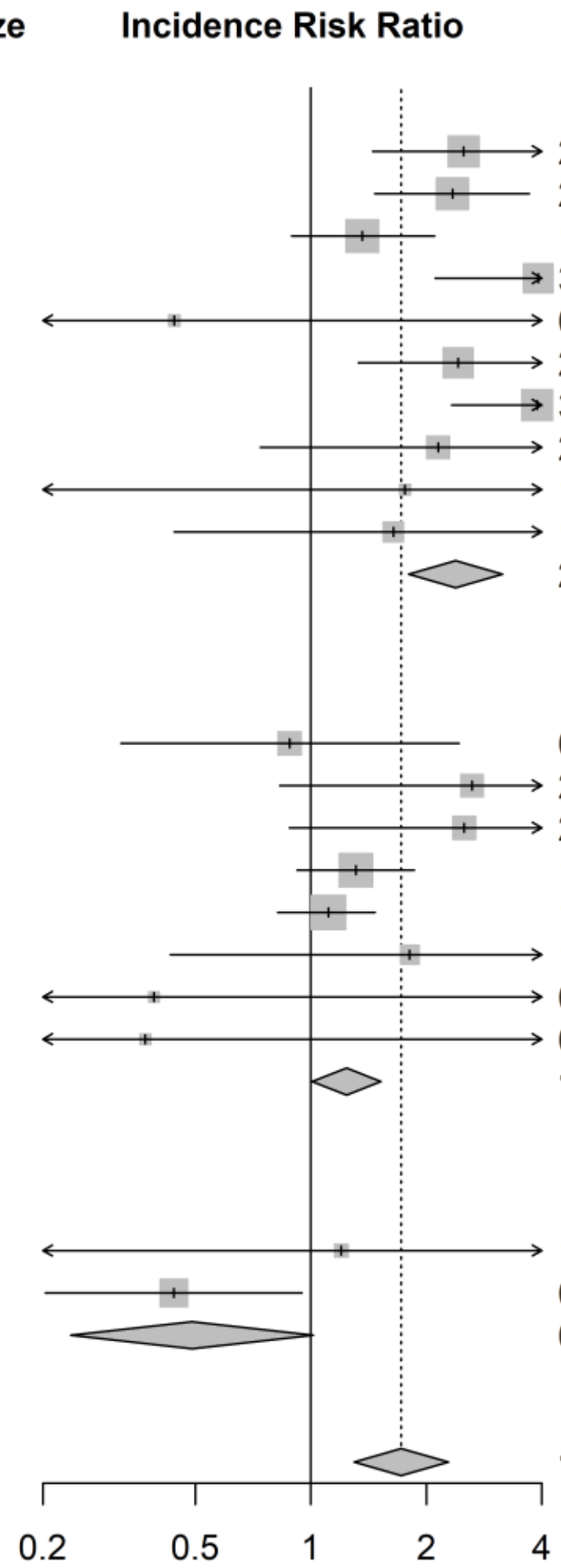

Subgroup analysis of recent exposure to sex work and HIV acquisition risk among male and female PWID, by median age of participants

Study	Country	Sample size	IRR	95% CI	Weight
**34+**					
Blouin (2024)	Canada	15,524	2.50	(1.45- 4.31)	8.5%
Bruneau and Zang (Unpublished)	Canada	2,738	1.36	(0.89- 2.10)	9.3%
Dumchev (Unpublished)	Ukraine	1,966	0.44	(0.03- 7.10)	1.2%
Jain (2020)	Mexico	651	2.42	(1.33- 4.41)	8.1%
Kral (2001)	USA	1,192	3.89	(2.33- 6.50)	8.7%
Skaathun (2024)	USA and Mexico	612	1.64	(0.44- 6.12)	3.9%
Strathdee (2001)	USA	NR	1.11	(0.82- 1.47)	10.2%
Sypsa and Roussos (b) (Unpublished)	Greece	1,097	1.81	(0.43- 7.66)	3.5%
Trickey and Des Jarlais (Unpublished)	Vietnam	266	0.39	(0.02- 6.41)	1.1%
**Overall**			**1.83**	**(1.21- 2.74)**	**54.6%**
Heterogeneity: I^2 = 67%, τ^2 = 0.2, p = 0.002					
**<34**					
Booth (2016)	Ukraine	1,197	0.88	(0.32- 2.43)	5.3%
Boyd, Prins, Jongen and Van Santen (Unpublished)	Netherlands	730	2.34	(1.47- 3.71)	9.1%
DeBeck (b) (Unpublished)	Canada	1,951	3.92	(2.11- 7.28)	7.9%
Kozlov (2006)	Russia	520	2.63	(0.83- 7.38)	4.9%
Kurth and Walker (Unpublished)	Kenya	979	2.51	(0.88- 7.19)	5.1%
Maher (Unpublished)	Australia	5,650	2.15	(0.74- 6.23)	5.0%
Solomon and Mehta (Unpublished)	India	9,448	0.44	(0.20- 0.95)	6.8%
Yin (2006)	China	252	0.37	(0.09-23.30)	1.2%
**Overall**			**1.67**	**(0.93- 2.99)**	**45.4%**
Heterogeneity: I^2 = 71%, τ^2 = 0.4, p = 0.001					
**Overall**			**1.77**	**(1.28- 2.44)**	**100.0%**
Heterogeneity: I^2 = 67.5%, τ^2 = 0.2, p < 0.001					
Test for subgroup differences: χ^2_1 = 0.06, df = 1 (p = 0.804)					

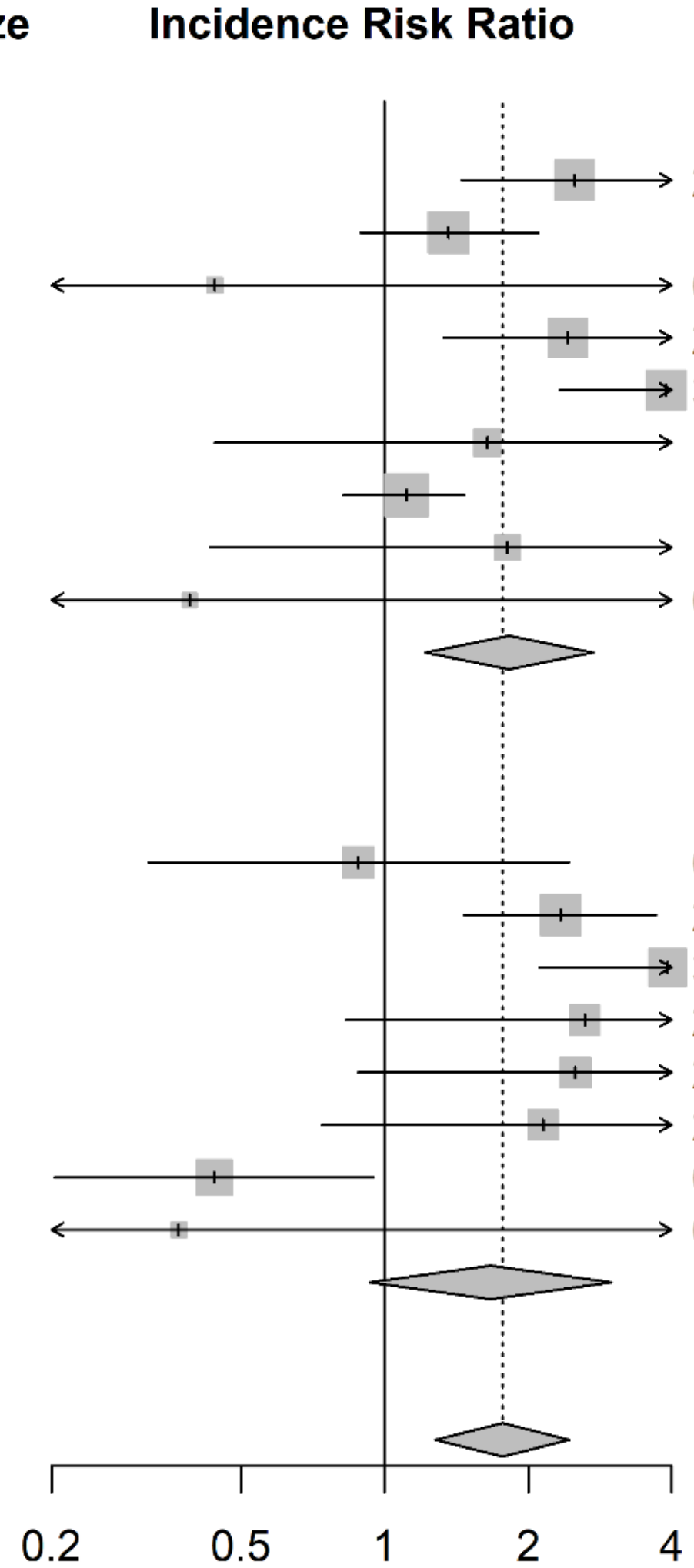

Subgroup analysis of recent exposure to sex work and HIV acquisition risk among male and female PWID, by median proportion recently incarcerated

Study	Country	Sample size	IRR	95% CI	Weight
**<0.1**					
Dumchev (Unpublished)	Ukraine	1,966	0.44	(0.03- 7.10)	3.4%
Kulchynska (2026)	Ukraine	19,882	1.20	(0.13-10.81)	4.9%
Solomon and Mehta (Unpublished)	India	9,448	0.44	(0.20- 0.95)	15.6%
Sypsa and Roussos (b) (Unpublished)	Greece	1,097	1.81	(0.43- 7.66)	8.9%
**Overall**			**0.66**	**(0.32- 1.36)**	**32.7%**
Heterogeneity: $I^2 = 9.1\%$, $\tau^2 = < 0.1$, $p = 0.348$					
**0.1+**					
Blouin (2024)	Canada	15,524	2.50	(1.45- 4.31)	18.3%
Boyd, Prins, Jongen and Van Santen (Unpublished)	Netherlands	730	2.34	(1.47- 3.71)	19.3%
DeBeck (b) (Unpublished)	Canada	1,951	3.92	(2.11- 7.28)	17.4%
Maher (Unpublished)	Australia	5,650	2.15	(0.74- 6.23)	12.2%
**Overall**			**2.66**	**(1.98- 3.58)**	**67.3%**
Heterogeneity: $I^2 = 0\%$, $\tau^2 = 0$, $p = 0.572$					
**Overall**			**1.77**	**(1.02- 3.07)**	**100.0%**
Heterogeneity: $I^2 = 68.0\%$, $\tau^2 = 0.4$, $p = 0.003$					
Test for subgroup differences: $\chi^2_1 = 12.41$, df = 1 ($p < 0.001$)					

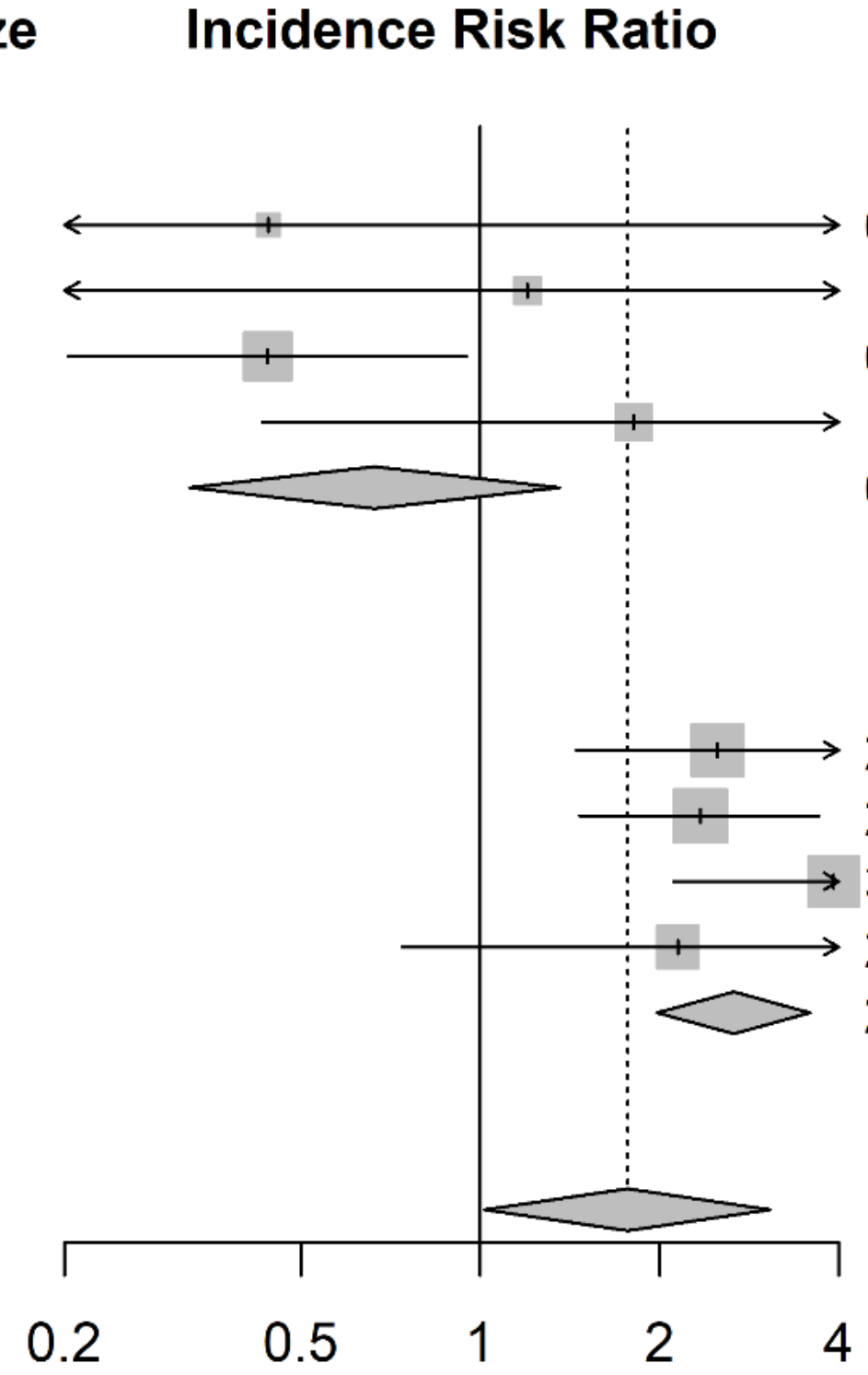

Subgroup analysis of recent exposure to sex work and HIV acquisition risk among male and female PWID, by median proportion recent or current OAT receipt

Study	Country	Sample size	IRR	95% CI	Weight
**<0.2**					
Bruneau and Zang (Unpublished)	Canada	2,738	1.36	(0.89- 2.10)	14.5%
Dumchev (Unpublished)	Ukraine	1,966	0.44	(0.03- 7.10)	2.4%
Kral (2001)	USA	1,192	3.89	(2.33- 6.50)	13.7%
Kulchynska (2026)	Ukraine	19,882	1.20	(0.13-10.81)	3.3%
Kurth and Walker (Unpublished)	Kenya	979	2.51	(0.88- 7.19)	8.8%
Trickey and Des Jarlais (Unpublished)	Vietnam	266	0.39	(0.02- 6.41)	2.1%
**Overall**			**1.87**	**(0.97- 3.63)**	**44.9%**
Heterogeneity: $I^2 = 59.5\%$, $\tau^2 = 0.3$, $p = 0.030$					
**0.2+**					
Boyd, Prins, Jongen and Van Santen (Unpublished)	Netherlands	730	2.34	(1.47- 3.71)	14.2%
DeBeck (b) (Unpublished)	Canada	1,951	3.92	(2.11- 7.28)	12.7%
Maher (Unpublished)	Australia	5,650	2.15	(0.74- 6.23)	8.7%
Mirzazadeh (2018)	USA	564	1.76	(0.04-13.16)	2.1%
Solomon and Mehta (Unpublished)	India	9,448	0.44	(0.20- 0.95)	11.2%
Sypsa and Roussos (b) (Unpublished)	Greece	1,097	1.81	(0.43- 7.66)	6.2%
**Overall**			**1.76**	**(0.85- 3.64)**	**55.1%**
Heterogeneity: $I^2 = 74.9\%$, $\tau^2 = 0.5$, $p = 0.001$					
**Overall**			**1.81**	**(1.15- 2.85)**	**100.0%**
Heterogeneity: $I^2 = 65.9\%$, $\tau^2 = 0.3$, $p < 0.001$					
Test for subgroup differences: $\chi^2_1 = 0.02$, df = 1 ($p = 0.901$)					

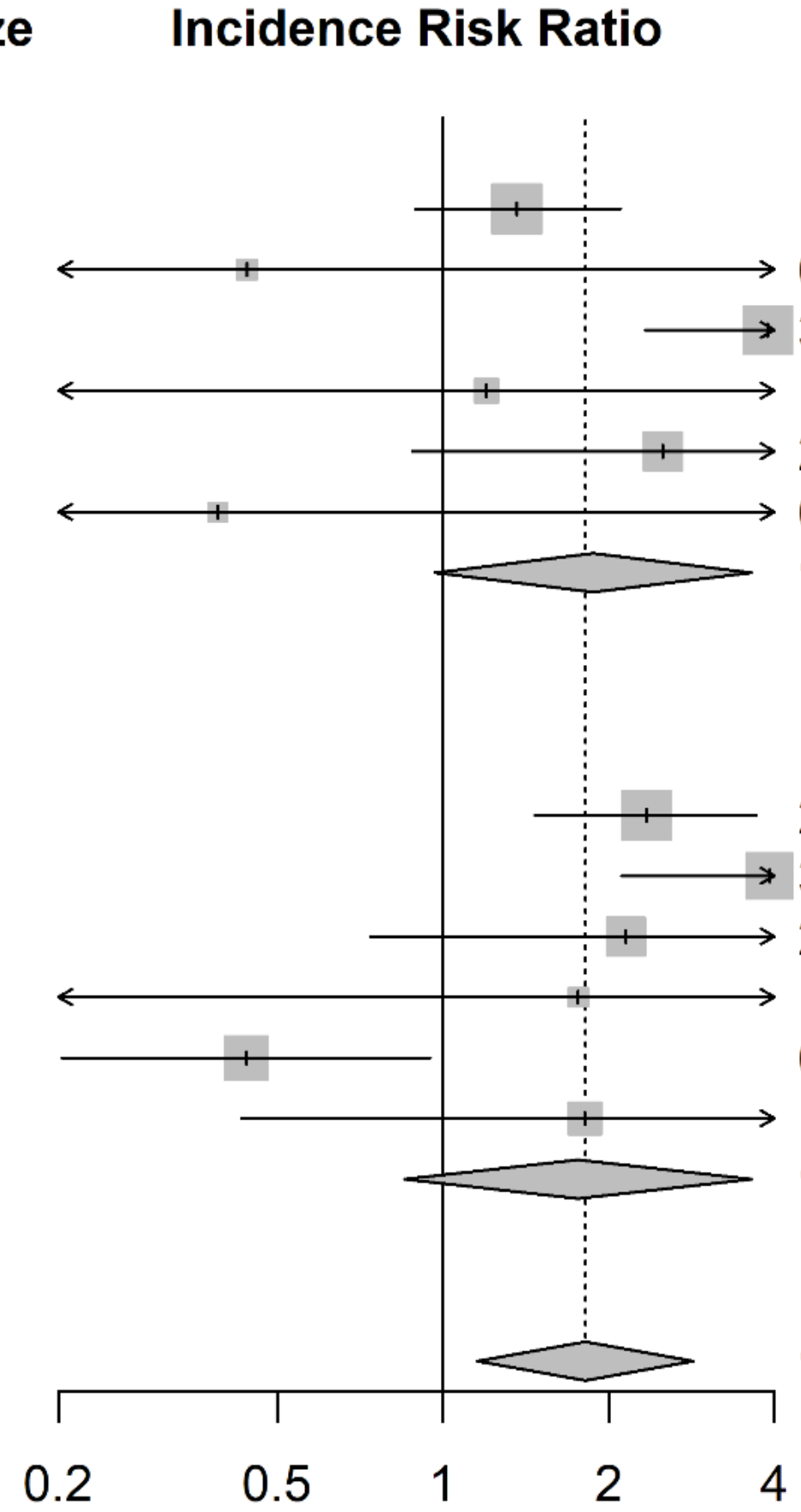

Subgroup analysis of recent exposure to sex work and HIV acquisition risk among male and female PWID, by median proportion recently homeless

Study	Country	Sample size	IRR	95% CI	Weight
**0.3+**					
Blouin (2024)	Canada	15,524	2.50	(1.45- 4.31)	15.9%
Bruneau and Zang (Unpublished)	Canada	2,738	1.36	(0.89- 2.10)	16.9%
DeBeck (b) (Unpublished)	Canada	1,951	3.92	(2.11- 7.28)	15.2%
Mirzazadeh (2018)	USA	564	1.76	(0.04-13.16)	2.9%
**Overall**			**2.27**	**(1.29- 3.98)**	**51.0%**
Heterogeneity: $I^2 = 63.5\%$, $\tau^2 = 0.2$, $p = 0.042$					
**<0.3**					
Kral (2001)	USA	1,192	3.89	(2.33- 6.50)	16.2%
Kurth and Walker (Unpublished)	Kenya	979	2.51	(0.88- 7.19)	11.0%
Solomon and Mehta (Unpublished)	India	9,448	0.44	(0.20- 0.95)	13.7%
Sypsa and Roussos (b) (Unpublished)	Greece	1,097	1.81	(0.43- 7.66)	8.1%
**Overall**			**1.67**	**(0.53- 5.30)**	**49.0%**
Heterogeneity: $I^2 = 86.1\%$, $\tau^2 = 1.1$, $p < 0.001$					
**Overall**			**1.97**	**(1.15- 3.38)**	**100.0%**
Heterogeneity: $I^2 = 76.5\%$, $\tau^2 = 0.4$, $p < 0.001$					
Test for subgroup differences: $\chi^2_1 = 0.22$, df = 1 ($p = 0.642$)					

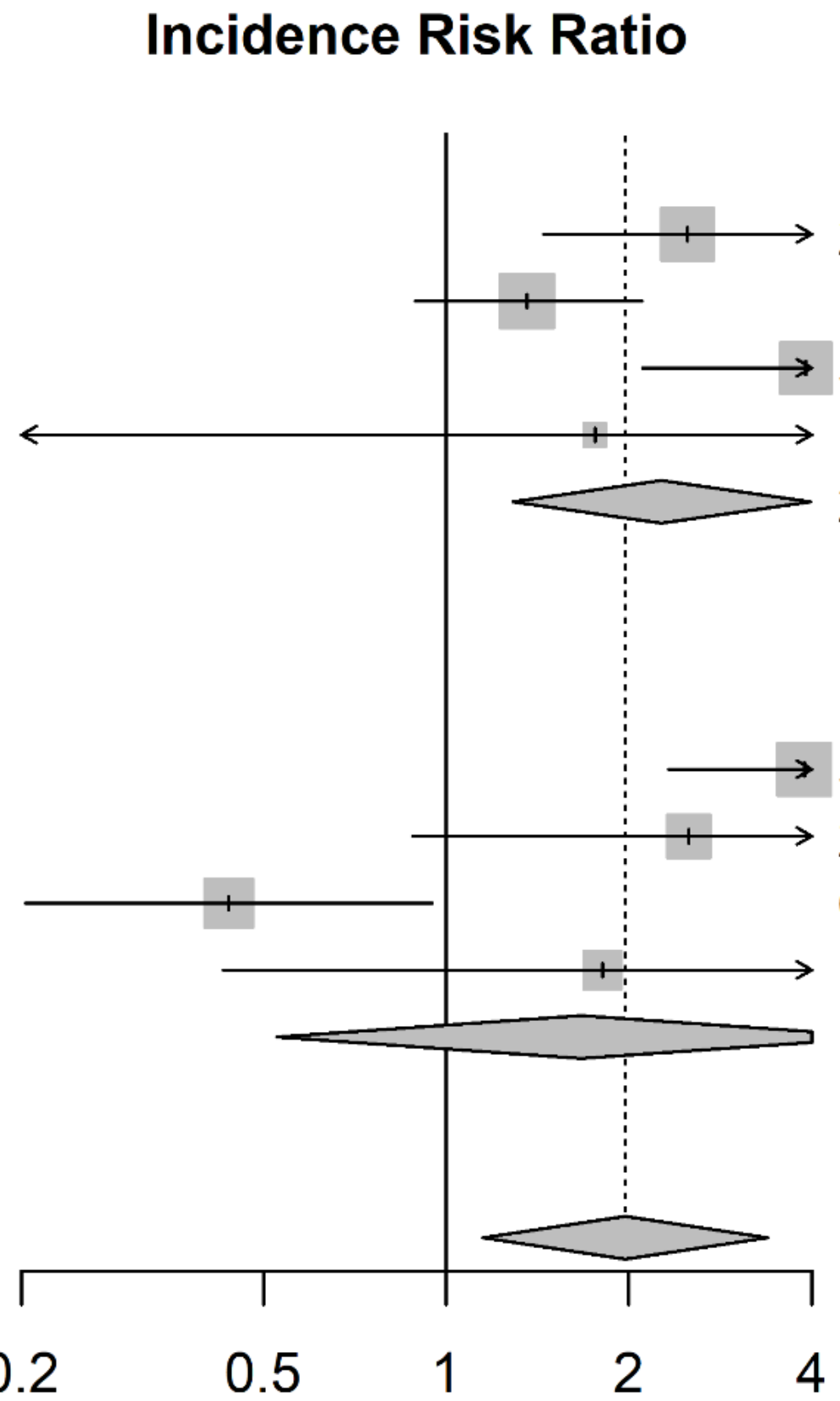

Subgroup analysis of recent exposure to sex work and HIV acquisition risk among male and female PWID, by median proportion female

Study	Country	Sample size	IRR	95% CI	Weight
**<0.3**					
Blouin (2024)	Canada	15,524	2.50	(1.45- 4.31)	7.9%
Booth (2016)	Ukraine	1,197	0.88	(0.32- 2.43)	4.7%
Bruneau and Zang (Unpublished)	Canada	2,738	1.36	(0.89- 2.10)	8.8%
Kulchynska (2026)	Ukraine	19,882	1.20	(0.13-10.81)	1.5%
Kurth and Walker (Unpublished)	Kenya	979	2.51	(0.88- 7.19)	4.5%
Solomon and Mehta (Unpublished)	India	9,448	0.44	(0.20- 0.95)	6.2%
Strathdee (2001)	USA	NR	1.11	(0.82- 1.47)	9.8%
Trickey and Des Jarlais (Unpublished)	Vietnam	266	0.39	(0.02- 6.41)	0.9%
Yin (2006)	China	252	0.37	(0.09-23.30)	1.0%
**Overall**			**1.20**	**(0.81- 1.78)**	**45.2%**
Heterogeneity: $I^2 = 54.5\%$, $\tau^2 = 0.1$, $p = 0.025$					
**0.3+**					
Boyd, Prins, Jongen and Van Santen (Unpublished)	Netherlands	730	2.34	(1.47- 3.71)	8.5%
DeBeck (b) (Unpublished)	Canada	1,951	3.92	(2.11- 7.28)	7.3%
Dumchev (Unpublished)	Ukraine	1,966	0.44	(0.03- 7.10)	1.0%
Jain (2020)	Mexico	651	2.42	(1.33- 4.41)	7.4%
Kozlov (2006)	Russia	520	2.63	(0.83- 7.38)	4.3%
Kral (2001)	USA	1,192	3.89	(2.33- 6.50)	8.1%
Maher (Unpublished)	Australia	5,650	2.15	(0.74- 6.23)	4.4%
Mirzazadeh (2018)	USA	564	1.76	(0.04-13.16)	0.9%
Roy (2011)	Canada	2,249	1.31	(0.92- 1.86)	9.4%
Skaathun (2024)	USA and Mexico	612	1.64	(0.44- 6.12)	3.4%
**Overall**			**2.33**	**(1.67- 3.27)**	**54.8%**
Heterogeneity: $I^2 = 50.6\%$, $\tau^2 = 0.1$, $p = 0.033$					
**Overall**			**1.72**	**(1.28- 2.29)**	**100.0%**
Heterogeneity: $I^2 = 64.8\%$, $\tau^2 = 0.2$, $p < 0.001$					
Test for subgroup differences: $\chi^2_1 = 6.36$, df = 1 ($p = 0.012$)					

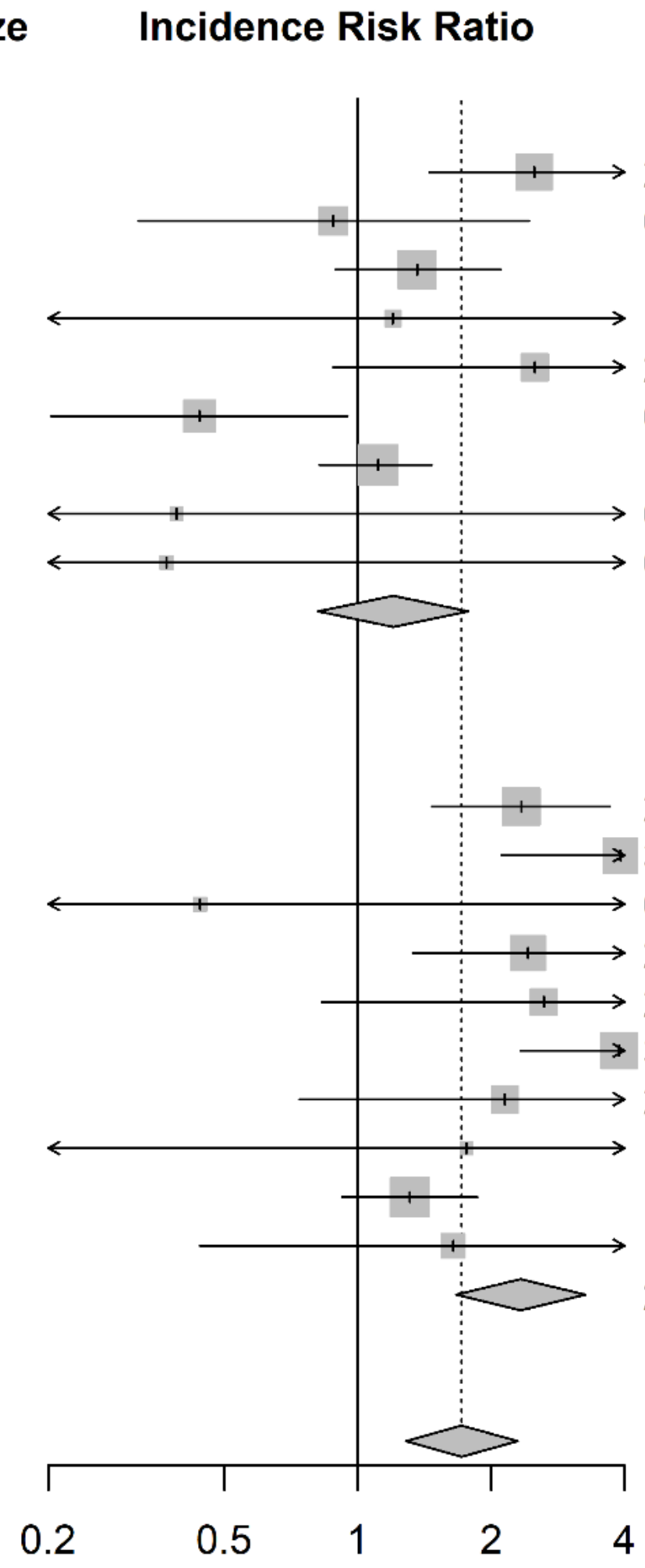

Subgroup analysis of recent exposure to sex work and HCV acquisition risk among male and female PWID, by incidence estimation method

Study	Country	Sample size	IRR	95% CI	Weight
**Direct**					
Bach (2016)	Canada	440	3.41	(2.33- 5.00)	7.3%
Blouin and Hankins (Unpublished)	Canada	926	1.60	(1.23- 2.09)	8.4%
Boyd, Prins, Jongen and Van Santen (Unpublished)	Netherlands	178	1.69	(0.88- 3.25)	5.0%
Bruneau and Zang (Unpublished)	Canada	449	2.14	(1.32- 3.48)	6.4%
DeBeck (a) (Unpublished)	Canada	NR	0.84	(0.45- 1.56)	5.2%
Dietze, Sacks-Davis and Kathnayake (Unpublished)	Australia	134	5.22	(0.32-84.04)	0.5%
Dumchev (Unpublished)	Ukraine	1,237	0.31	(0.10- 0.96)	2.5%
Hagan (2004)	USA	426	2.90	(1.90- 4.60)	6.8%
Hahn (2002)	USA	195	2.30	(1.10- 4.70)	4.5%
Havens and Young (Unpublished)	USA	277	0.75	(0.28- 2.05)	3.0%
Maher (Unpublished)	Australia	5,650	1.36	(1.05- 1.77)	8.5%
Moles (2020)	Vietnam	92	0.83	(0.22- 3.18)	1.9%
Morris and Nguyen (Unpublished)	USA	1,227	1.07	(0.66- 1.73)	6.4%
Roy (2007)	Canada	359	2.31	(1.47- 3.63)	6.7%
Spittal (2012)	Canada	148	1.71	(1.16- 2.53)	7.3%
Strathdee, Abramovitz and Artamonova (Unpublished)	Mexico	150	0.92	(0.38- 1.90)	4.0%
Sypsa and Roussos (a) (Unpublished)	Greece	1,630	3.21	(0.19-55.22)	0.5%
Vallejo (2015)	Spain	57	1.90	(0.80- 4.60)	3.6%
**Overall**			**1.61**	**(1.28- 2.02)**	**88.4%**
Heterogeneity: $I^2 = 65.1\%$, $\tau^2 = 0.1$, $p < 0.001$					
**Indirect**					
Antuori (2021)	Spain	215	1.56	(0.60- 4.03)	3.2%
Mitchell and Clarke (Unpublished)	UK	5,600	1.07	(0.62- 1.85)	5.8%
Palmateer (Unpublished)	UK	1,007	1.09	(0.18-46.64)	0.5%
Platt and Hope (Unpublished)	UK	692	3.00	(0.82-10.99)	2.0%
**Overall**			**1.31**	**(0.84- 2.03)**	**11.6%**
Heterogeneity: $I^2 = 0\%$, $\tau^2 = 0$, $p = 0.524$					
**Overall**			**1.59**	**(1.29- 1.96)**	**100.0%**
Heterogeneity: $I^2 = 59.7\%$, $\tau^2 = 0.1$, $p < 0.001$					
Test for subgroup differences: $\chi^2_1 = 0.67$, df = 1 ($p = 0.413$)					

Incidence Risk Ratio

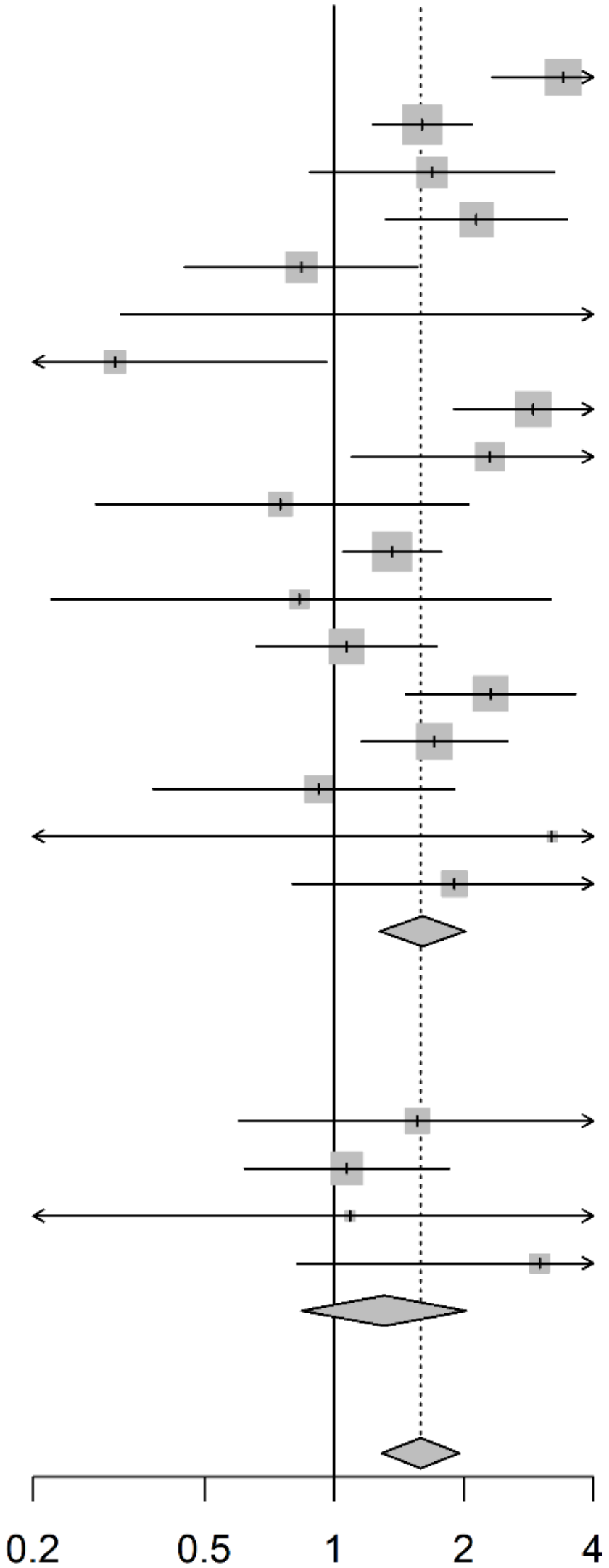

Subgroup analysis of recent exposure to sex work and HCV acquisition risk among male and female PWID, by median age of participants

Study	Country	Sample size	IRR	95% CI	Weight
**<31.8**					
Bach (2016)	Canada	440	3.41	(2.33- 5.00)	9.2%
Blouin and Hankins (Unpublished)	Canada	926	1.60	(1.23- 2.09)	10.6%
Boyd, Prins, Jongen and Van Santen (Unpublished)	Netherlands	178	1.69	(0.88- 3.25)	6.1%
Dietze, Sacks-Davis and Kathnayake (Unpublished)	Australia	134	5.22	(0.32-84.04)	0.6%
Hahn (2002)	USA	195	2.30	(1.10- 4.70)	5.5%
Morris and Nguyen (Unpublished)	USA	1,227	1.07	(0.66- 1.73)	7.9%
Platt and Hope (Unpublished)	UK	692	3.00	(0.82-10.99)	2.5%
Spittal (2012)	Canada	148	1.71	(1.16- 2.53)	9.1%
**Overall**			**1.90**	**(1.40- 2.58)**	**51.4%**
Heterogeneity: $I^2 = 60.2\%$, $\tau^2 = < 0.1$, $p = 0.014$					
**31.8+**					
Bruneau and Zang (Unpublished)	Canada	449	2.14	(1.32- 3.48)	7.9%
Dumchev (Unpublished)	Ukraine	1,237	0.31	(0.10- 0.96)	3.1%
Havens and Young (Unpublished)	USA	277	0.75	(0.28- 2.05)	3.7%
Maher (Unpublished)	Australia	5,650	1.36	(1.05- 1.77)	10.6%
Mitchell and Clarke (Unpublished)	UK	5,600	1.07	(0.62- 1.85)	7.2%
Moles (2020)	Vietnam	92	0.83	(0.22- 3.18)	2.4%
Roy (2007)	Canada	359	2.31	(1.47- 3.63)	8.3%
Strathdee, Abramovitz and Artamonova (Unpublished)	Mexico	150	0.92	(0.38- 1.90)	4.9%
Sypsa and Roussos (a) (Unpublished)	Greece	1,630	3.21	(0.19-55.22)	0.6%
**Overall**			**1.27**	**(0.90- 1.78)**	**48.6%**
Heterogeneity: $I^2 = 57.7\%$, $\tau^2 = 0.1$, $p = 0.015$					
**Overall**			**1.57**	**(1.25- 1.97)**	**100.0%**
Heterogeneity: $I^2 = 61.1\%$, $\tau^2 = 0.1$, $p < 0.001$					
Test for subgroup differences: $\chi^2_1 = 3.01$, df = 1 ($p = 0.083$)					

Incidence Risk Ratio

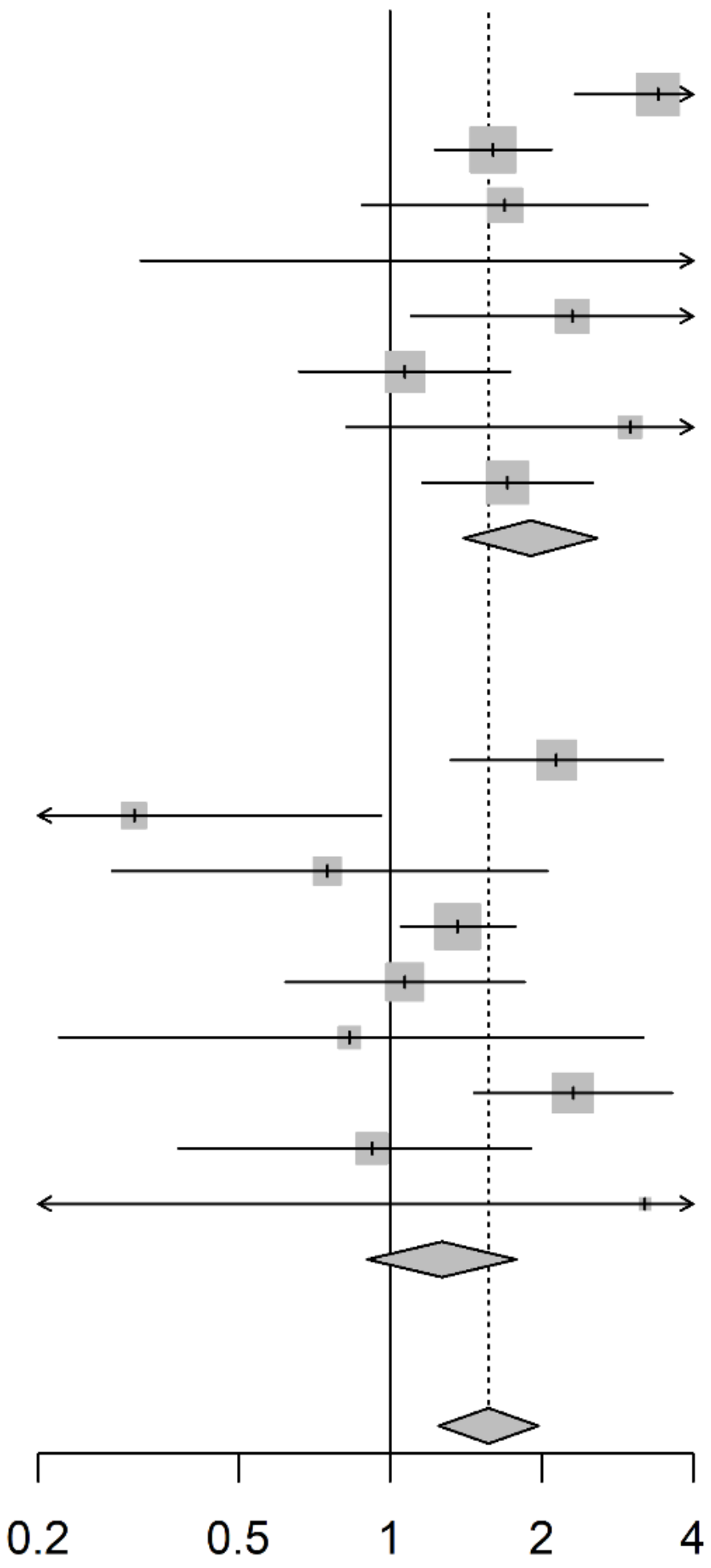

Subgroup analysis of recent exposure to sex work and HCV acquisition risk among male and female PWID, by median proportion recently incarcerated

Study	Country	Sample size	IRR	95% CI	Weight
**0.3+**					
Hahn (2002)	USA	195	2.30	(1.10- 4.70)	5.8%
Havens and Young (Unpublished)	USA	277	0.75	(0.28- 2.05)	3.5%
Morris and Nguyen (Unpublished)	USA	1,227	1.07	(0.66- 1.73)	10.2%
Platt and Hope (Unpublished)	UK	692	3.00	(0.82-10.99)	2.1%
Spittal (2012)	Canada	148	1.71	(1.16- 2.53)	12.9%
Strathdee, Abramovitz and Artamonova (Unpublished)	Mexico	150	0.92	(0.38- 1.90)	4.9%
Vallejo (2015)	Spain	57	1.90	(0.80- 4.60)	4.3%
**Overall**			**1.45**	**(1.07- 1.96)**	**43.8%**
Heterogeneity: I^2 = 27.3%, τ^2 = < 0.1, p = 0.220					
**<0.3**					
Blouin and Hankins (Unpublished)	Canada	926	1.60	(1.23- 2.09)	17.7%
Boyd, Prins, Jongen and Van Santen (Unpublished)	Netherlands	178	1.69	(0.88- 3.25)	6.8%
Bruneau and Zang (Unpublished)	Canada	449	2.14	(1.32- 3.48)	10.2%
Dietze, Sacks-Davis and Kathnayake (Unpublished)	Australia	134	5.22	(0.32-84.04)	0.5%
Dumchev (Unpublished)	Ukraine	1,237	0.31	(0.10- 0.96)	2.8%
Maher (Unpublished)	Australia	5,650	1.36	(1.05- 1.77)	17.8%
Sypsa and Roussos (a) (Unpublished)	Greece	1,630	3.21	(0.19-55.22)	0.5%
**Overall**			**1.51**	**(1.12- 2.02)**	**56.2%**
Heterogeneity: I^2 = 47.7%, τ^2 = < 0.1, p = 0.075					
**Overall**			**1.48**	**(1.22- 1.81)**	**100.0%**
Heterogeneity: I^2 = 34.3%, τ^2 < 0.1, p = 0.101					
Test for subgroup differences: χ^2_1 = 0.03, df = 1 (p = 0.854)					

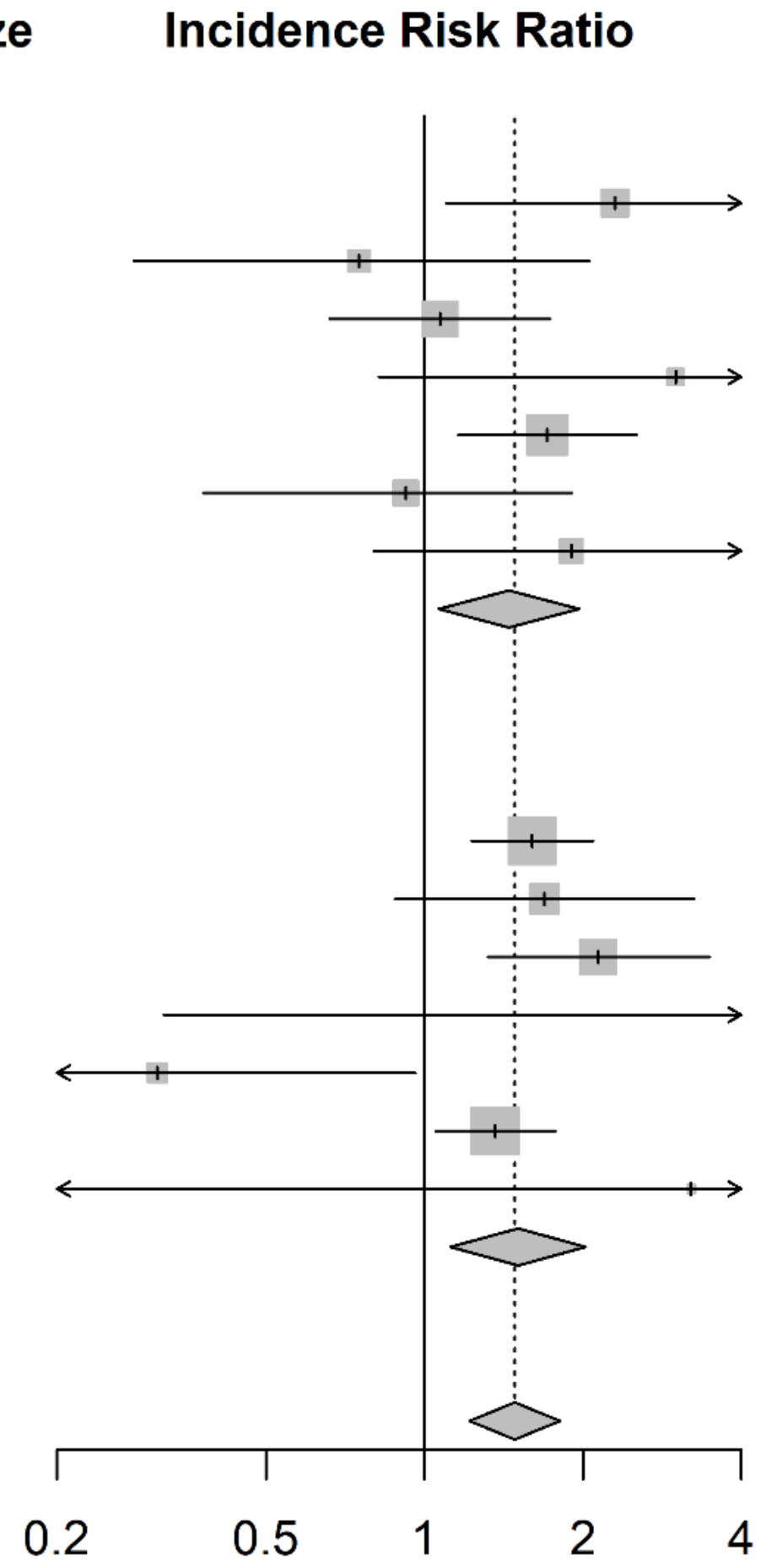

Subgroup analysis of recent exposure to sex work and HCV acquisition risk among male and female PWID, by median proportion recent or current OAT receipt

Study	Country	Sample size	IRR	95% CI	Weight
**<0.2**					
Bach (2016)	Canada	440	3.41	(2.33- 5.00)	10.3%
Blouin and Hankins (Unpublished)	Canada	926	1.60	(1.23- 2.09)	11.7%
Dumchev (Unpublished)	Ukraine	1,237	0.31	(0.10- 0.96)	3.6%
Moles (2020)	Vietnam	92	0.83	(0.22- 3.18)	2.8%
Morris and Nguyen (Unpublished)	USA	1,227	1.07	(0.66- 1.73)	9.0%
Spittal (2012)	Canada	148	1.71	(1.16- 2.53)	10.2%
Strathdee, Abramovitz and Artamonova (Unpublished)	Mexico	150	0.92	(0.38- 1.90)	5.7%
**Overall**			**1.35**	**(0.88- 2.07)**	**53.2%**
Heterogeneity: $I^2 = 78.9\%$, $\tau^2 = 0.2$, $p < 0.001$					
**0.2+**					
Boyd, Prins, Jongen and Van Santen (Unpublished)	Netherlands	178	1.69	(0.88- 3.25)	7.0%
Bruneau and Zang (Unpublished)	Canada	449	2.14	(1.32- 3.48)	9.0%
Dietze, Sacks-Davis and Kathnayake (Unpublished)	Australia	134	5.22	(0.32-84.04)	0.8%
Hagan (2004)	USA	426	2.90	(1.90- 4.60)	9.5%
Maher (Unpublished)	Australia	5,650	1.36	(1.05- 1.77)	11.7%
Platt and Hope (Unpublished)	UK	692	3.00	(0.82-10.99)	2.9%
Sypsa and Roussos (a) (Unpublished)	Greece	1,630	3.21	(0.19-55.22)	0.7%
Vallejo (2015)	Spain	57	1.90	(0.80- 4.60)	5.1%
**Overall**			**1.95**	**(1.47- 2.60)**	**46.8%**
Heterogeneity: $I^2 = 34.7\%$, $\tau^2 = < 0.1$, $p = 0.151$					
**Overall**			**1.68**	**(1.31- 2.16)**	**100.0%**
Heterogeneity: $I^2 = 64.5\%$, $\tau^2 = 0.1$, $p < 0.001$					
Test for subgroup differences: $\chi^2_1 = 1.98$, df = 1 ($p = 0.159$)					

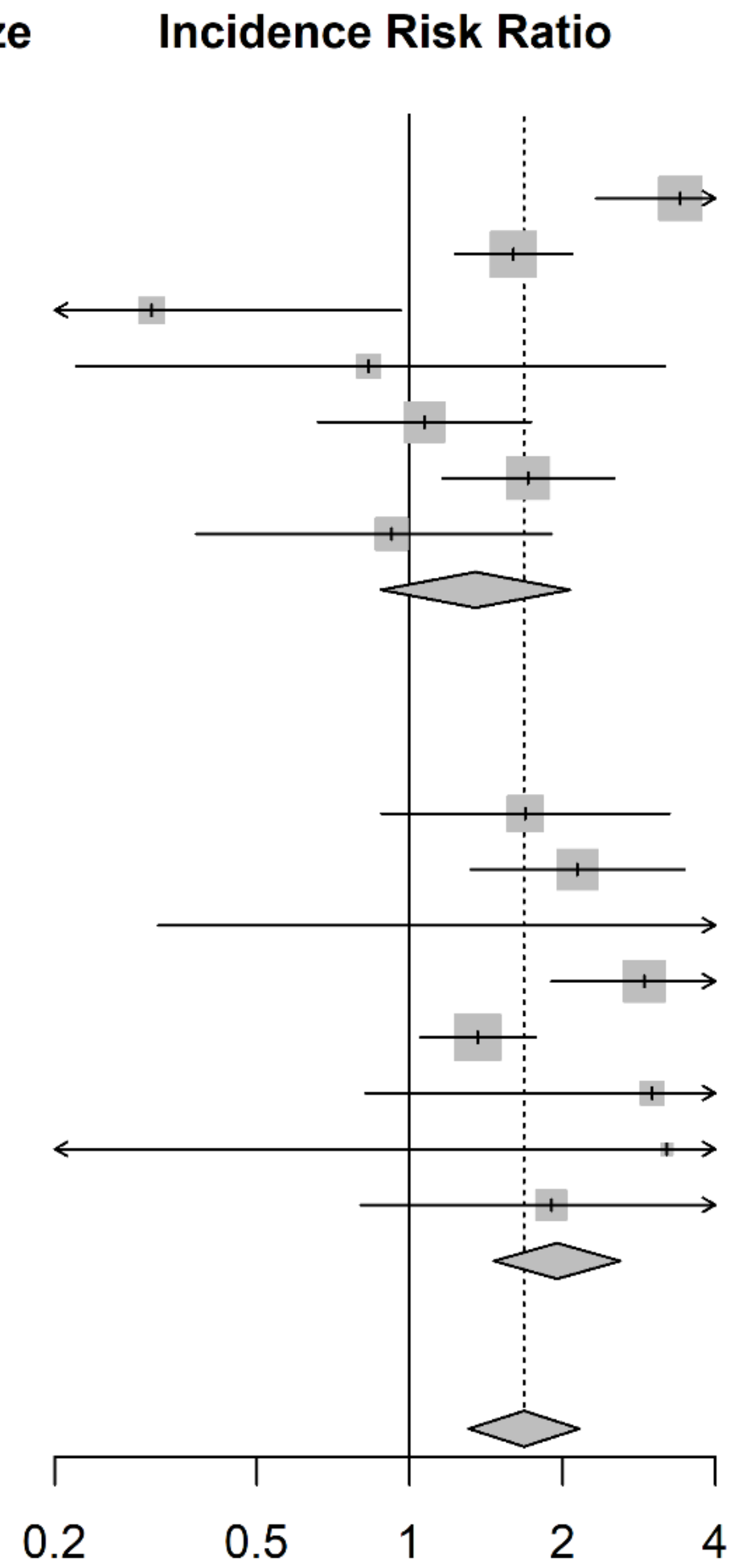

Subgroup analysis of recent exposure to sex work and HCV acquisition risk among male and female PWID, by median proportion recently homeless

Study	Country	Sample size	IRR	95% CI	Weight
**<0.4**					
Antuori (2021)	Spain	215	1.56	(0.60- 4.03)	6.6%
Bach (2016)	Canada	440	3.41	(2.33- 5.00)	16.0%
Dietze, Sacks-Davis and Kathnayake (Unpublished)	Australia	134	5.22	(0.32-84.04)	1.0%
Sypsa and Roussos (a) (Unpublished)	Greece	1,630	3.21	(0.19-55.22)	1.0%
Vallejo (2015)	Spain	57	1.90	(0.80- 4.60)	7.4%
**Overall**			**2.89**	**(2.09- 3.99)**	**32.1%**
Heterogeneity: $I^2 = 0\%$, $\tau^2 = 0$, $p = 0.494$					
**0.4+**					
Blouin and Hankins (Unpublished)	Canada	926	1.60	(1.23- 2.09)	18.7%
Bruneau and Zang (Unpublished)	Canada	449	2.14	(1.32- 3.48)	13.7%
Hahn (2002)	USA	195	2.30	(1.10- 4.70)	9.3%
Morris and Nguyen (Unpublished)	USA	1,227	1.07	(0.66- 1.73)	13.8%
Platt and Hope (Unpublished)	UK	692	3.00	(0.82-10.99)	4.1%
Strathdee, Abramovitz and Artamonova (Unpublished)	Mexico	150	0.92	(0.38- 1.90)	8.2%
**Overall**			**1.59**	**(1.20- 2.10)**	**67.9%**
Heterogeneity: $I^2 = 35.3\%$, $\tau^2 = < 0.1$, $p = 0.172$					
**Overall**			**1.86**	**(1.39- 2.49)**	**100.0%**
Heterogeneity: $I^2 = 52.1\%$, $\tau^2 = 0.1$, $p = 0.022$					
Test for subgroup differences: $\chi^2_1 = 7.55$, df = 1 ($p = 0.006$)					

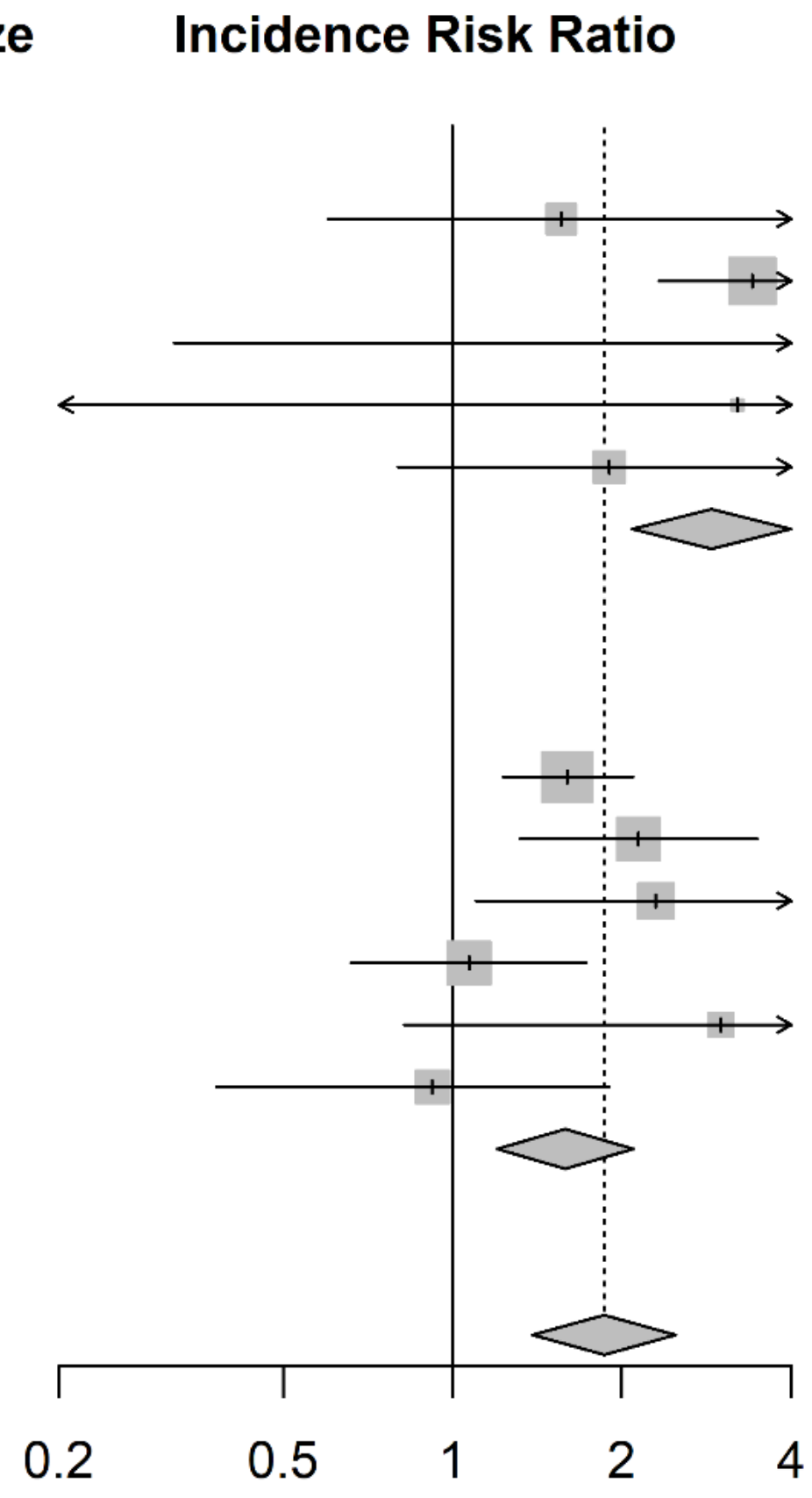

Subgroup analysis of recent exposure to sex work and HCV acquisition risk among male and female PWID, by median proportion female

Study	Country	Sample size	IRR	95% CI	Weight
**0.3+**					
Bach (2016)	Canada	440	3.41	(2.33- 5.00)	7.9%
Boyd, Prins, Jongen and Van Santen (Unpublished)	Netherlands	178	1.69	(0.88- 3.25)	5.2%
Dietze, Sacks-Davis and Kathnayake (Unpublished)	Australia	134	5.22	(0.32-84.04)	0.5%
Hagan (2004)	USA	426	2.90	(1.90- 4.60)	7.2%
Hahn (2002)	USA	195	2.30	(1.10- 4.70)	4.7%
Havens and Young (Unpublished)	USA	277	0.75	(0.28- 2.05)	3.1%
Maher (Unpublished)	Australia	5,650	1.36	(1.05- 1.77)	9.1%
Morris and Nguyen (Unpublished)	USA	1,227	1.07	(0.66- 1.73)	6.8%
Spittal (2012)	Canada	148	1.71	(1.16- 2.53)	7.8%
Strathdee, Abramovitz and Artamonova (Unpublished)	Mexico	150	0.92	(0.38- 1.90)	4.2%
**Overall**			**1.72**	**(1.25- 2.36)**	**56.5%**
Heterogeneity: $I^2 = 70.7\%$, $\tau^2 = 0.2$, $p < 0.001$					
**<0.3**					
Antuori (2021)	Spain	215	1.56	(0.60- 4.03)	3.3%
Blouin and Hankins (Unpublished)	Canada	926	1.60	(1.23- 2.09)	9.1%
Bruneau and Zang (Unpublished)	Canada	449	2.14	(1.32- 3.48)	6.8%
Dumchev (Unpublished)	Ukraine	1,237	0.31	(0.10- 0.96)	2.6%
Mitchell and Clarke (Unpublished)	UK	5,600	1.07	(0.62- 1.85)	6.2%
Moles (2020)	Vietnam	92	0.83	(0.22- 3.18)	2.0%
Platt and Hope (Unpublished)	UK	692	3.00	(0.82-10.99)	2.1%
Roy (2007)	Canada	359	2.31	(1.47- 3.63)	7.1%
Sypsa and Roussos (a) (Unpublished)	Greece	1,630	3.21	(0.19-55.22)	0.5%
Vallejo (2015)	Spain	57	1.90	(0.80- 4.60)	3.7%
**Overall**			**1.57**	**(1.17- 2.11)**	**43.5%**
Heterogeneity: $I^2 = 44.6\%$, $\tau^2 = < 0.1$, $p = 0.062$					
**Overall**			**1.65**	**(1.34- 2.04)**	**100.0%**
Heterogeneity: $I^2 = 59.8\%$, $\tau^2 = 0.1$, $p < 0.001$					
Test for subgroup differences: $\chi^2_1 = 0.17$, df = 1 ($p = 0.678$)					

Incidence Risk Ratio

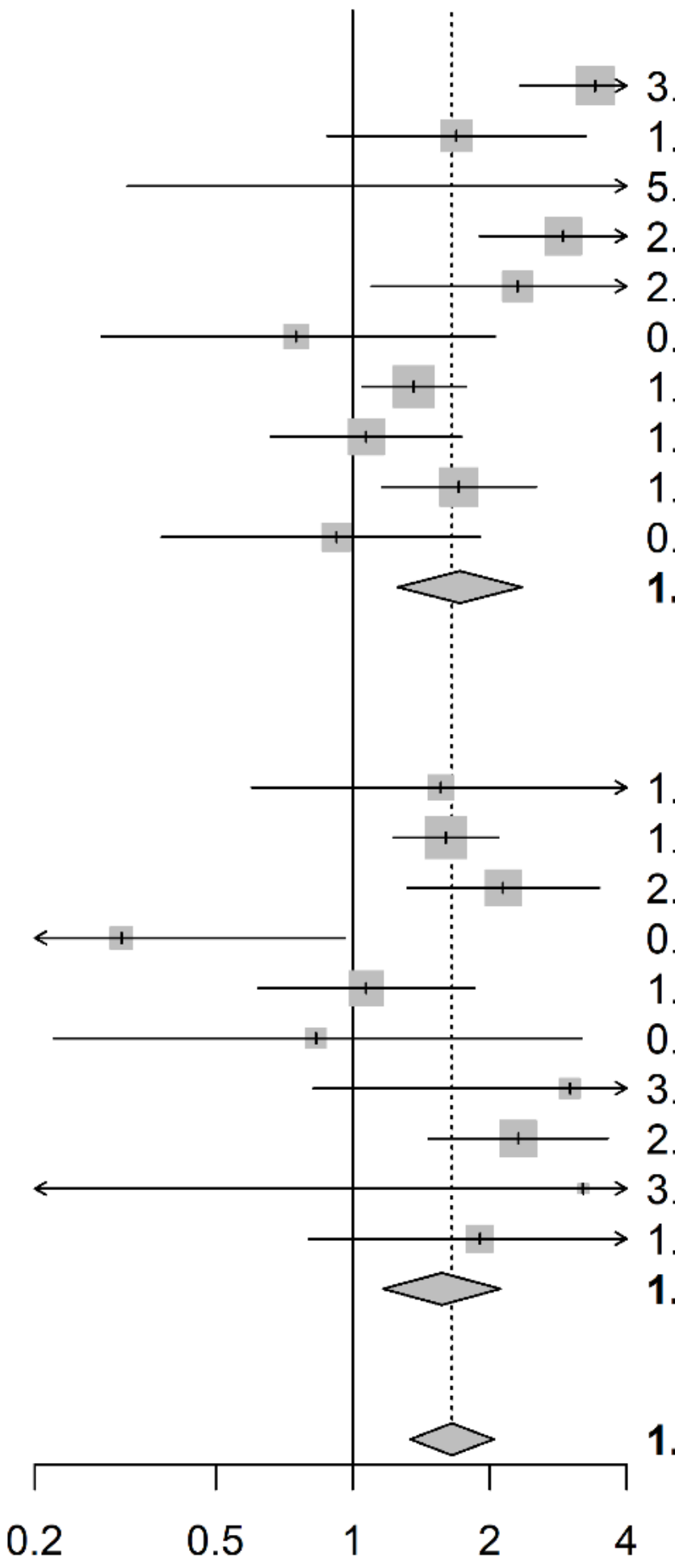

Appendix 12. Subgroup analyses of recent exposure to male-with-male sex and HIV or HCV acquisition risk among male PWID

Subgroup analysis of recent exposure to male-with-male sex and HIV acquisition risk among male PWID, by incidence estimation method

Study	Country	Sample size	IRR	95% CI	Weight
**Direct**					
Blouin and Hankins (Unpublished)	Canada	1,633	2.34	(1.26- 4.36)	13.9%
Bruneau and Zang (Unpublished)	Canada	2,213	1.96	(1.30- 2.95)	16.4%
DeBeck (b) (Unpublished)	Canada	1,216	4.89	(1.67-14.32)	9.0%
Kral (2001)	USA	806	6.90	(3.52-13.49)	13.3%
Martin (2014)	Thailand	1,913	0.75	(0.05-12.20)	2.3%
Mirzazadeh (2018)	USA	369	4.00	(0.65-18.89)	5.1%
Solomon and Mehta (Unpublished)	India	9,170	0.91	(0.37- 2.27)	10.6%
Strathdee (2001)	USA	NR	4.04	(1.50-10.87)	9.8%
Strathdee, Abramovitz and Artamonova (Unpublished)	Mexico	126	3.00	(0.15-57.92)	2.0%
Suntharasamai (2009)	Thailand	NR	0.81	(0.11- 5.87)	3.9%
Sypsa (2017)	Greece	NR	1.85	(0.25-13.50)	3.9%
Sypsa and Roussos (b) (Unpublished)	Greece	952	3.33	(0.45-24.75)	3.9%
**Overall**			**2.62**	**(1.73- 3.98)**	**93.9%**
Heterogeneity: $I^2 = 44.2\%$, $\tau^2 = 0.2$, $p = 0.049$					
**Indirect**					
Lucas (2015)	India	NR	0.47	(0.11- 2.07)	6.1%
**Overall**			**2.35**	**(1.51- 3.66)**	**100.0%**
Heterogeneity: $I^2 = 51.3\%$, $\tau^2 = 0.3$, $p = 0.017$					
Test for subgroup differences: $\chi^2_1 = 4.88$, df = 1 ($p = 0.027$)					

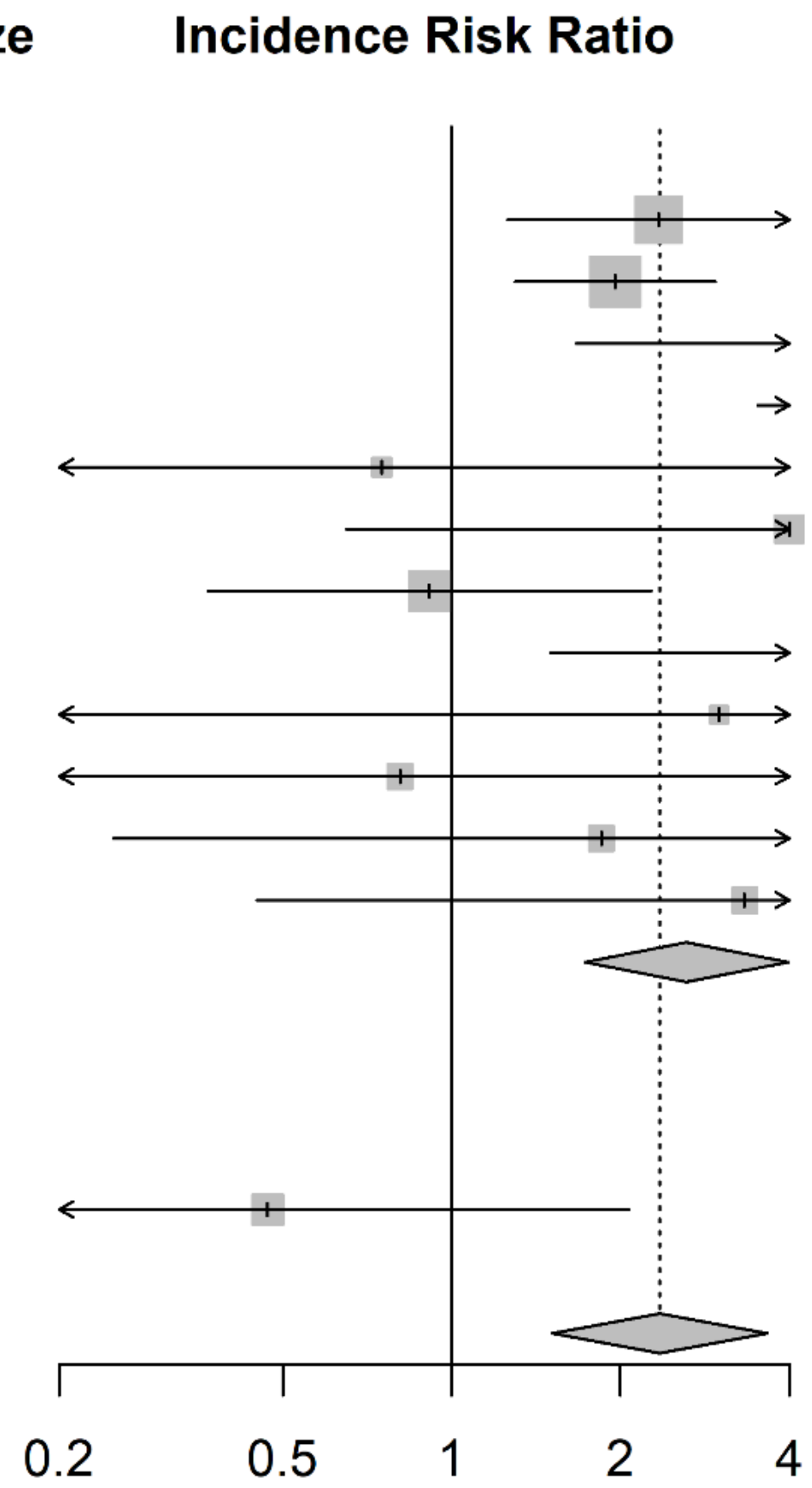

Subgroup analysis of recent exposure to male-with-male sex and HIV acquisition risk among male PWID, by median age of participants

Study	Country	Sample size	IRR	95% CI	Weight
**<35**					
DeBeck (b) (Unpublished)	Canada	1,216	4.89	(1.67-14.32)	9.5%
Lucas (2015)	India	NR	0.47	(0.11- 2.07)	6.6%
Martin (2014)	Thailand	1,913	0.75	(0.05-12.20)	2.5%
Solomon and Mehta (Unpublished)	India	9,170	0.91	(0.37- 2.27)	11.1%
Suntharasamai (2009)	Thailand	NR	0.81	(0.11- 5.87)	4.3%
**Overall**			**1.20**	**(0.46- 3.10)**	**34.0%**
Heterogeneity: I^2 = 53.7%, τ^2 = 0.6, p = 0.071					
**35+**					
Blouin and Hankins (Unpublished)	Canada	1,633	2.34	(1.26- 4.36)	14.4%
Bruneau and Zang (Unpublished)	Canada	2,213	1.96	(1.30- 2.95)	16.9%
Kral (2001)	USA	806	6.90	(3.52-13.49)	13.8%
Strathdee (2001)	USA	NR	4.04	(1.50-10.87)	10.3%
Strathdee, Abramovitz and Artamonova (Unpublished)	Mexico	126	3.00	(0.15-57.92)	2.2%
Sypsa (2017)	Greece	NR	1.85	(0.25-13.50)	4.2%
Sypsa and Roussos (b) (Unpublished)	Greece	952	3.33	(0.45-24.75)	4.2%
**Overall**			**3.06**	**(1.91- 4.88)**	**66.0%**
Heterogeneity: I^2 = 44.7%, τ^2 = 0.1, p = 0.093					
**Overall**			**2.28**	**(1.43- 3.62)**	**100.0%**
Heterogeneity: I^2 = 54.7%, τ^2 = 0.3, p = 0.012					
Test for subgroup differences: χ^2_1 = 3.00, df = 1 (p = 0.083)					

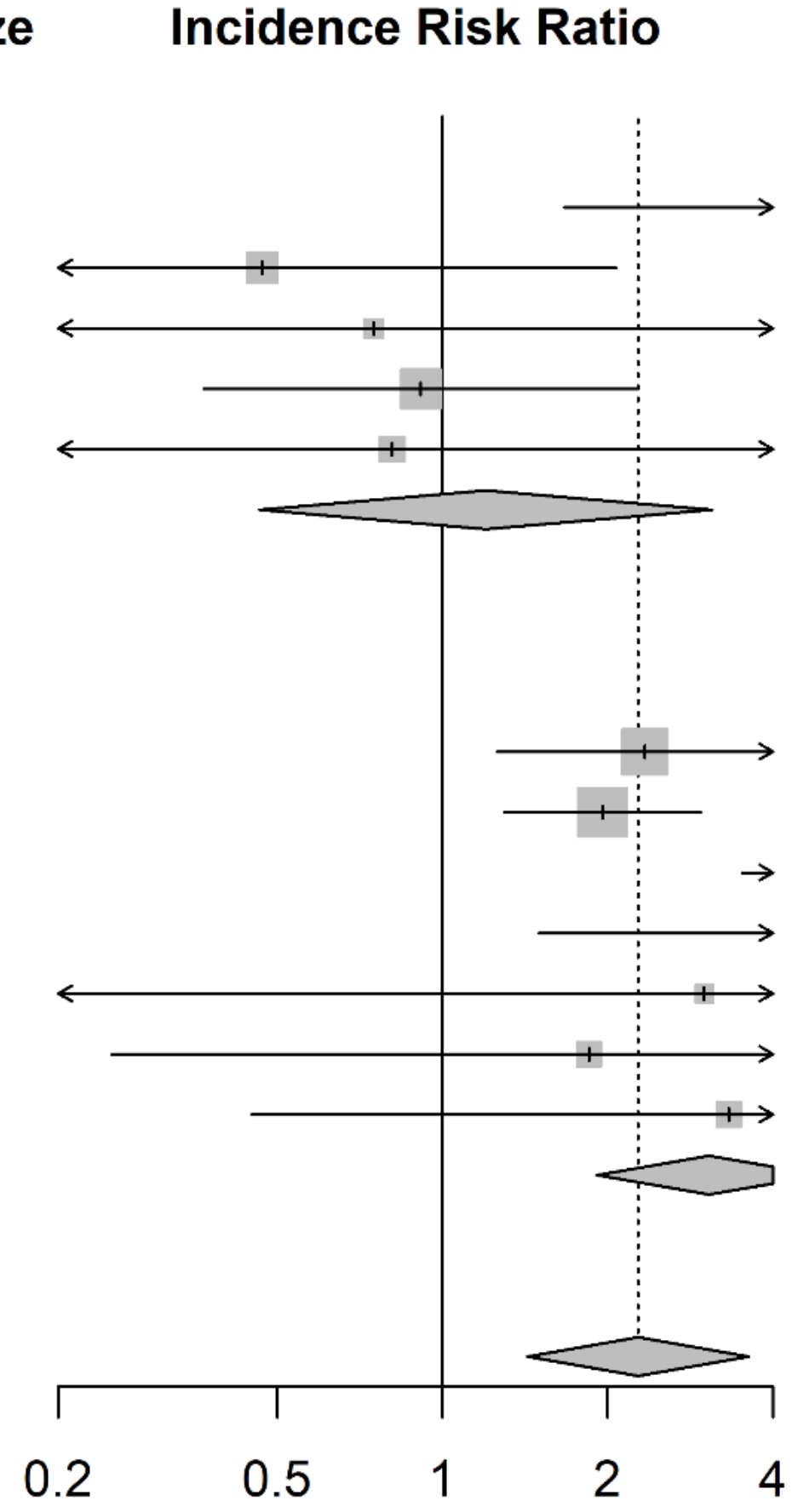

Subgroup analysis of recent exposure to male-with-male sex and HIV acquisition risk among male PWID, by median proportion recently incarcerated

Study	Country	Sample size	IRR	95% CI	Weight
**0.1+**					
Blouin and Hankins (Unpublished)	Canada	1,633	2.34	(1.26- 4.36)	28.9%
DeBeck (b) (Unpublished)	Canada	1,216	4.89	(1.67-14.32)	18.8%
Lucas (2015)	India	NR	0.47	(0.11- 2.07)	13.0%
Martin (2014)	Thailand	1,913	0.75	(0.05-12.20)	4.8%
**Overall**			**1.83**	**(0.72- 4.69)**	**65.5%**
Heterogeneity: I^2 = 57.1%, τ^2 = 0.5, p = 0.072					
**<0.1**					
Solomon and Mehta (Unpublished)	India	9,170	0.91	(0.37- 2.27)	22.1%
Strathdee, Abramovitz and Artamonova (Unpublished)	Mexico	126	3.00	(0.15-57.92)	4.2%
Sypsa and Roussos (b) (Unpublished)	Greece	952	3.33	(0.45-24.75)	8.2%
**Overall**			**1.22**	**(0.55- 2.71)**	**34.5%**
Heterogeneity: I^2 = 0%, τ^2 = 0, p = 0.426					
**Overall**			**1.75**	**(0.92- 3.33)**	**100.0%**
Heterogeneity: I^2 = 41.2%, τ^2 = 0.3, p = 0.116					
Test for subgroup differences: χ^2_1 = 0.42, df = 1 (p = 0.517)					

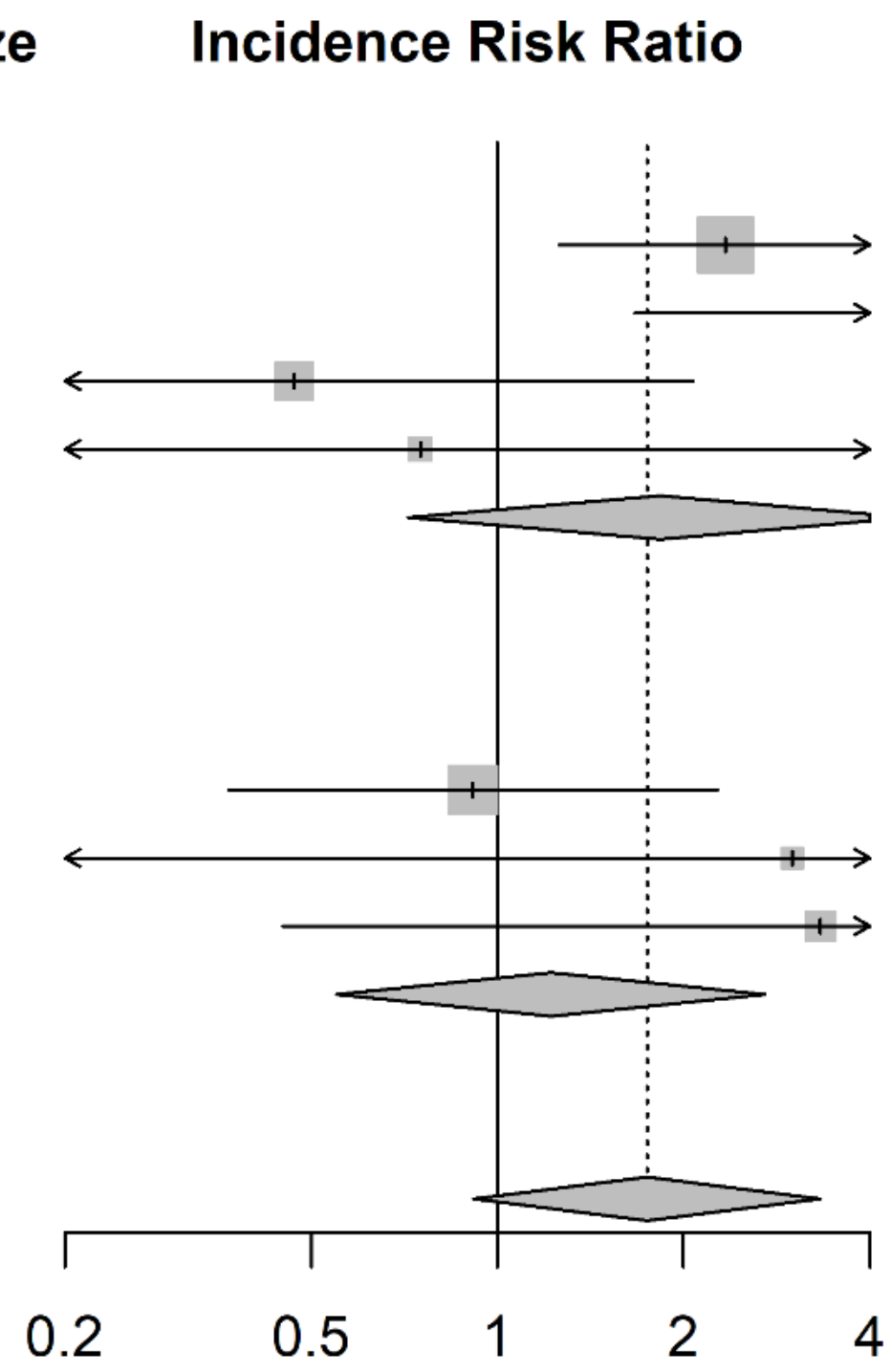

Subgroup analysis of recent exposure to male-with-male sex and HIV acquisition risk among male PWID, by median proportion recent or current OAT receipt

Study	Country	Sample size	IRR	95% CI	Weight
**0.2+**					
DeBeck (b) (Unpublished)	Canada	1,216	4.89	(1.67-14.32)	10.8%
Kral (2001)	USA	806	6.90	(3.52-13.49)	15.5%
Mirzazadeh (2018)	USA	369	4.00	(0.65-18.89)	6.3%
Solomon and Mehta (Unpublished)	India	9,170	0.91	(0.37- 2.27)	12.6%
Sypsa (2017)	Greece	NR	1.85	(0.25-13.50)	4.9%
**Overall**			**3.09**	**(1.24- 7.69)**	**50.1%**
Heterogeneity: I^2 = 69.5%, τ^2 = 0.7, p = 0.011					
**<0.2**					
Blouin and Hankins (Unpublished)	Canada	1,633	2.34	(1.26- 4.36)	16.1%
Bruneau and Zang (Unpublished)	Canada	2,213	1.96	(1.30- 2.95)	18.7%
Lucas (2015)	India	NR	0.47	(0.11- 2.07)	7.6%
Strathdee, Abramovitz and Artamonova (Unpublished)	Mexico	126	3.00	(0.15-57.92)	2.5%
Sypsa and Roussos (b) (Unpublished)	Greece	952	3.33	(0.45-24.75)	4.9%
**Overall**			**1.94**	**(1.34- 2.80)**	**49.9%**
Heterogeneity: I^2 = 6.9%, τ^2 = < 0.1, p = 0.367					
**Overall**			**2.39**	**(1.44- 3.95)**	**100.0%**
Heterogeneity: I^2 = 58.6%, τ^2 = 0.3, p = 0.010					
Test for subgroup differences: χ^2_1 = 0.85, df = 1 (p = 0.356)					

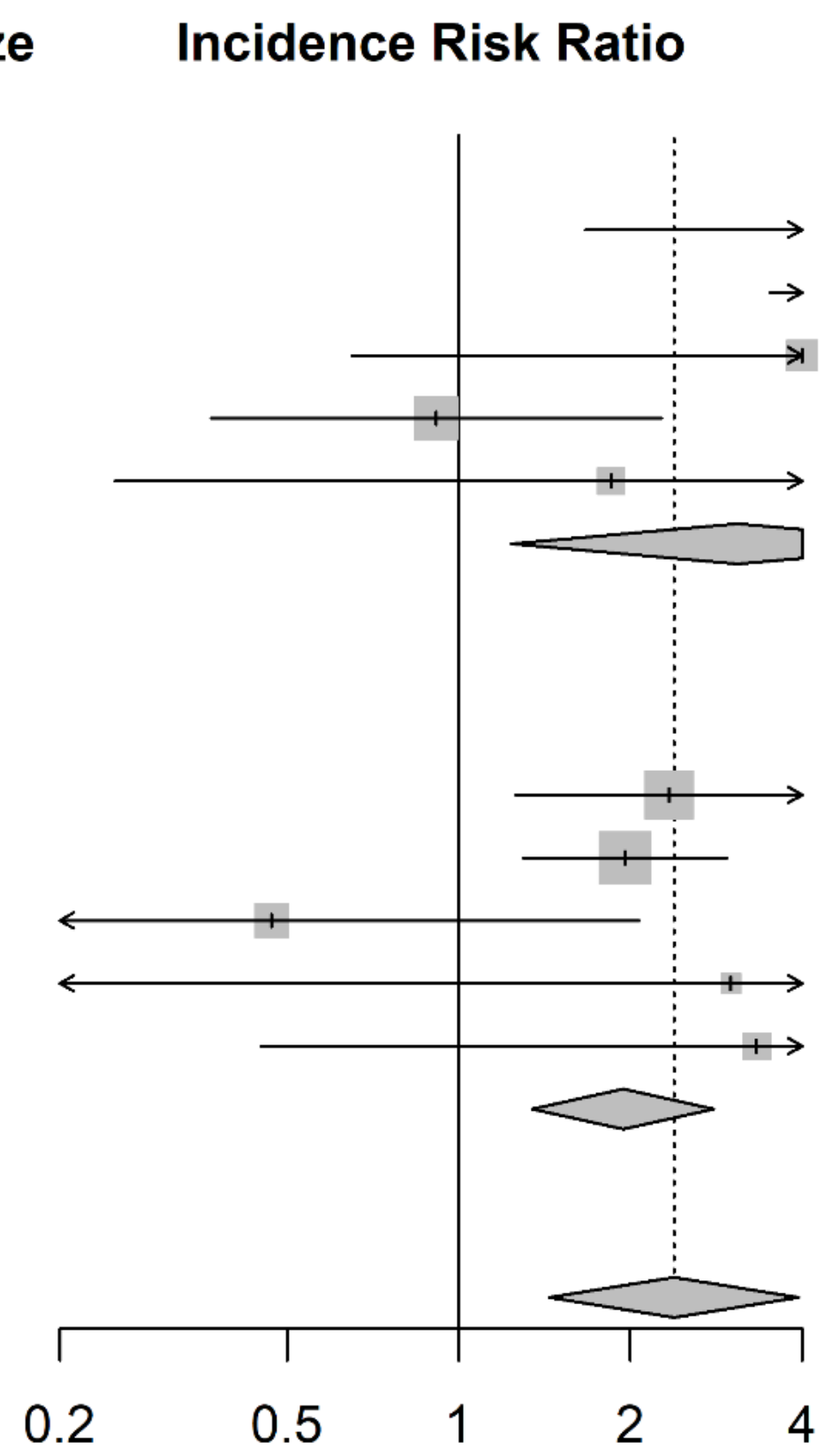

Subgroup analysis of recent exposure to male-with-male sex and HIV acquisition risk among male PWID, by median proportion recently homeless

Study	Country	Sample size	IRR	95% CI	Weight
**<0.4**					
Kral (2001)	USA	806	6.90	(3.52-13.49)	17.3%
Solomon and Mehta (Unpublished)	India	9,170	0.91	(0.37- 2.27)	13.4%
Sypsa (2017)	Greece	NR	1.85	(0.25-13.50)	4.7%
Sypsa and Roussos (b) (Unpublished)	Greece	952	3.33	(0.45-24.75)	4.7%
**Overall**			**2.56**	**(0.74- 8.82)**	**40.1%**
Heterogeneity: $I^2 = 76.2\%$, $\tau^2 = 1.1$, $p = 0.006$					
**0.4+**					
Blouin and Hankins (Unpublished)	Canada	1,633	2.34	(1.26- 4.36)	18.2%
Bruneau and Zang (Unpublished)	Canada	2,213	1.96	(1.30- 2.95)	22.0%
DeBeck (b) (Unpublished)	Canada	1,216	4.89	(1.67-14.32)	11.3%
Mirzazadeh (2018)	USA	369	4.00	(0.65-18.89)	6.1%
Strathdee, Abramovitz and Artamonova (Unpublished)	Mexico	126	3.00	(0.15-57.92)	2.3%
**Overall**			**2.29**	**(1.67- 3.15)**	**59.9%**
Heterogeneity: $I^2 = 0\%$, $\tau^2 = 0$, $p = 0.570$					
**Overall**			**2.71**	**(1.68- 4.37)**	**100.0%**
Heterogeneity: $I^2 = 52.5\%$, $\tau^2 = 0.2$, $p = 0.032$					
Test for subgroup differences: $\chi^2_1 = 0.03$, df = 1 ($p = 0.865$)					

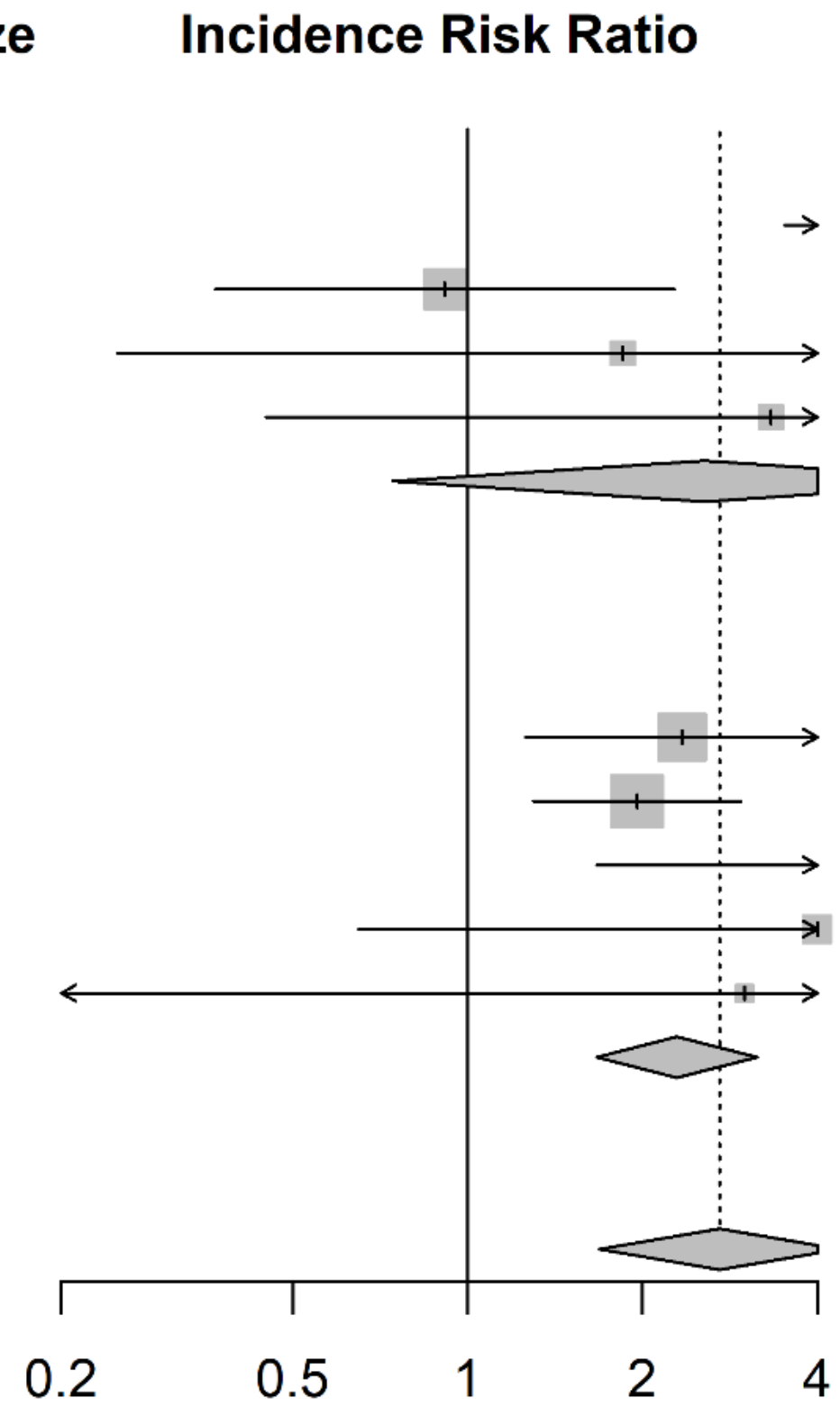

Subgroup analysis of recent exposure to male-with-male sex and HCV acquisition risk among male PWID, by incidence estimation method

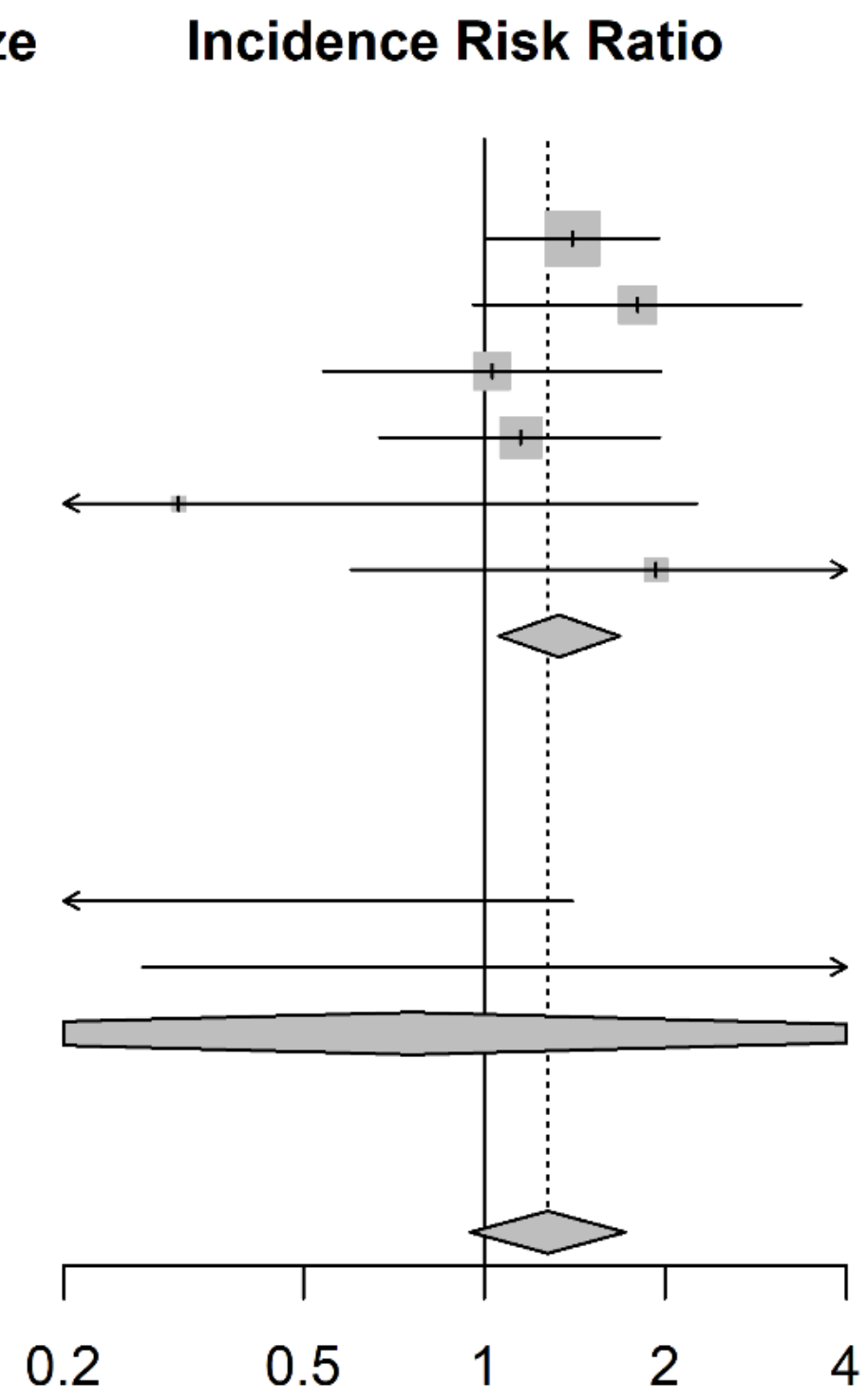

Study	Country	Sample size	IRR	95% CI	Weight
**Direct**					
Blouin and Hankins (Unpublished)	Canada	689	1.40	(1.01- 1.95)	35.5%
Bruneau and Zang (Unpublished)	Canada	361	1.80	(0.96- 3.36)	16.6%
DeBeck (b) (Unpublished)	Canada	521	1.03	(0.54- 1.97)	15.8%
Roy (2007)	Canada	NR	1.15	(0.67- 1.96)	20.6%
Strathdee, Abramovitz and Artamonova (Unpublished)	Mexico	103	0.31	(0.04- 2.26)	2.1%
Todd (Unpublished)	Afghanistan	479	1.93	(0.60- 6.16)	5.9%
**Overall**			**1.33**	**(1.06- 1.68)**	**96.5%**
Heterogeneity: $I^2 = 0\%$, $\tau^2 = 0$, $p = 0.512$					
**Indirect**					
Mitchell and Clarke (Unpublished)	UK	4,631	0.19	(0.03- 1.40)	2.3%
Platt and Hope (Unpublished)	UK	731	4.20	(0.27-64.90)	1.2%
**Overall**			**0.76**	**(0.04-15.55)**	**3.5%**
Heterogeneity: $I^2 = 69.6\%$, $\tau^2 = 3.3$, $p = 0.070$					
**Overall**			**1.28**	**(0.95- 1.72)**	**100.0%**
Heterogeneity: $I^2 = 21.0\%$, $\tau^2 < 0.1$, $p = 0.263$					
Test for subgroup differences: $\chi^2_1 = 0.13$, df = 1 ($p = 0.716$)					

Subgroup analysis of recent exposure to male-with-male sex and HCV acquisition risk among male PWID, by median age of participants

Study	Country	Sample size	IRR	95% CI	Weight
**<32.5**					
DeBeck (b) (Unpublished)	Canada	521	1.03	(0.54- 1.97)	15.8%
Platt and Hope (Unpublished)	UK	731	4.20	(0.27-64.90)	1.2%
Roy (2007)	Canada	NR	1.15	(0.67- 1.96)	20.6%
Todd (Unpublished)	Afghanistan	479	1.93	(0.60- 6.16)	5.9%
**Overall**			**1.20**	**(0.82- 1.77)**	**43.5%**
Heterogeneity: $I^2 = 0\%$, $\tau^2 = 0$, $p = 0.641$					
**32.5+**					
Blouin and Hankins (Unpublished)	Canada	689	1.40	(1.01- 1.95)	35.5%
Bruneau and Zang (Unpublished)	Canada	361	1.80	(0.96- 3.36)	16.6%
Mitchell and Clarke (Unpublished)	UK	4,631	0.19	(0.03- 1.40)	2.3%
Strathdee, Abramovitz and Artamonova (Unpublished)	Mexico	103	0.31	(0.04- 2.26)	2.1%
**Overall**			**1.11**	**(0.58- 2.13)**	**56.5%**
Heterogeneity: $I^2 = 56.5\%$, $\tau^2 = 0.2$, $p = 0.075$					
**Overall**			**1.28**	**(0.95- 1.72)**	**100.0%**
Heterogeneity: $I^2 = 21.0\%$, $\tau^2 < 0.1$, $p = 0.263$					
Test for subgroup differences: $\chi^2_1 = 0.04$, df = 1 ($p = 0.841$)					

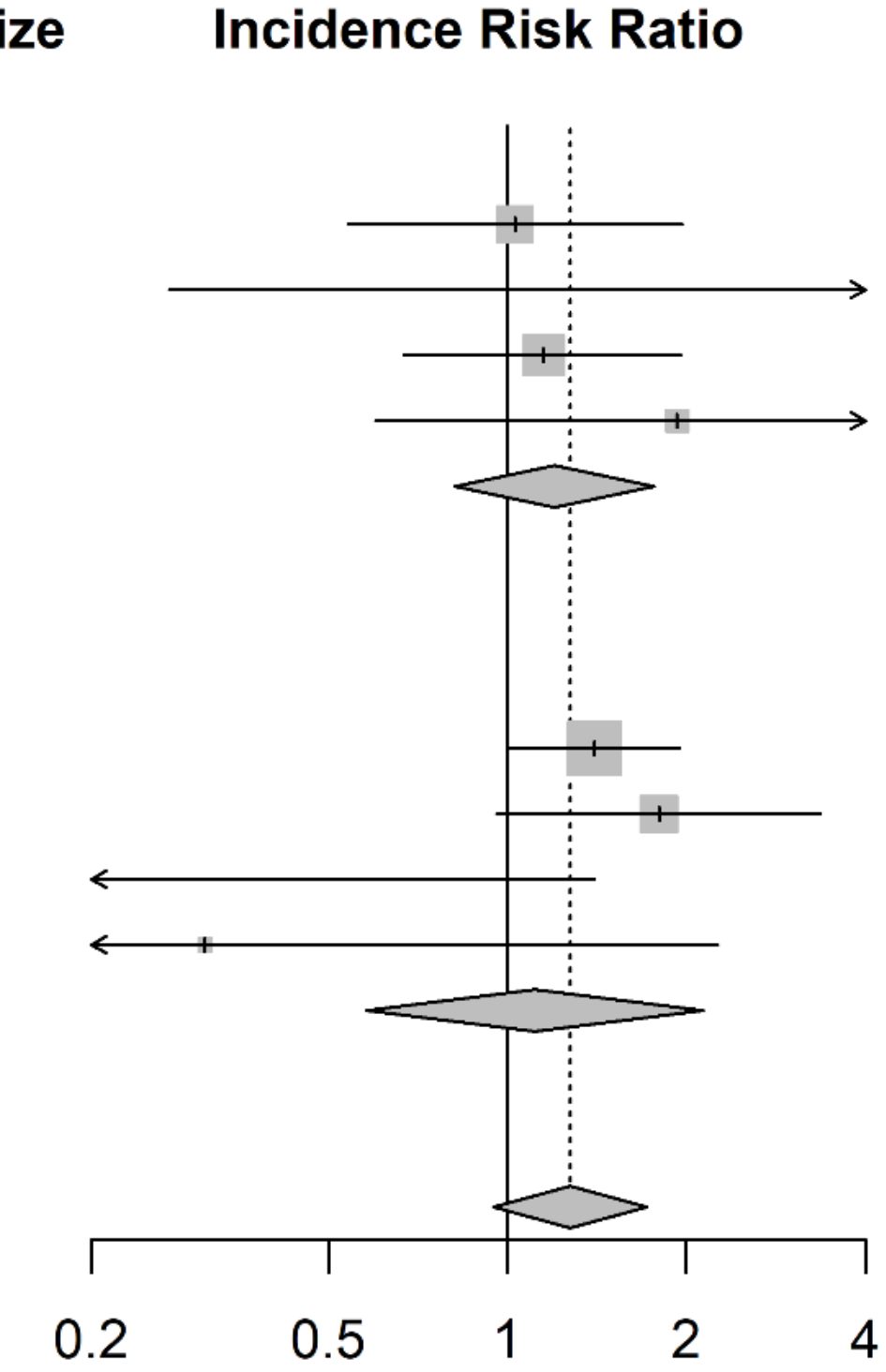

Subgroup analysis of recent exposure to male-with-male sex and HCV acquisition risk among male PWID, by median proportion recently incarcerated

Study	Country	Sample size	IRR	95% CI	Weight
**<0.1**					
Blouin and Hankins (Unpublished)	Canada	689	1.40	(1.01- 1.95)	60.4%
Strathdee, Abramovitz and Artamonova (Unpublished)	Mexico	103	0.31	(0.04- 2.26)	1.6%
Todd (Unpublished)	Afghanistan	479	1.93	(0.60- 6.16)	4.8%
**Overall**			**1.35**	**(0.80- 2.26)**	**66.9%**
Heterogeneity: I^2 = 17.7%, τ^2 = < 0.1, p = 0.297					
**0.1+**					
Bruneau and Zang (Unpublished)	Canada	361	1.80	(0.96- 3.36)	16.7%
DeBeck (b) (Unpublished)	Canada	521	1.03	(0.54- 1.97)	15.6%
Platt and Hope (Unpublished)	UK	731	4.20	(0.27-64.90)	0.9%
**Overall**			**1.42**	**(0.89- 2.25)**	**33.1%**
Heterogeneity: I^2 = 4.6%, τ^2 = < 0.1, p = 0.350					
**Overall**			**1.39**	**(1.08- 1.80)**	**100.0%**
Heterogeneity: I^2 = 0.0%, τ^2 = 0, p = 0.475					
Test for subgroup differences: χ^2_1 = 0.02, df = 1 (p = 0.883)					

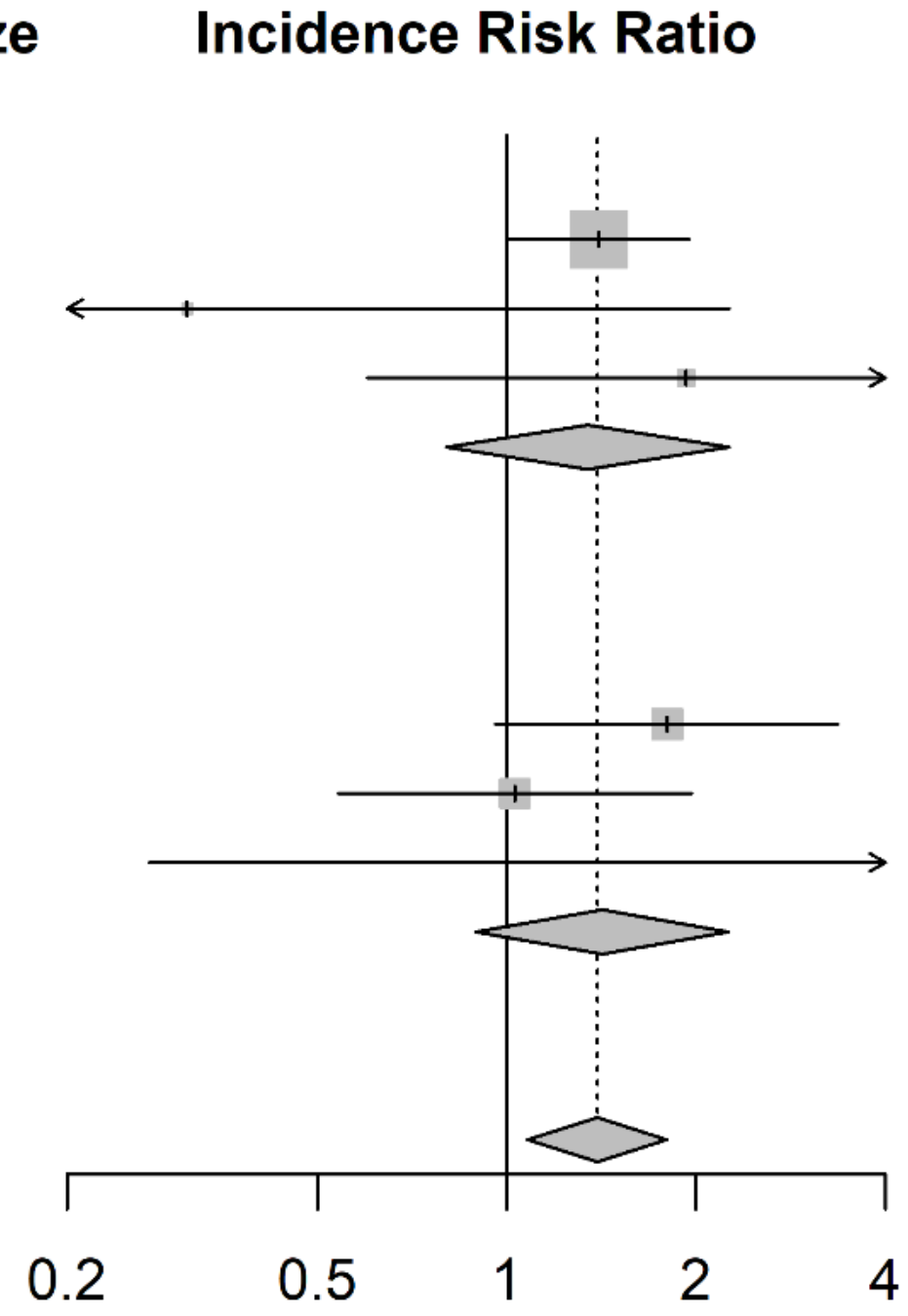

Subgroup analysis of recent exposure to male-with-male sex and HCV acquisition risk among male PWID, by median proportion recent or current OAT receipt

Study	Country	Sample size	Incidence Risk Ratio	IRR	95% CI	Weight
**<0.2**						
Blouin and Hankins (Unpublished)	Canada	689		1.40	(1.01- 1.95)	59.5%
Strathdee, Abramovitz and Artamonova (Unpublished)	Mexico	103		0.31	(0.04- 2.26)	2.0%
**Overall**				**0.93**	**(0.25- 3.46)**	**61.5%**
Heterogeneity: I^2 = 52.2%, τ^2 = 0.6, p = 0.148						
**0.2+**						
Bruneau and Zang (Unpublished)	Canada	361		1.80	(0.96- 3.36)	19.3%
DeBeck (b) (Unpublished)	Canada	521		1.03	(0.54- 1.97)	18.1%
Platt and Hope (Unpublished)	UK	731		4.20	(0.27-64.90)	1.1%
**Overall**				**1.42**	**(0.89- 2.25)**	**38.5%**
Heterogeneity: I^2 = 4.6%, τ^2 = < 0.1, p = 0.350						
**Overall**				**1.37**	**(1.03- 1.81)**	**100.0%**
Heterogeneity: I^2 = 5.2%, τ^2 < 0.1, p = 0.377						
Test for subgroup differences: χ^2_1 = 0.35, df = 1 (p = 0.551)						

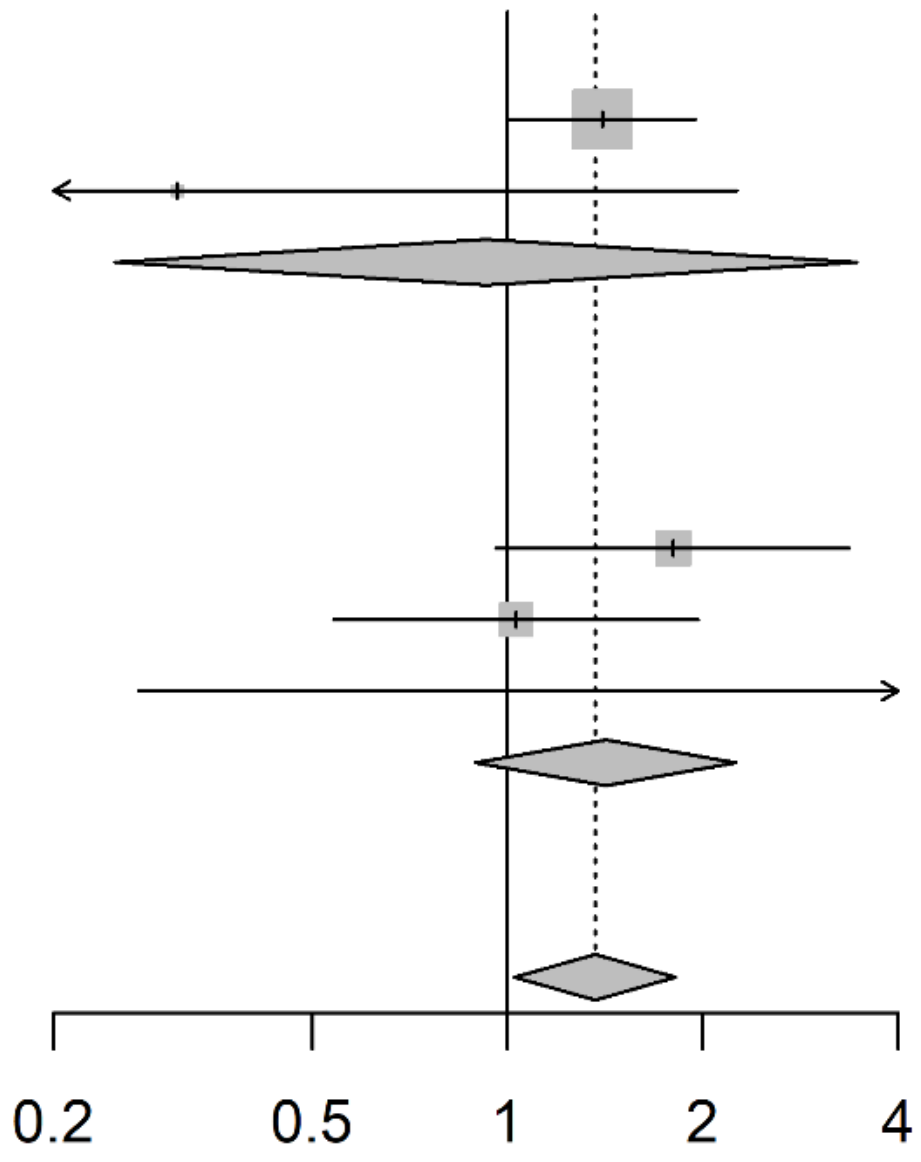

Subgroup analysis of recent exposure to male-with-male sex and HCV acquisition risk among male PWID, by median proportion recently homeless

Study	Country	Sample size	IRR	95% CI	Weight
**<0.5**					
Blouin and Hankins (Unpublished)	Canada	689	1.40	(1.01- 1.95)	60.4%
Bruneau and Zang (Unpublished)	Canada	361	1.80	(0.96- 3.36)	16.7%
Todd (Unpublished)	Afghanistan	479	1.93	(0.60- 6.16)	4.8%
**Overall**			**1.50**	**(1.13- 1.99)**	**81.9%**
Heterogeneity: I^2 = 0%, τ^2 = 0, p = 0.714					
**0.5+**					
DeBeck (b) (Unpublished)	Canada	521	1.03	(0.54- 1.97)	15.6%
Platt and Hope (Unpublished)	UK	731	4.20	(0.27-64.90)	0.9%
Strathdee, Abramovitz and Artamonova (Unpublished)	Mexico	103	0.31	(0.04- 2.26)	1.6%
**Overall**			**0.97**	**(0.42- 2.25)**	**18.1%**
Heterogeneity: I^2 = 15.1%, τ^2 = 0.1, p = 0.308					
**Overall**			**1.39**	**(1.08- 1.80)**	**100.0%**
Heterogeneity: I^2 = 0.0%, τ^2 = 0, p = 0.475					
Test for subgroup differences: χ^2_1 = 0.93, df = 1 (p = 0.334)					

Incidence Risk Ratio

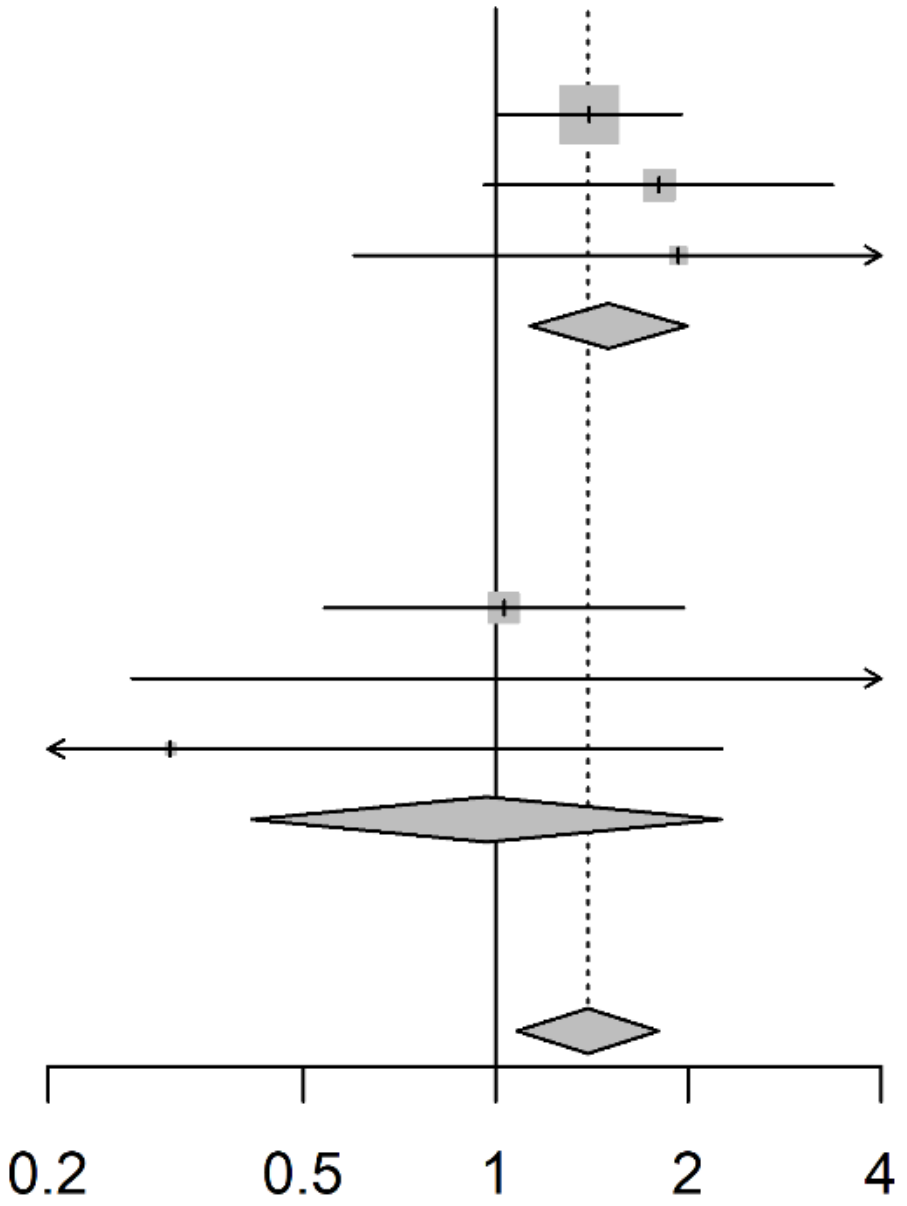

Appendix 13. Sensitivity analyses of association between male-with-male sex and risk of HCV acquisition, excluding studies where less than 100% of sample are male PWID

Meta-analysis of studies reporting the crude pooled association between recent exposure to male-with-male sex and HCV acquisition risk among male PWID, excluding studies where less than 100% of sample are male PWID

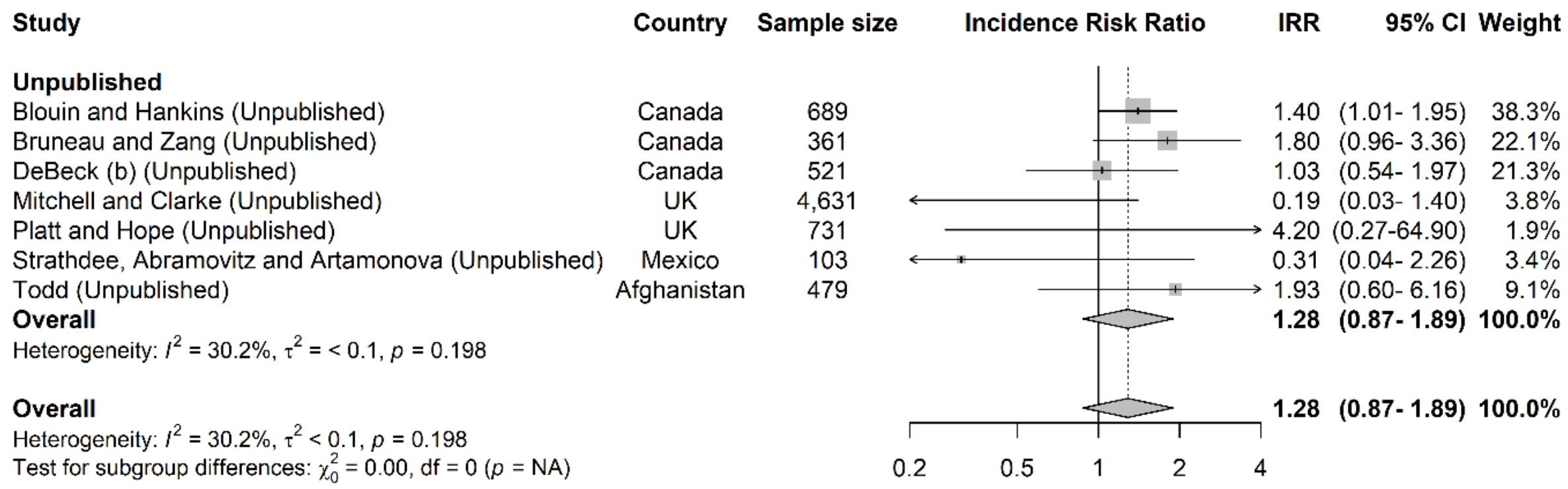

Meta-analysis of studies reporting the crude pooled association between lifetime exposure to male-with-male sex and HCV acquisition risk among male PWID, excluding studies where less than 100% of sample are male PWID

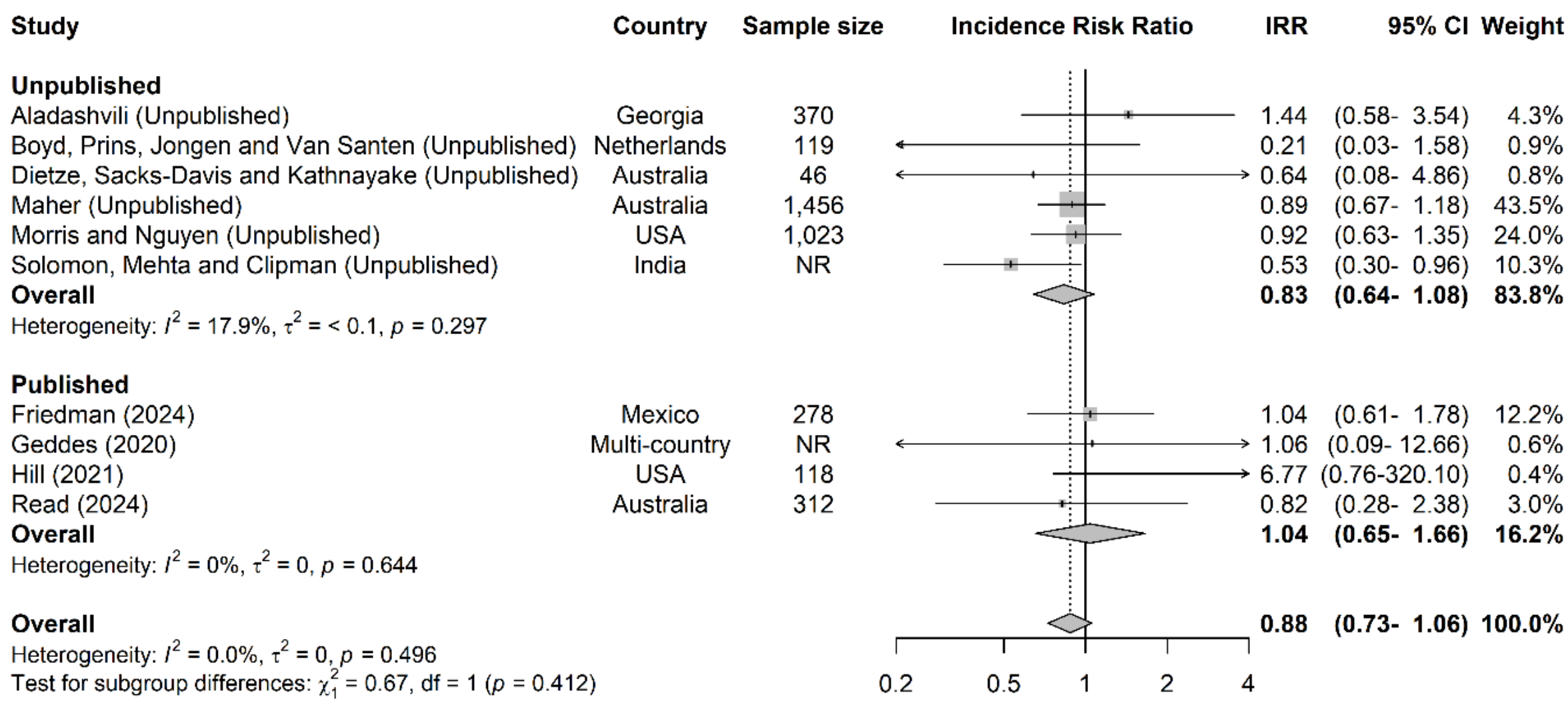

Appendix 14. Publication asymmetry

Funnel plot of studies included in the meta-analysis of crude estimates of recent exposure to sex work or male-with-male sexual exposure and HIV or HCV incidence

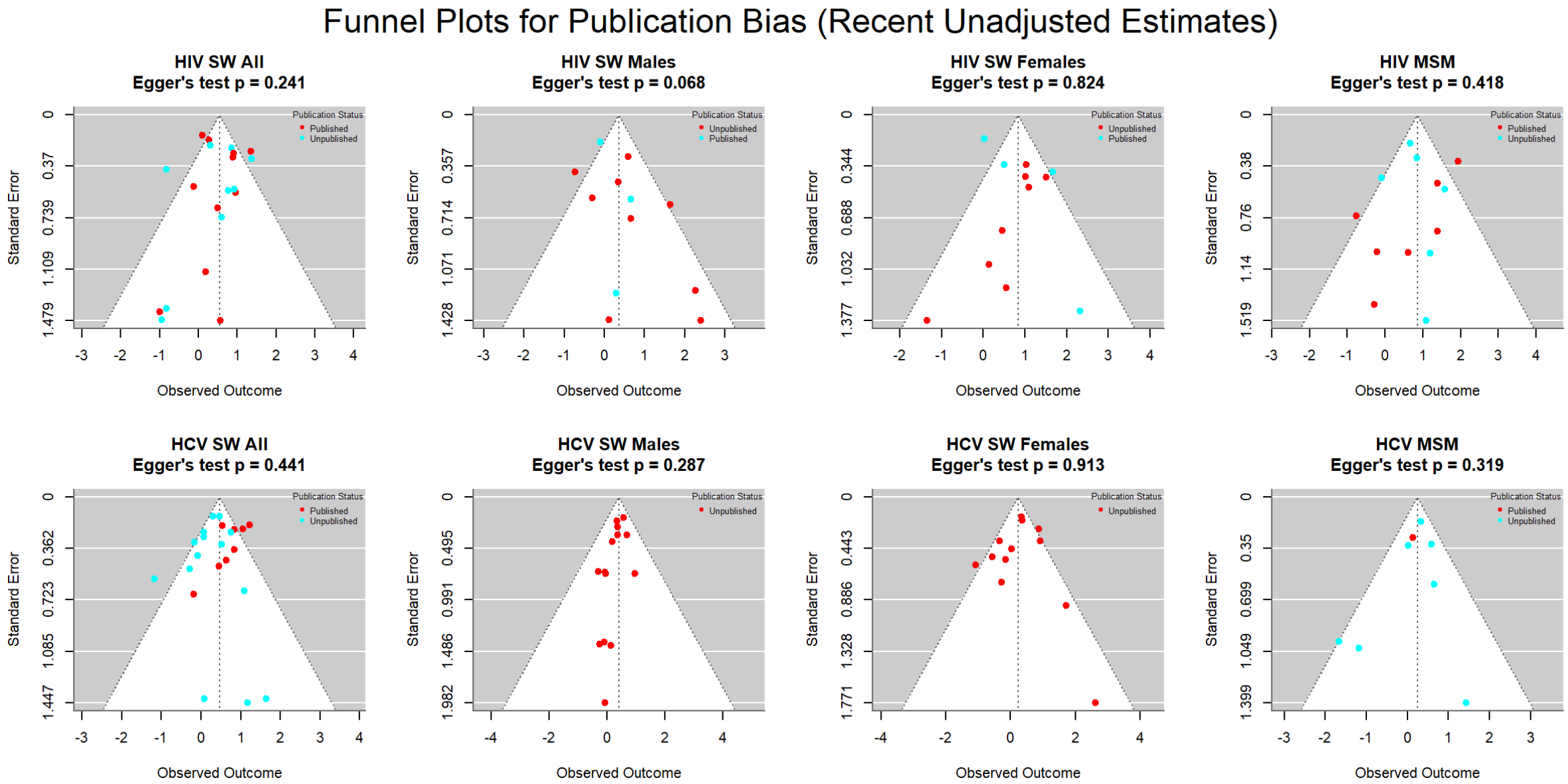

Funnel plot of studies included in the meta-analysis of lifetime exposure to sex work or male-with-male sexual exposure and HIV or HCV incidence

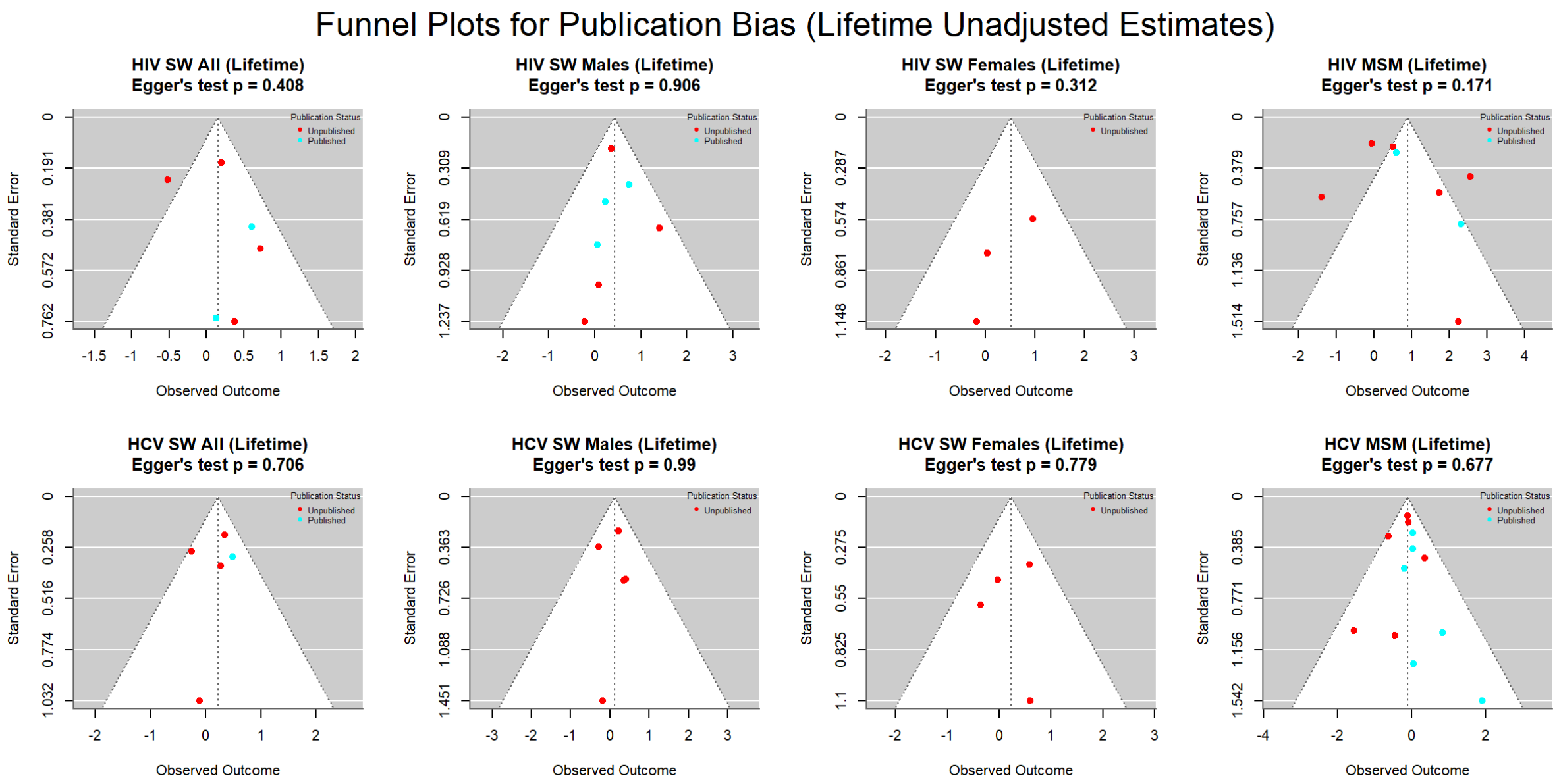

Egger's test stratified by publication status

Exposure	Sex/gender	Outcome	Publication status	P-value
Sex work	Males and females	HIV	Published	0.570
Sex work	Males and females	HIV	Unpublished	0.281
Sex work	Males	HIV	Published	0.331
Sex work	Males	HIV	Unpublished	0.128
Sex work	Females	HIV	Published	0.089
Sex work	Females	HIV	Unpublished	0.057
Male-with-male sex	Males	HIV	Published	0.011
Male-with-male sex	Males	HIV	Unpublished	0.577
Sex work	Males and females	HCV	Published	0.091
Sex work	Males and females	HCV	Unpublished	0.792
Sex work	Males	HCV	Published	-
Sex work	Males	HCV	Unpublished	0.287
Sex work	Females	HCV	Published	-
Sex work	Females	HCV	Unpublished	0.913
Male-with-male sex	Males	HCV	Published	-
Male-with-male sex	Males	HCV	Unpublished	0.316

Appendix 15. Study quality of published studies for the effect of sex work or male-with-male sexual exposure on HIV and HCV acquisition risk

Lead author	Representativeness of the cohort	Selection of the non-exposed cohort	Ascertainment of exposure	Demonstration that outcome of interest was not present at start of study	Comparability of cohorts on the basis of the design or analysis controlled for confounders	Assessment of incident infection	Was follow-up testing schedule adequate?	Adequacy of follow-up of cohorts	Total score (max: 8)
Antuori	1	1	1	1	0	1	1	1	7
Bach	1	1	1	1	0	1	1	1	7
Blouin	1	1	1	1	1	1	1	0	7
Booth	1	1	1	1	0	1	1	1	7
Friedman	1	1	1	1	0	1	1	1	7
Geddes	1	1	0	1	0	1	1	1	6
Hagan	1	1	1	1	0	1	1	0	6
Hahn	0	1	1	1	1	1	1	0	6
Hill	0	0	0	1	0	1	1	0	3
Jain	1	1	1	1	1	1	1	1	8
Kozlov	1	1	1	1	0	1	1	0	6
Kozlov	1	1	1	1	1	1	1	1	8
Kral	1	1	1	1	1	1	1	0	7
Kulchynska	1	1	1	1	0	1	1	1	7
Lucas	1	1	1	1	0	1	1	1	7
Martin	1	1	1	1	0	1	1	0	6
Martinello	0	0	1	1	0	1	1	1	5
Mehta	0	1	1	1	1	1	1	0	6
Mirzazadeh	0	1	1	1	1	1	1	0	6
Moles	1	1	1	1	0	1	1	0	6
Read	0	1	0	1	0	1	0	0	3
Roy	1	1	1	1	1	1	1	0	7

Roy	1	1	1	1	1	1	1	0	7
Scheim	1	1	1	1	1	1	1	0	7
Skaathun	1	1	1	1	0	1	1	0	6
Spada	1	1	0	1	0	1	1	0	5
Spittal	0	1	1	1	0	1	1	1	6
Strathdee	1	1	1	1	1	1	1	0	7
Suntharasamai	1	1	1	1	0	1	1	1	7
Sypsa	1	1	1	1	0	1	1	0	6
Vallejo	0	1	1	1	0	1	1	0	5
Yin	1	1	1	1	1	1	1	0	7
Zhang	1	1	0	1	0	1	1	1	6

Appendix 16. Study quality of unpublished studies for the effect of sex work or male-with-male sexual exposure on HIV and HCV acquisition risk

Lead author	Representativeness of the cohort	Selection of the non-exposed cohort	Ascertainment of exposure	Demonstration that outcome of interest was not present at start of study	Comparability of cohorts on the basis of the design or analysis controlled for confounders	Assessment of incident infection	Was follow-up testing schedule adequate?	Adequacy of follow-up of cohorts	Total score (max: 8)
Aladashvili	1	1	1	1	1	1	1	0	7
Blouin and Hankins	1	1	1	1	1	1	1	0	7
Boyd, Prins, Jongen and Van Santen	1	1	1	1	1	1	1	0	7
Bruneau and Zang	1	1	1	1	1	1	1	1	8
Debeck	1	1	1	1	1	1	1	0	7
Debeck	1	1	1	1	1	1	1	0	7
Dietze, Sacks-Davis and Kathnayake	1	1	1	1	0	1	1	0	6
Dumchev	1	1	1	1	1	1	1	0	7
Havens and Young	1	1	1	1	1	1	1	1	8
Platt and Hope	1	1	1	1	1	1	1	1	8
Kurth and Walker	1	1	1	1	1	1	1	0	7
Maher	1	1	1	1	1	1	1	1	8
Mitchell and Clarke	1	1	1	1	1	1	1	1	8
Morris and Nguyen	0	1	1	1	1	1	1	0	6
Mravcik	1	1	1	1	1	1	1	0	7
Palmateer	1	1	1	1	0	1	1	1	7
Platt	1	1	1	1	1	1	1	1	8
Sacks-Davis	1	1	1	1	0	1	1	0	6
Sarna and Ray Saraswati	1	1	1	1	1	1	1	0	7

Solomon and Mehta	1	1	1	1	1	1	1	0	7
Solomon, Mehta and Clipman	1	1	1	1	1	1	1	1	8
Strathdee, Abramovitz and Artamonova	1	1	1	1	1	1	1	1	8
Sypsa and Roussos	1	1	1	1	0	1	1	0	6
Sypsa and Roussos	1	1	1	1	1	1	1	0	7
Todd	1	1	1	1	1	1	1	1	8
Trickey and Des Jarlais	1	1	1	1	0	1	1	0	6

References

1. Antuori A, Montoya V, Pineyro D, Sumoy L, Joy J, Krajden M, et al. Characterization of Acute HCV Infection and Transmission Networks in People Who Currently Inject Drugs in Catalonia: Usefulness of Dried Blood Spots. Hepatology. 2021.
2. Bach P, Walton G, Hayashi K, Milloy MJ, Dong H, Kerr T, et al. Benzodiazepine Use and Hepatitis C Seroconversion in a Cohort of Persons Who Inject Drugs. American Journal of Public Health. 2016;106(6):1067-72.
3. Blouin K, Blanchette C, Leclerc P, Morissette C, Vallee M, Diabate S. HIV and HCV seroincidence, associated factors and drug use in people who inject drugs, SurvUDI network, eastern central Canada, 1995-2020. International Journal of Drug Policy. 2024;(no pagination).
4. Booth RE, Davis JM, Dvoryak S, Brewster JT, Lisovska O, Strathdee SA, et al. HIV incidence among people who inject drugs (PWIDs) in Ukraine: results from a clustered randomised trial. Lancet HIV. 2016;3(10):e482-9.
5. Friedman JR, Abramovitz D, Skaathun B, Rangel G, Harvey-Vera A, Vera CF, et al. Illicit Fentanyl Use and Hepatitis C Virus Seroconversion Among People Who Inject Drugs in Tijuana and San Diego: Results From a Binational Cohort Study. Clinical Infectious Diseases. 2024;79(4):1109-16.
6. Geddes L, Iversen J, Wand H, Esmaeili A, Tsui J, Hellard M, et al. Sex Discrepancies in the Protective Effect of Opioid Agonist Therapy on Incident Hepatitis C Infection. Clinical Infectious Diseases. 2020;70(1):123-31.
7. Hagan H, Thiede H, Des Jarlais DC. Hepatitis C virus infection among injection drug users: survival analysis of time to seroconversion. Epidemiology. 2004;15(5):543-9.
8. Hahn JA, Page-Shafer K, Lum PJ, Bourgois P, Stein E, Evans JL, et al. Hepatitis C virus seroconversion among young injection drug users: relationships and risks. Journal of Infectious Diseases. 2002;186(11):1558-64.
9. Hill LA, Martin NK, Torriani FJ, Jain S, Qin H, Mathews WC, et al. Screening for Sexually Transmitted Infections During Hepatitis C Treatment to Predict Reinfection Among People With HIV. Open Forum Infectious Diseases. 2021;8(2):ofaa643.
10. Jain JP, Abramovitz D, Strathdee SA, Gonzalez-Zuniga P, Rangel G, West BS, et al. Sex Work as a Mediator Between Female Gender and Incident HIV Infection Among People Who Inject Drugs in Tijuana, Mexico. AIDS & Behavior. 2020;24(9):2720-31.
11. Kozlov AP, Shaboltas AV, Toussova OV, Verevochkin SV, Masse BR, Perdue T, et al. HIV incidence and factors associated with HIV acquisition among injection drug users in St Petersburg, Russia. AIDS. 2006;20(6):901-6.
12. Kozlov AP, Skochilov RV, Toussova OV, Verevochkin SV, Krasnoselskikh TV, Malov SV, et al. HIV incidence and behavioral correlates of HIV acquisition in a cohort of injection drug users in St Petersburg, Russia. Medicine (Baltimore). 2016;95(44):e5238.
13. Kral AH, Bluthenthal RN, Lorvick J, Gee L, Bacchetti P, Edlin BR. Sexual transmission of HIV-1 among injection drug users in San Francisco, USA: risk-factor analysis. Lancet. 2001;357(9266):1397-401.
14. Kulchynska R, Sazonova Y, Azarskova M, Salyuk T, Kornilova M, Vitek C, et al. Sex disparities and partners' positive HIV status in HIV recent infection among people who inject drugs in Ukraine. 2026.
15. Lucas GM, Solomon SS, Srikrishnan AK, Agrawal A, Iqbal S, Laeyendecker O, et al. High HIV burden among people who inject drugs in 15 Indian cities. AIDS. 2015;29(5):619-28.

16. Martin M, Vanichseni S, Suntharasamai P, Sangkum U, Mock PA, Leethochawalit M, et al. Risk behaviors and risk factors for HIV infection among participants in the Bangkok tenofovir study, an HIV pre-exposure prophylaxis trial among people who inject drugs. PLoS ONE [Electronic Resource]. 2014;9(3):e92809.
17. Martinello M, Carson JM, Van Der Valk M, Rockstroh JK, Ingiliz P, Hellard M, et al. Reinfection incidence and risk among people treated for recent hepatitis C virus infection. AIDS. 2023;37(12):1883-90.
18. Mehta SH, Galai N, Astemborski J, Celentano DD, Strathdee SA, Vlahov D, et al. HIV incidence among injection drug users in Baltimore, Maryland (1988-2004). Journal of Acquired Immune Deficiency Syndromes. 2006;43(3):368-72.
19. Mirzazadeh A, Evans JL, Hahn JA, Jain J, Briceno A, Shiboski S, et al. Continued Transmission of HIV Among Young Adults Who Inject Drugs in San Francisco: Still Room for Improvement. AIDS Behav. 2018;22(4):1383-94.
20. Moles JP, Vallo R, Khue PM, Huong DT, Oanh KTH, Thoa NT, et al. HIV control programs reduce HIV incidence but not HCV incidence among people who inject drugs in HaiPhong, Vietnam. Sci. 2020;10(1):6999.
21. Read P, Tang BZH, Silins E, Doab A, Cornelisse VJ, Gilliver R. Hepatitis C (HCV) Reinfection and Risk Factors among Clients of a Low-Threshold Primary Healthcare Service for People Who Inject Drugs in Sydney, Australia. Viruses. 2024;16(6):13.
22. Roy E, Alary M, Morissette C, Leclerc P, Boudreau JF, Parent R, et al. High hepatitis C virus prevalence and incidence among Canadian intravenous drug users. International Journal of STD AND AIDS. 2007;18(1):23-7.
23. Roy E, Richer I, Morissette C, Leclerc P, Parent R, Claessens C, et al. Temporal changes in risk factors associated with HIV seroconversion among injection drug users in eastern central Canada. AIDS. 2011;25(15):1897-903.
24. Scheim AI, Nosova E, Knight R, Hayashi K, Kerr T. HIV Incidence Among Men Who Have Sex with Men and Inject Drugs in a Canadian Setting. AIDS & Behavior. 2018;22(12):3957-61.
25. Skaathun B, Strathdee SA, Shrader CH, Nacht CL, Borquez A, Artamonova I, et al. HIV-1 transmission dynamics among people who inject drugs on the US/Mexico border during the COVID-19 pandemic: a prosepective cohort study. Lancet Reg Health Am. 2024;33:100751.
26. Spada E, Rezza G, Garbuglia AR, Lombardo FL, Zuccaro O, Menniti Ippolito F, et al. Incidence and Risk Factors for Hepatitis C Virus Infection among Illicit Drug Users in Italy. Journal of urban health : bulletin of the New York Academy of Medicine. 2018;95(1):99-110.
27. Spittal PM, Pearce ME, Chavoshi N, Christian WM, Moniruzzaman A, Teegee M, et al. The Cedar Project: high incidence of HCV infections in a longitudinal study of young Aboriginal people who use drugs in two Canadian cities. BMC Public Health. 2012;12:632.
28. Strathdee SA, Galai N, Safaiean M, Celentano DD, Vlahov D, Johnson L, et al. Sex differences in risk factors for hiv seroconversion among injection drug users: a 10-year perspective. Arch Intern Med. 2001;161(10):1281-8.
29. Suntharasamai P, Martin M, Vanichseni S, van Griensven F, Mock PA, Pitisuttithum P, et al. Factors associated with incarceration and incident human immunodeficiency virus (HIV) infection among injection drug users participating in an HIV vaccine trial in Bangkok, Thailand, 1999-2003. Addiction. 2009;104(2):235-42.

30. Sypsa V, Vickerman P, Wiessing L, Malliori M, Hatzakis A. High levels of transmission of HCV infection among people who inject drugs in Greece. Journal of Hepatology. 2017;66 (1 Supplement 1):S416.
31. Vallejo F, Barrio G, Brugal MT, Pulido J, Toro C, Sordo L, et al. High hepatitis C virus prevalence and incidence in a community cohort of young heroin injectors in a context of extensive harm reduction programmes. Journal of Epidemiology AND Community Health. 2015;69(6):599-603.
32. Yin L, Qin GM, Ruan YH, Zhang L, Hao QN, Chen XH, et al. A prospective cohort study on human immunodeficiency virus and syphilis seroconversion among injecting drug users. [Chinese]. Zhonghua liu xing bing xue za zhi = Zhonghua liuxingbingxue zazhi. 2006;27(4):293-7.
33. Zhang Y, Shan H, Trizzino J, Ruan Y, Beauchamp G, Masse B, et al. HIV incidence, retention rate, and baseline predictors of HIV incidence and retention in a prospective cohort study of injection drug users in Xinjiang, China. Int J Infect Dis. 2007;11(4):318-23.
